# Multimodal AI for Alzheimer Disease Diagnosis: Systematic Review of Datasets, Models, and Modalities

**DOI:** 10.2196/85414

**Published:** 2026-03-25

**Authors:** Ziwen Yu, Anthony Mulholland, Tianyan Huang, Qiang Liu

**Affiliations:** 1 School of Engineering Mathematics and Technology, University of Bristol, Tankard's Close, Ada Lovelace Building, Bristol, BS8 1TW, United Kingdom, 44 01173746653; 2 Medical Physics and Biomedical Engineering, University College London, London, United Kingdom

**Keywords:** alzheimer’s disease, neurodegenerative disease, multimodal dataset, machine learning, deep learning, multimodal fusion, computer-aided diagnosis, early diagnosis

## Abstract

**Background:**

Early detection of Alzheimer disease (AD) is essential for timely intervention; yet, diagnostic performance varies widely across modalities and datasets. Recent multimodal artificial intelligence (AI) models have made significant progress, but the evidence base remains fragmented due to heterogeneous datasets, modeling frameworks, and reporting quality.

**Objective:**

This systematic review aimed to analyze studies on multimodal AI models for AD diagnosis, prognosis, and risk prediction over 5 years. We evaluated dataset characteristics, modality combinations, modeling strategies, performance metrics, and methodological limitations. We further discuss real-world implications and translational pathways.

**Methods:**

Following PRISMA (Preferred Reporting Items for Systematic Reviews and Meta-Analyses) 2020 guidelines, we systematically searched PubMed, IEEE Xplore, Scopus, ACM Digital Library, Cochrane, and arXiv, with the final datasets last searched on November 15, 2025. Studies applying multimodal machine learning or deep learning to AD, mild cognitive impairment, and dementia outcomes were included, whereas studies using a single modality or lacking sufficient methodological detail were excluded. QUADAS-2 (Revised Quality Assessment of Diagnostic Accuracy Studies tool) assessed risk of bias. Extracted performance results were synthesized across 4 major multimodal dataset families.

**Results:**

A total of 66 studies met the inclusion criteria. Across datasets, multimodal models consistently outperformed single-modal baselines. Alzheimer’s Disease Neuroimaging Initiative–based diagnosis achieved an average accuracy of 92.5% (SD 3.8%), while mild cognitive impairment–conversion models achieved an average area under the curve (AUC) of 0.922 (SD 0.045), and several fusion architectures reported AUCs above 0.95. In contrast, UK Biobank risk-prediction studies reported an average AUC of 0.84 (SD 0.056), and this reflects performance in large, population-based datasets. DementiaBank speech-language studies achieved an average AUC of 0.813 (SD 0.042), and cross-lingual AD detection achieved an accuracy of 77% (SD 6.5%). Self-collected multimodal datasets demonstrated average accuracies around 96% (SD 2.4%), but their generalizability is limited due to small sample sizes and single-center designs.

**Conclusions:**

This systematic review demonstrates that multimodal AI models consistently outperform single-modal models for AD diagnosis, prognosis, and risk prediction by integrating complementary biological, clinical, and behavioral information. Unlike prior reviews, this review provides a unified synthesis across heterogeneous clinical, imaging, genetic, and linguistic datasets, enabling cross-domain comparison of modeling strategies and performance. However, the generalizability of reported performance was limited due to substantial heterogeneity in dataset composition, outcome definitions, and validation, and prevalent risks of bias. By evaluating these factors, this review clarifies where current evidence is robust and where caution is warranted. The findings highlight the need for standardized multimodal benchmarks, transparent evaluation protocols, and clinically grounded model design to enable reliable real-world deployment. Overall, this work advances the field by framing multimodal AI not only as a performance-driven tool but also as a translational framework for equitable, interpretable, and scalable AD diagnosis.

## Introduction

Alzheimer disease (AD) is the most prevalent neurodegenerative disorder and the leading cause of dementia worldwide [1]. With an aging global population, AD has become one of the most costly and deadly diseases of the 21st century, imposing profound emotional, financial, and caregiving burdens on patients, families, and health systems. By 2050, the number of people with AD is projected to rise from 55 million in 2020 to approximately 139 million [1].

The progression of AD includes the preclinical stage, mild cognitive impairment (MCI), and symptomatic stages, with varying degrees of symptom severity. The preclinical stage is a key window for intervention, during which neuropathological changes have commenced, but clinical symptoms remain largely undetectable [2]. Despite advances in awareness and screening, up to 75% of dementia cases remain undiagnosed worldwide, particularly in low- and middle-income countries [3]. This persistent diagnostic gap highlights the need for low-cost, scalable, and accurate early detection tools to enable timely intervention and slow disease progression [4].

Artificial intelligence (AI) has emerged as a promising approach for improving the early detection and management of AD. By systematically integrating and analyzing multimodal data, AI-based diagnostic frameworks offer powerful tools to enhance early detection accuracy and facilitate timely intervention.

Recent work has used transformer-based models to integrate imaging, genetic, and linguistic data. Multimodal transformers combining magnetic resonance imaging (MRI) or positron emission tomography (PET) with clinical features and cognitive assessments have reported improved diagnostic accuracy and interpretability [5-7]. In parallel, GPT-style architectures, BERT (Bidirectional Encoder Representations From Transformers) variants, and domain-adapted language models improve extraction of linguistic and semantic markers linked to early cognitive decline [8,9]. Self-supervised speech models also perform strongly for detecting MCI and early AD from spontaneous speech [10]. Together, these advances reflect a shift toward unified, more interpretable, and clinically translatable multimodal systems that capture both biological and behavioral aspects of AD.

Traditional machine learning (ML) [11], ensemble methods [12], deep learning [13], and reinforcement learning (RL) [14] can perform well on unimodal data, but clinical diagnosis integrates structural and behavioral information [15]. Unimodal AI can therefore diverge from clinical workflows and miss complementary signals (eg, MRI for structural change plus speech features for cognitive decline [16]), increasing the risk of modality-specific overfitting and poorer real-world performance. Accordingly, recent work has shifted toward multimodal integration for AD diagnosis, yet many studies emphasize incremental accuracy gains while underaddressing generalizability, interpretability, and cost-effectiveness needed for adoption. The literature also remains fragmented: recent reviews often cover multimodal clinical phenotyping datasets [17] and multimodal linguistic cognitive-impairment datasets [18] separately, obscuring cross-modal insights such as how imaging and speech biomarkers might jointly improve early detection.

Recent multimodal methods have substantially improved AD detection. However, a comprehensive systematic review that integrates evidence across both clinical and linguistic modalities, fusion strategies, and critically evaluates methodological quality, dataset diversity, and reporting transparency is still lacking. To address these gaps, this review investigates how multimodal models are applied to AD diagnosis, prognosis, and risk prediction and compares performance across different modality combinations and dataset families published between 2019 and 2025. We also examine modeling and fusion strategies alongside validation practices and assess methodological quality and risk of bias using QUADAS-2 (Revised Quality Assessment of Diagnostic Accuracy Studies tool). Furthermore, key multimodal combinations within public datasets are analyzed in relation to their diagnostic performance, and datasets are categorized to evaluate their suitability for AD research and clinical translation. Overall, this review provides a comprehensive synthesis of multimodal AI in AD diagnosis, bridges previously disconnected research streams, and offers practical guidance for future model development and clinical adoption.

## Methods

### Study Design

This review was conducted in accordance with the PRISMA (Preferred Reporting Items for Systematic Reviews and Meta-Analyses) 2020 guidelines [19], with the search procedures reported following PRISMA-S (Preferred Reporting Items for Systematic Reviews and Meta-Analyses literature search extension) [20] and developed using the principles outlined in the Cochrane Handbook [21]. These methods were applied to systematically identify and evaluate studies on computer-aided AD diagnosis, with a particular focus on those using multimodal clinical phenotyping datasets and multimodal linguistic-based cognitive impairment datasets.

### Source of the Study and Search Criteria

We developed and internally reviewed independent search strategies (no external peer review). We manually searched multiple databases to identify AI-driven multimodal approaches for AD diagnosis, rather than using an integrated multidatabase platform. As this review targets methodological advances mainly reported in peer-reviewed computational literature, we did not search trial registries (ClinicalTrials.gov, World Health Organization’s International Clinical Trials Registry Platform). We also avoided validated or published filters, instead iteratively refining customized controlled-vocabulary and free-text terms for AD or dementia, multimodal data, and AI through pilot screening to maximize sensitivity.

Searches were performed in PubMed (447 records; January 1, 2019, to November 13, 2025), Scopus (1086 records; all years through November 13, 2025, filtered to PUBYEAR > 2018), IEEE Xplore (2229 records; January 1, 2020 to November 13, 2025), ACM Digital Library (2067 records; 1 January 2020, to 15 November 2025), Cochrane Library (1061 records; all available years through November 15, 2025), and arXiv (1081 records; all available years through November 15, 2025). We included the verbatim search strings for all databases, and because arXiv does not support bulk export, an arXiv search Python (Python Software Foundation) script is provided in Multimedia Appendix 1 [11,14,22-44].

### Eligibility Criteria

The inclusion criteria were if studies were considered eligible if they met all the following conditions: (1) focused on AD, MCI, or related dementias as the primary clinical outcome; (2) applied AI or ML methods for computer-aided diagnosis, classification, or prediction; (3) used multimodal data, defined as any combination of at least two distinct modalities (eg, neuroimaging, clinical phenotyping, genetics, or linguistic features); (4) reported quantitative evaluation metrics; and (5) written in English.

The exclusion criteria were if studies met any of the following conditions: (1) single-modal approaches using only a single imaging modality, cognitive test, or biomarker, without any multimodal integration; (2) works without reported performance metrics or with insufficient methodological detail; (3) works not addressing diagnosis, classification, or prediction (eg, treatment response, drug trials, and lifestyle interventions); (4) duplicate publications or overlapping datasets without providing additional methodological contribution; and (5) non-English publications.

### Selection Process

The study selection process followed the PRISMA 2020 guidelines, and the protocol was registered. The final search update was conducted in November 2025. All records retrieved from the databases were first imported into Zotero, where duplicates were automatically detected and removed.

The initial search identified 7435 records. After removing 3047 duplicates, 4388 records remained for title and abstract screening. A total of 4021 records were obviously irrelevant at the title and abstract level, 252 studies were excluded for the following main reasons:

- Focused on outcomes unrelated to AD diagnosis, classification, or prediction (eg, drug trials, treatment response, lifestyle interventions; n=140).
- Used unimodal data without multimodal integration (n=46).
- No sufficient methodological details (n=47).

Finally, 66 studies were included in the systematic synthesis, and all were successfully retrieved (reports not retrieved=0).

### Overview of AI-Assisted AD Diagnosis

**Figure 1.**
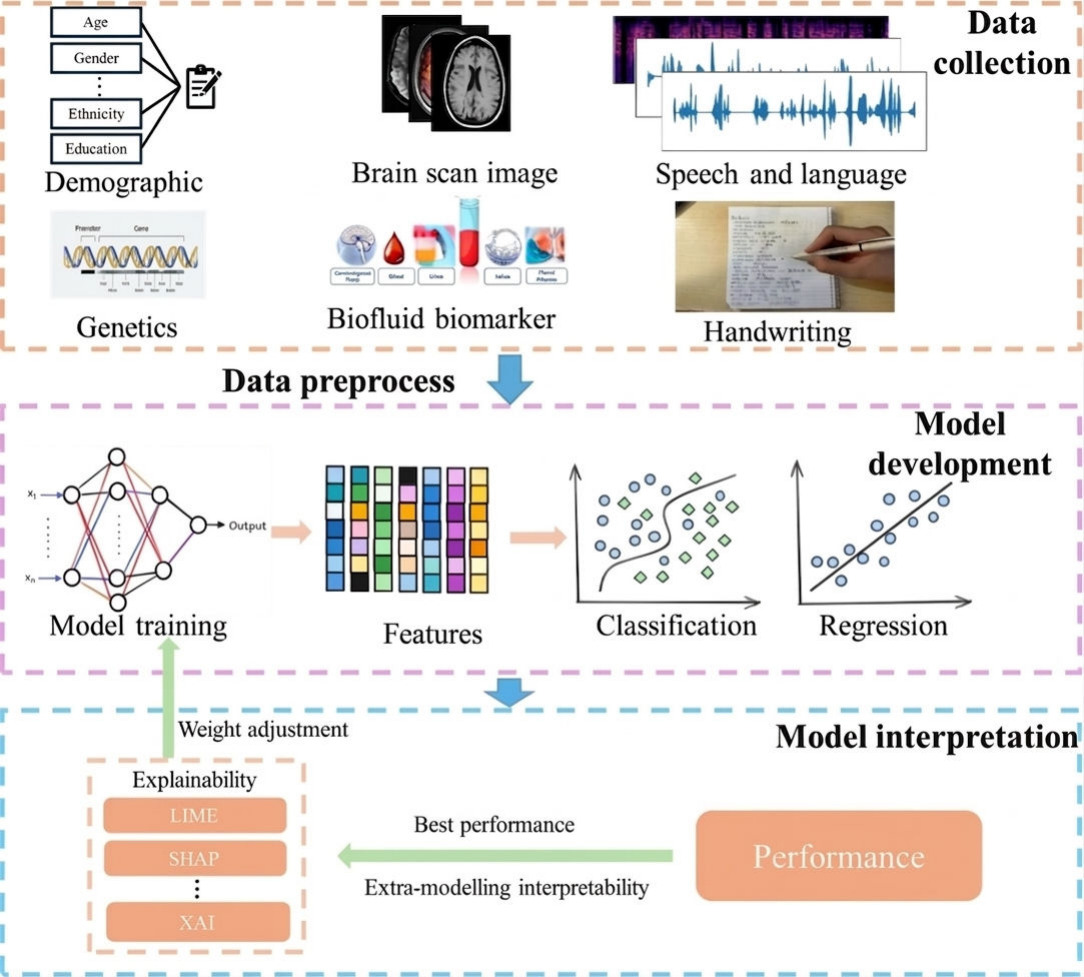
Overview of the AI pipeline for AD diagnosis. Multimodal inputs undergo preprocessing and feature extraction before model training for classification or regression tasks. Model interpretability supports explanation and performance evaluation. AD: Alzheimer disease; AI: artificial intelligence; LIME: Local Interpretable Model-Agnostic Explanations; SHAP: Shapley Additive Explanations; XAI: explainable artificial intelligence.

The workflow of AI-assisted AD diagnosis involves 3 stages, as illustrated in Figure 1. The initial stage involves comprehensive data acquisition, where information is collected from multiple modalities, including neuroimaging, biomarkers, genetics, and speech or behavioral signals. The second stage involves feature extraction and model development, followed by an interpretable and explainable analysis to ensure that AI models can effectively support clinical decision-making.

### Performance Evaluation Metrics

To ensure the clinical applicability and scientific rigor of computer-aided diagnosis models for AD, it is essential to systematically evaluate their performance using a variety of quantitative metrics. We have summarized all performance evaluation metrics in Multimedia Appendix 2.

### Risk of Bias and Quality Assessment

**Figure 2.**
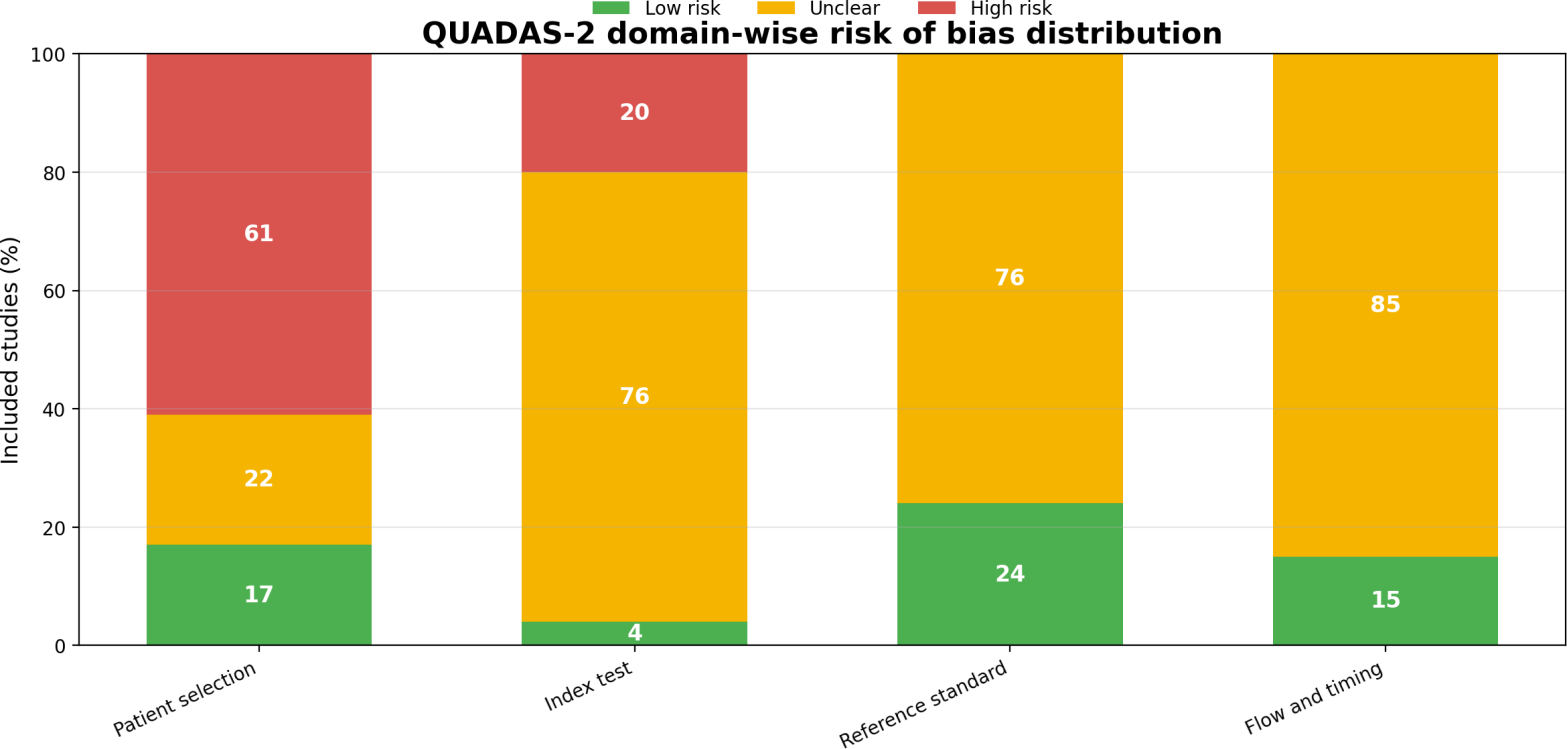
Summary of the QUADAS-2 plot across the 66 included studies in the domain. QUADAS-2: Revised Quality Assessment of Diagnostic Accuracy Studies tool.

We assessed methodological quality with QUADAS-2 [45], evaluating risk of bias in 4 domains. Patient selection raised the main concern: 61% (40/66) of outcomes were high risk due to poor reporting or nonrepresentative sampling. For the index test, 76% (50/66) were unclear risk because procedures and decision thresholds were insufficiently described, and 20% (13/66) were high risk. The reference standard showed 76% (50/66) unclear risk from limited methodological detail, with no high-risk ratings. Flow and timing had the greatest uncertainty: 85% (56/66) were unclear owing to missing information on testing intervals and participant flow. Figure 2 summarizes domain-level risk distributions; Multimedia Appendix 3 reports study-level assessments.

Given frequent unclear and high risk in key domains, we interpreted diagnostic performance cautiously, especially without external validation or a clearly defined reference standard. Future benchmarking should emphasize transparent reporting, prespecified thresholds, and multicenter evaluation to reduce bias and improve reproducibility.

## Results

### Overview

**Figure 3.**
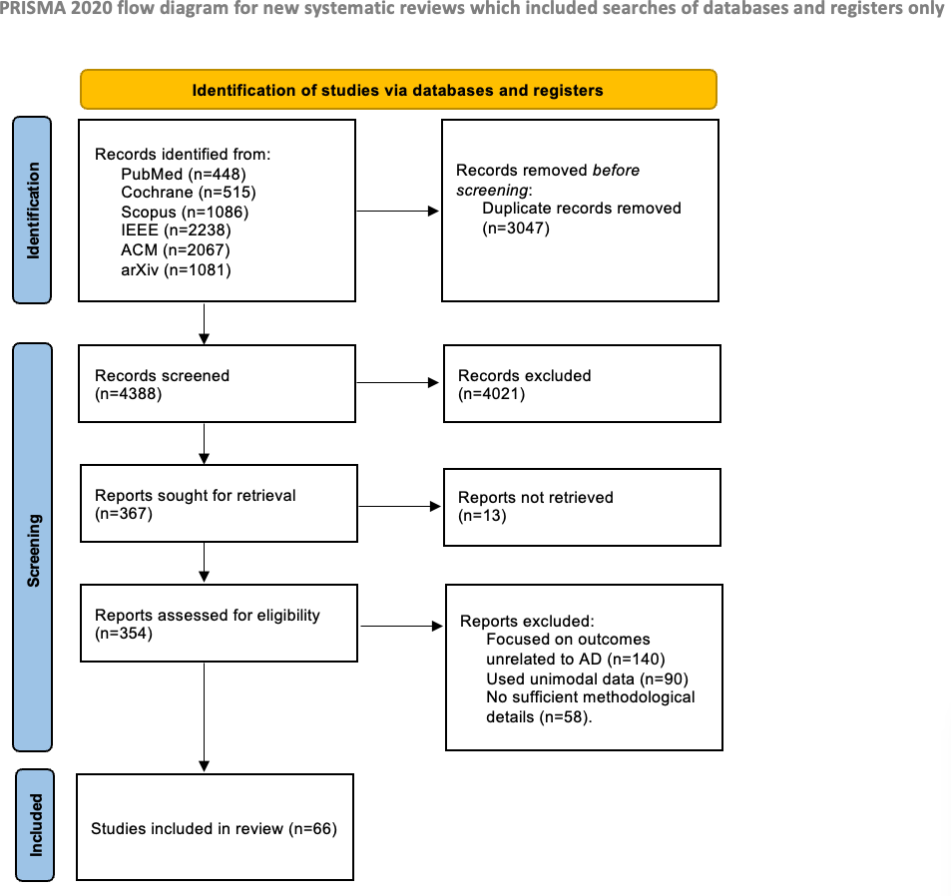
Flow diagram of PRISMA. AD: Alzheimer disease; PRISMA: Preferred Reporting Items for Systematic Reviews and Meta-Analyses.

**Figure 4.**
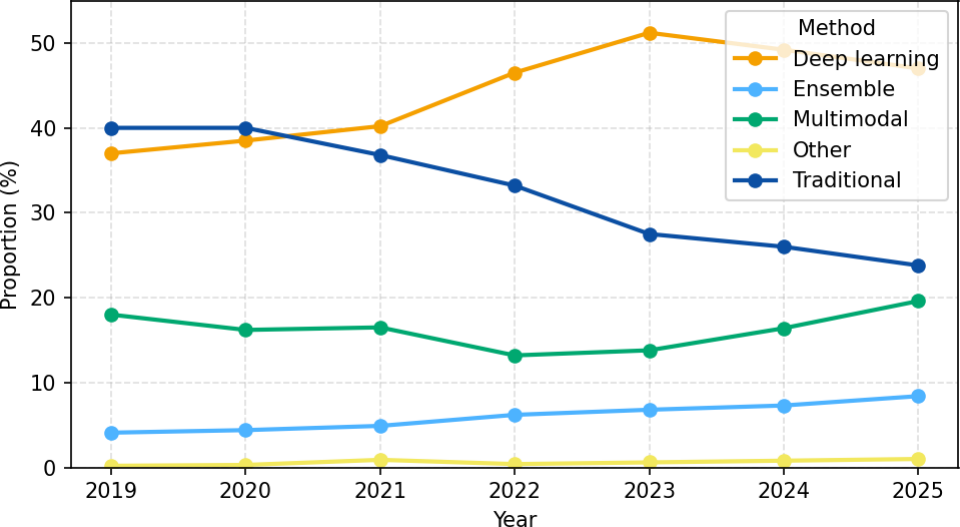
Temporal trends of machine-learning methods used for AD diagnosis (2019‐2025). AD: Alzheimer disease.

Following study selection (the complete selection process is summarized in the PRISMA 2020 flow diagram, Figure 3), we first summarize the overall profile of the included literature to contextualize the subsequent synthesis. Figure 4 provides a temporal overview (2019‐2025) of modeling approaches across included studies, illustrating how methodological focus has shifted over time and informing interpretation of the evidence base.

### Single Modality

#### Overview

If readers are already familiar with traditional ML and deep learning approaches, they may wish to proceed directly to the next section, which focuses on multimodal data integration for AD diagnosis.

A concise overview of these baseline methods is provided in Multimedia Appendix 4, which also includes a summary of RL. As most RL studies address sequential decision-making tasks rather than direct diagnostic modeling, their methodological details are presented in Multimedia Appendix 4, to maintain focus on multimodal diagnostic frameworks in the main text.

#### Deep Learning

Compared with traditional ML, deep learning enables hierarchical feature extraction, capturing complex patterns in high-dimensional data. It is therefore widely used to process and integrate AD-related multimodal inputs, including neuroimaging, clinical scores, genetics, and speech. Key approaches and findings are summarized below.

Recurrent neural networks are effective for modeling sequential data such as longitudinal clinical records and speech signals, but they are susceptible to vanishing gradients in long sequences [46-49]. Long short-term memory networks address this limitation through gated memory mechanisms, enabling more stable training and improved capture of long-term dependencies. Consequently, long short-term memory models have been widely applied in AD research for analyzing temporal and sequential modalities [50-53].

The transformer model, which leverages attention mechanisms, dynamically assigns different weights to input features based on their relative importance. Each layer of the transformer consists of multiple attention heads, allowing the model to capture diverse feature representations by attending to various aspects. Transformer models use attention mechanisms to weight input features and capture diverse representations through multihead attention, enabling efficient and scalable training [54]. Owing to these advantages, they have been widely adopted in AD diagnosis and multimodal learning, where their encoder-decoder architecture facilitates effective integration of heterogeneous data sources [13,55-57].

### Ensemble Learning

Ensemble learning improves generalization and robustness by combining multiple base models, including bagging and boosting methods such as AdaBoost (Adaptive Boosting), XGBoost (Extreme Gradient Boosting), and LightGBM (Light Gradient-Boosting Machine), and has been widely applied in AD detection and progression prediction [12,58-60]. However, ensemble models may introduce redundant features, offer limited gains on small datasets, and incur higher computational costs, which can restrict real-time or resource-constrained deployment.

### Summarization for Single Modality

Traditional single-modality ML approaches can achieve high performance in AD-related tasks; however, they are constrained by several inherent limitations:

First, regarding information completeness, structural MRI alone has limited sensitivity to functional and molecular changes and cannot fully capture AD-related cognitive and behavioral alterations. Combining MRI with PET, neuropsychological tests, speech, electroencephalography (EEG), and genetic or biomarker data provides a more complete, multidimensional view of disease progression and patient heterogeneity [61].

Second, regarding model robustness, in multimodal data, residual noise in one modality may persist despite denoising, but other modalities can provide complementary signals that improve robustness. Leveraging multisensory-style integration, multimodal models better reflect biological cognition and can yield more reliable decisions [62].

Third, in cross-modal learning, transformer architectures use cross-modal attention to learn associations between modalities. Some studies apply them in weakly supervised or cross-modal guided settings, using one modality to constrain or guide representation learning in another [63].

Fourth, in real-world decision-making, multimodal learning better matches real-world diagnosis, which integrates multiple information sources. Using diverse modalities aligns models with clinical workflows and improves translational potential in practice [64].

Therefore, this review analyzes the methodological strengths and limitations of multimodal models for computer-assisted AD diagnosis, focusing on how dataset grouping and classification choices affect evaluation. By classifying datasets, we enable model comparisons under a unified setup, allowing for more direct assessment of generalizability and cross-dataset stability.

### Multimodal Data

#### Multimodal Dataset Overview

High-quality data plays a pivotal role in training AI models for computer-aided diagnosis and detection. Robust datasets not only enhance the generalization ability of models but also help mitigate the risk of overfitting. Commonly used datasets for AI-assisted diagnosis of AD can be broadly categorized into 2 types. The first type of multimodal clinical phenotyping dataset, such as the Alzheimer’s Disease Neuroimaging Initiative (ADNI), UK Biobank, and the Open Access Series of Imaging Studies (OASIS), focuses on neuroimaging modalities, including MRI, functional MRI, genetic data, and electronic health records. The second type—multimodal cognitive-linguistic behavioral dataset centers on sequential data modalities, including audio, video, and transcribed language data, such as the Pitt Corpus and ADReSS (Alzheimer’s Dementia Recognition Through Spontaneous Speech). Each dataset type provides unique features that contribute to the comprehensive modeling of AD progression and diagnosis. The commonly used datasets, along with their population demographics and associated modalities, are summarized in Table 1.

**Table 1.** Commonly used dataset.

Dataset		Population demographics		Modalities	Link
		Male	Age (years)		
UK Biobank		23,000 (46)^a^	56.5 (8.1)^b^	MRI^c^, fMRI^d^, genetic, lifestyle scores, activity monitor, and EHR^e^	[65]
ADNI^f^					
ADNI-1		469 (57.3)^a^	75 (6.9)^b^	MRI, PET^g^, genetic, and EHR	[66]
ADNI-GO^h^ and 2		473 (53)^a^	72.5 (7.2)^b^	MRI, PET, genetic, and EHR	[66]
ADNI-3		471 (49)^a^	74.9 (8.1)^b^	MRI, PET, genetic, and EHR	[66]
OASIS^i^					
OASIS-1		177 (42.5)^a^	57 (39)^b^	MRI, PET, CT, genetic, and EHR	[67]
OASIS-2		60 (40)^a^	78 (18)^b^	MRI, PET, CT, genetic, and EHR	[67]
OASIS-3		622 (45)^a^	69 (26.56)^b^	MRI, PET, CT, genetic, and EHR	[67]
OASIS-4		663 subjects	57.5 (36.5)^b^	MRI, PET, CT, genetic, and EHR	[67]
NACC^j^		23,625 (44.62)^a^	73.3 (10.5)^b^	MRI, PET, genetics, and EHR	[68]
FHS^k^		718 (42)^a^	80.76 (8.2)^b^	MRI, genetic, and EHR	[69]
AIBL^l^		289 (43.72)^a^	73.5 (7.03)^b^	MRI, PET, genetic, and EHR	[70]
Dementia bank					
Pitt corpus		HC^m^: 104, AD^n^: 208/552	—^o^	Audio and text	[71]
ADReSS^p^		HC: 78, AD: 78/156	—	Audio and text	[71]
ADReSSo^q^		HC: 115, AD: 122/237	—	Audio and text	[71]
ADReSS-M^r^		HC: 143, AD: 148/291	—	Audio and text	[71]
TAUKADIAL^s^		106/507	—	Audio and text	[71]
Multimodal dementia corpus		HC: 10, AD: 12/816	—	Audio, typed, and hand-written	[72]
ADReFV^t^		AD: 25	66.68 (2.08)^b^	Video	[73]
GENCODE^u^		Human: 78,686	—	Genetics	[74]

a n (%).

b Mean (SD).

c MRI: magnetic resonance imaging.

d fMRI: functional magnetic resonance imaging.

e EHR: electronic health record.

f ADNI: Alzheimer’s Disease Neuroimaging Initiative.

g PET: positron emission tomography.

h ADNI-GO: Alzheimer’s Disease Neuroimaging Initiative – Grand Opportunity.

i OASIS: Open Access Series of Imaging Studies.

j NACC: National Alzheimer’s Coordinating Centre.

k FHS: Framingham Heart Study.

l AIBL: Australian Imaging, Biomarkers and Lifestyle Study.

m HC: health control.

n AD: Alzheimer disease.

o Not available*.*

p ADReSS: Alzheimer’s Dementia Recognition Through Spontaneous Speech.

q ADReSSo: Alzheimer’s Dementia Recognition Through Spontaneous Speech 2021 Challenge.

r ADReSS-M: Multilingual Alzheimer’s Dementia Recognition Through Spontaneous Speech Challenge.

s TAUKADIAL: Speech-Based Cognitive Assessment in Chinese and English.

t ADReFV: Alzheimer’s Disease Recognition from Face & Voice.

u GENCODE: gene code.

To better understand current research trends, this paper reviewed studies from the past 5 years on multimodal AI models for AD diagnosis. The literature was categorized by dataset type. (1) Multimodal clinical phenotyping datasets: ADNI dominates this category, used in about 80% of studies, while others, such as UK Biobank, OASIS, National Alzheimer’s Coordinating Centre (NACC), Framingham Heart Study, and Australian Imaging, Biomarkers and Lifestyle Study, account for the remaining 20%, mainly for supplementary analysis or external validation. (2) Multimodal cognitive-linguistic behavioral datasets: The ADReSS series is most widely used, representing around 70% of studies. The remaining 30% use datasets often for complementary analysis or benchmarking.

#### Multimodal Clinical Phenotyping Datasets

Multimodal clinical phenotyping datasets integrate MRI, PET, or diffusion tensor imaging, biomarkers from blood, cerebrospinal fluid, or genomics, and standardized cognitive assessments. This review summarizes representative resources, highlighting their modalities, distinguishing features, and contributions to diagnostic modeling (Table 2).

**Table 2.** Multimodal clinical phenotyping datasets related paper. The exceptionally high performances reported in some of these studies can be attributed to specific methodological factors: (1) for two studies [75] and [76], the absence of external validation likely inflated the results; (2) for another study [77], the use of a small but highly controlled dataset, extensive sample expansion, multimodal feature fusion, and pronounced disease-related electrophysiological signatures contributed to the elevated accuracy; and (3) for yet another study [78], the integration of rich gait features with optimized machine learning techniques in a controlled experimental setting facilitated unusually high performance.

Study	Datasets	Model type	Type of task	Modalities	Outcomes	Validation	Results	Limitation
Xue et al [79], 2024	NACC^a^, ADNI^b^, AIBL^c^, FHS^d^, PPMI^e^, and OASIS^f^	Transformer-based multimodal model	Differential diagnosis of 10 etiologies	MRI^g^ (T1, T2, FLAIR^h^), clinical, neuropsychological tests, and PET^i^	Differential diagnosis probabilities and AD^j^/MCI^k^/NC^l^ classification	Internal: NACC held out; external: ADNI and FHS	Etiology classification AUROC^m^ 0.96; strong alignment with PET biomarkers and neuropathology	Imbalanced etiologies, training label subjectivity, and limited racial diversity
Shi et al [80], 2018	ADNI	MM-SDPN^n^	Classification (AD vs NC, MCI vs NC)	T1 MRI and FDG-PET^o^	Classification accuracy	Comparative vs single-modality and state-of-the-art models	Outperformed single-modality DPN^p^/SDPN^q^ and concatenated models	ADNI-only dataset (limits generalizability); ROI^r^-based features rather than voxel-wise
Allwright et al [81], 2023	UK Biobank	XGBoost^s^	Risk prediction (Incident AD)	Demographics, lifestyle, genetics, and medical history	Prediction of incident AD (2-10 years) and risk factor ranking	Internal: nested 3-fold cross-validation; external: held-out validation set	AUROC 0.77, APOE-ε4^t^ identified as the strongest risk factor, and liver enzymes or frailty as predictors	*ICD-10*^u^ underascertainment, healthy volunteer bias, and observational design
Gu et al [82], 2025	UK Biobank	LightGBM^v^	Risk prediction (incident dementia in ASCVD^w^ patients)	Clinical, biological assays, cognitive tests, and physical measures	All-cause incident dementia, AD, and VD^x^ incidence	Temporal: train (2006-2009) and test (2010 cohort)	5-year dementia AUC^y^ 0.903, AD AUC 0.775, and accuracy 0.851	Sample mostly European descent, static baseline features, and potential overfitting
You et al [83], 2022	UK Biobank	LightGBM	Risk prediction (5/10-year horizon)	Demographics, lifestyle, blood biomarkers, and genetics	Incident all-cause dementia and AD prediction	Internal: 5-fold cross-validation	AUC 0.848 (all-cause), 0.862 (AD), and outperformed CAIDE^z^ and DRS^aa^	Limited external validation, population predominantly White, and feature selection fully data-driven
Calvo et al [84], 2024	UK Biobank	Multivariable logistic regression	Risk association analysis	Questionnaire, ICD^ab^ records, and genotypes	Odds of AD related to menopause type	Single cohort: multivariable adjustment	Early bilateral oophorectomy associated with 4-fold AD odds (OR^ac^ 4.12) and HT^ad^ use protective	Low case numbers in subgroups; self-reported HT use and healthy volunteer bias
Yi et al [85], 2025	UK Biobank, ADNI, PPMI, and IXI^ae^	3D-ViT^af^	BAG^ag^ estimation and GWAS^ah^	T1-weighted MRI, genetics (SNP^ai^ and xQTL^aj^)	BAG and drug target prioritization	External: ADNI, PPMI, and IXI	MAE^ak^ ≈ 2.6 and identified 7 high-confidence drug targets (eg, MAPT^al^ and TNFSF12^am^)	European-ancestry bias; lack of biological “ground truth” for brain age
Yousefzadeh et al [86], 2024	UK Biobank (retina cohort)	VGG-16^an^ classifier + LAVA^ao^ (XAI^ap^)	Binary classification and explainability	Retinal fundus images	AD vs NC classification and neuron-level explanations	Internal: nested 5-fold cross-validation	Accuracy 71.4% and identified 7 latent clusters linking vascular and cognitive decline	Small AD sample size (n=100), cross-sectional design, and UK Biobank volunteer bias
Gong et al [87], 2023	UK Biobank	SuperBigFLICA (semisupervised Bayesian fusion)	Phenotype discovery	Multimodal MRI (47 modalities)	Latent components predictive of nonimaging phenotypes	Internal: train, validation, or test split	Up to 46% improvement over expert IDPs^aq^ and interpretable multimodal modes	Linear modeling constraints and UK Biobank population bias
Lian et al [88], 2022	ADNI-1, ADNI-2, and AIBL	Attention-guided HybNet (3D FCN^ar^ + hybrid network)	Diagnosis and prognosis	Structural T1 MRI	AD vs NC classification; pMCI^as^ vs sMCI^at^ prediction	External: trained ADNI-1, and validated ADNI-2 and AIBL	ADNI-2 AD vs NC accuracy 0.919 (AUC 0.965) and outperformed ROI/VBM^au^ methods	Heavy preprocessing reliance, limited demographic diversity, and potential overfitting
Lian et al [89], 2022	ADNI-1 and ADNI-2	MWAN^av^	Joint regression of clinical scores	Structural T1 MRI and clinical scores	MMSE^aw^, CDRSB^ax^, and ADAS-Cog^ay^ prediction	Cross-validation: across ADNI-1 and ADNI-2	Lower RMSE^az^ and higher correlation coefficients than single-task baselines	Restricted to the ADNI cohorts and potential overfitting to the modest sample size
Li et al [90], 2019	ADNI-1, ADNI-GO/2^ba^, and AIBL	3D CNN^bb^ + Cox proportional hazards	Time-to-event prognosis	Hippocampal MRI patches and clinical variables	Progression from MCI to AD and risk stratification	External: trained ADNI-1, and validated ADNI-GO/2 and AIBL	C-index 0.864 (combined model) and significant risk-based stratification of MCI	Focus on the hippocampus only and potential cohort and scanner bias (1.5T vs 3T)
Qiu et al [15], 2022	NACC, ADNI, and ADCP^bc^	Multimodal deep learning (3D CNN + FCN)	Multiclass classification	Structural MRI, demographics, and neuropsychology	Diagnosis (NC, MCI, AD, and nADD^bd^) and saliency maps	External: trained NACC, and validated ADNI and independent cohorts	Performance comparable to neurologists and saliency aligned with pathology	Retrospective design and heterogeneity in protocols across cohorts
Oh et al [91], 2023	ADNI	LEAR^be^ framework (CNN + RL^bf^ + XAI)	Diagnosis and interpretation	Structural T1 MRI	AD vs non-AD classification and counterfactual maps	Internal: cross-validation on ADNI	Improved accuracy and generalization, and localized plausible atrophy patterns	Single-cohort (ADNI) and XAI evaluation, partly qualitative
Lian et al [92], 2020	ADNI-1 and ADNI-2	Hierarchical FCN	Diagnosis and atrophy localization	Structural T1 MRI	AD vs NC, MCI vs NC, and atrophy pattern mapping	External: trained ADNI-1 and tested ADNI-2	Improved accuracy vs conventional features and interpretable atrophy maps	ADNI-only; strong reliance on preprocessing and registration
Avsec et al [93], 2021	Genomic reference datasets	Enformer (transformer)	Genomic prediction	DNA sequence	Gene expression and chromatin state prediction	Internal: held-out chromosomes	Improved capture of long-range regulatory effects vs previous models	Limited to available cell types and assays
Yang et al [94], 2021	ADNI	Deep learning and super learner	Prognosis	MRI, cognitive, and biomarkers	Diagnostic classification and prognostic risk signature	Internal: cross-validation within ADNI	Derived signature distinguished diagnostic groups and progression risk	Limited external validation and restricted to the ADNI research cohort
Lee et al [95], 2024	ADNI and UK or Singapore Clinics	PPM^bg^	Prognosis (MCI to AD)	MRI (gray matter) and cognitive tests	Individualized prognostic index	External: independent real-world memory clinics	Accuracy ≈81.7%, AUC ≈0.84; and index predicted conversion better than atrophy alone	Heterogeneity in real-world clinical data and potential site effects
Zhu et al [96], 2021	ADNI and AIBL	DA-MIDL^bh^	Diagnosis	Structural MRI patches	AD vs NC and MCI vs NC	External: trained ADNI and tested AIBL	Higher accuracy and generalizability than baselines, and attention maps aligned with pathology	Reliance on structural MRI and potential dataset-specific overfitting
Zhang et al [97], 2024	ADNI	GCN^bi^, SHAP^bj^, and automatic fusion	Diagnosis	Cognitive, MRI, PET, and risk factors	AD vs non-AD diagnosis, and multimodal feature selection	Internal: two ADNI multimodal cohorts	Accuracies of 95.9% and 91.9%, and efficient selection of clinically important features	Complex model deployment and reliance on ADNI data
Velazquez and Lee [75], 2022	ADNI EMCI^bk^	Ensemble (random forest and CNN)	Prediction of conversion	DTI^bl^ (ADC^bm^ maps) and EHR^bn^	EMCI to AD conversion prediction	Internal: held-out test set	98.81% accuracy and feature importance explainability provided	Small converter sample size and potential overfitting
Zhang et al [76], 2024	ADNI	Multimodal learning machine (ELM^bo^ ensemble)	Diagnosis	MRI features and neuropsychological tests	NC, MCI, and AD classification	Internal: cross-validation on ADNI	>98% accuracy and *F*_1_-score, and no observed bias between MCI and AD	A single research cohort and very high accuracy require external verification
Bi et al [98], 2020	ADNI	Cluster evolutionary random forest	Diagnosis	Resting-state fMRI^bp^ and SNP	AD vs control classification and biomarker identification	Comparative vs competing methods	Identified significant brain region-gene pairs and effective classification	Small multimodal sample size and complex hyperparameters
Bi et al [99], 2022	ADNI	Weighted evolutionary random forest	Pathogen detection	Resting-state fMRI and SNP	MCI identification and pathogenic factor extraction	Comparative vs state-of-the-art methods	Superior MCI identification performance, and highlighted key ROIs and SNPs	High-dimensional fusion features, small N, and overfitting risk
Hashmi and Barukab [100], 2023	OASIS	Deep RL and neural network	Staging	Structural MRI	4-class dementia staging	Internal: augmented vs baseline	RL augmentation improved accuracy by ≈6% and recall by ≈13%	Single open dataset; focus on MRI only
Wang et al [101], 2024	ADNI-1/2/3	Multimodal DL^bq^ with an interaction layer	Prognosis (MCI to AD)	MRI, clinical, and genetics (SNP)	4-year conversion prediction	External: generalized to ADNI-3	AUC 0.962 (cross-validation), 0.939 (test); interaction effects improved accuracy	ADNI-only and potential overfitting despite cross-validation
Hatami et al [102], 2024	ADNI	DNN^br^ and RL (data augmentation)	Classification	Structural MRI	AD vs NC classification	Comparative vs baseline augmentation approaches	Precision ≈0.95; RL-guided augmentation outperformed baselines	Single research cohort and no external clinical validation
Tabarestani et al [103], 2020	ADNI	Distributed multitask regression	Longitudinal progression	MRI, PET, CSF^bs^, EEG^bt^, and clinical	Prediction of longitudinal cognitive scores	Comparative vs unimodal or multimodal methods	Reduced errors, particularly in sparse or incomplete longitudinal data	Model complexity and potential sensitivity to hyperparameters
Burkhart et al [104], 2024	ADNI and Singapore Memory Clinic	Unsupervised multimodal trajectory modeling	Prognosis	Cognitive, amyloid PET, and MRI	Cognitive health clustering and progression prediction	External: real-world memory clinic data	Better stratification than standard clinical assessments and robust to missing data	Unsupervised complexity and reliance on ADNI for training
El-Sappagh et al [105], 2021	ADNI	Random forest and SHAP (multilayer)	Diagnosis and progression	11 modalities (MRI, PET, CSF, and clinical)	Multiclass diagnosis and MCI progression detection	Internal: cross-validation	Diagnosis accuracy 93.95%, progression accuracy 87.08%, and interpretable	High complexity and challenges for routine care deployment
Lee et al [106], 2024	ADNI and 4 Korean hospitals	GBM^bu^	Conversion prediction	MRI (T1, T2-FLAIR^bv^), amyloid PET, and clinical	MCI to AD conversion (4-year)	Internal: nested cross-validation with modality combinations	T1 and amyloid PET is the best combination, and T2-FLAIR did not improve prediction	Small multicenter sample and site and scanner heterogeneity
Yuan et al [107], 2021	ADNI	Multimodal cotraining (random forest)	MCI subtype classification	Structural MRI and SNP	sMCI vs pMCI classification	External: ADNI-2 independent test set	Accuracy 85.5% and cotraining outperformed single modality	Dependence on feature selection and ADNI-only
Cirincione et al [108], 2024	TADPOLE^bw^ (ADNI)	Ensemble integration	Prediction	MRI, PET, clinical, and cognitive	Future dementia prediction in MCI	Internal: held-out test set	AUC 0.81, and outperformed XGBoost and deep learning baselines	Single research dataset and the complexity of multimodal integration
Cassani and Falk [109], 2020	Clinical EEG	Feature engineering and ML	Diagnosis and severity	Resting-state EEG	AD vs normal, and mild vs moderate AD classification	Internal: cross-validation	Modulation spectral features outperformed traditional EEG features	Small sample size, resting state only, and single center
Cilia et al [110], 2021	Custom (Naples)	Deep transfer learning (CNN)	Diagnosis	Online handwriting (dynamic)	Early AD detection	Internal: cross-validation	Dynamic features (color-encoded) are superior to shape-only images	Single-center dataset and task-specific protocol
Kmetzsch et al [111], 2022	PREV-DEMALS^bx^	Supervised variational autoencoder	Disease progression modeling	MRI and microRNA	Disease progression score (FTD^by^/ALS^bz^)	Validation: synthetic data and cohort evaluation	Outperformed competing models in capturing progression trajectory	Small sample (rare disease) and cross-sectional data used for progression
Mengoudi et al [112], 2020	UCL^ca^ and Insight 46	Self-supervised deep neural network	Diagnosis	Eye-tracking (gaze or pupil)	Dementia vs control classification	Comparative vs handcrafted features	Self-supervised features are more sensitive than handcrafted metrics	Modest sample size, mixed dementia subtypes, and specialized hardware
Tsai et al [113], 2024	Taiwan NHI^cb^	MAND^cc^	Incidence prediction	EHR (ICD codes) and demographics	Dementia incidence risk	Internal: held-out test set	AUC 0.901 and outperformed traditional CTR^cd^ models	Coding errors in administrative data are specific to the Taiwan NHI
Park et al [22], 2024	Korean memory clinics	SVM^ce^	Diagnosis (MCI vs HC^cf^)	VR^cg^ biomarkers, MRI, and neuropsychological tests	MCI vs healthy control classification	Internal: train or test split	VR, MRI AUC 0.89, and VR biomarkers comparable to MRI alone	Small sample (n=54) and VR hardware requirement
Wu et al [114], 2022	Clinical EEG	WiGMM^ch^	Severity detection	Resting-state EEG	Unsupervised dementia degree detection	Internal: latent structure analysis	Captured latent dementia degrees matching clinical status	Unsupervised labeling requires careful interpretation
Zhang et al [115], 2025	Chinese memory clinics	FCRN^ci^ and MLP^cj^ (patch-based)	Diagnosis	MRI, PET, clinical, and genotype	AD vs normal and MCI vs normal classification	Internal: cross-validation	Accuracy ≈96% (AD), ≈92% (MCI), and interpretable probability maps	Single-country clinical cohorts and limited ethnic diversity
Fabietti et al [77], 2023	Mouse models	Ensemble machine learning	Early detection (animal)	LFP^ck^	AD vs control mouse classification	Internal: channel masking robustness tests	Accuracy 99.4% and robust to artifacts	Preclinical animal model results and small sample size
Seifallahi et al [78], 2022	Single center	SVM	Diagnosis	Kinect V2 (gait or TUG^cl^)	AD vs healthy control classification	Internal: leave-one-out cross-validation	Accuracy 98.68% using 12 skeletal features	Small sample, case-control design may overestimate performance
Fan et al [116], 2024	CVD^cm^ patients (Wuhan)	ViT^cn^ (MRI) and XGBoost (clinical)	VCI^co^ diagnosis	MRI (T1, T2-FLAIR) and clinical	Vascular cognitive impairment diagnosis	External: independent CVD dataset	The hybrid model has an AUC of 0.965 and is comparable to expert neurologists	CVD-specific cohort and complex ViT and XGBoost pipeline
Beebe-Wang et al [117], 2021	Aging cohort (US)	Nonlinear ML and SHAP	Imminent prediction (3 years)	Clinical, neuropsychological	Incident dementia within 3 years	Internal: cross-validation	Sparse model (4 tests) comparable to full battery	Prediction limited to a 3-year horizon and a single health system
Battineni et al [118], 2021	Public MRI dataset	Gradient boosting	Classification	MRI features and demographics	AD vs non-AD classification	Internal: cross-validation	Accuracy 97.58% (gradient boosting performed best)	Small public dataset and lack of external validation

a NACC: National Alzheimer’s Coordinating Centre.

b ADNI: Alzheimer’s Disease Neuroimaging Initiative.

c AIBL: Australian Imaging, Biomarkers, and Lifestyle Study.

d FHS: Framingham Heart Study.

e PPMI: Parkinson Progression Markers Initiative.

f OASIS: Open Access Series of Imaging Studies.

g MRI: magnetic resonance imaging.

h FLAIR: fluid-attenuated inversion recovery.

i PET: positron emission tomography.

j AD: Alzheimer disease.

k MCI: mild cognitive impairment.

l NC: normal control.

m AUROC: area under the receiver operating characteristic curve.

n MM-SPDN: multimodal stacked deep polynomial network.

o FDG-PET: fluorodeoxyglucose-positron emission tomography.

p DPN: deep polynomial network.

q SPDN: stacked deep polynomial network.

r ROI: region of interest.

s XGBoost: Extreme Gradient Boosting.

t APOE-ε4: apolipoprotein E epsilon 4 allele.

u *ICD-10*: *International Statistical Classification of Diseases, Tenth Revision.*

v LightGBM: Light Gradient-Boosting Machine.

w ASCVD: atherosclerotic cardiovascular disease.

x VD: vascular dementia.

y AUC: area under the curve.

z CAIDE: Cardiovascular Risk Factors, Aging, and Incidence of Dementia.

aa DRS: Dementia Risk Score.

ab *ICD*: *International Classification of Diseases.*

ac OR: odds ratio.

ad HT: hormone therapy.

ae IXI: Information Extraction From Images.

af 3D-ViT: 3D vision transformer.

ag BAG: brain age gap.

ah GWAS: genome-wide association study.

ai SNP: single-nucleotide polymorphism.

aj xQTL: molecular quantitative trait locus.

ak MAE: mean absolute error.

al MAPT: microtubule-associated protein tau.

am TNFSF12: Tumor Necrosis Factor (Ligand) Superfamily, Member 12.

an VGG-16: Visual Geometry Group 16-Layer Network.

ao LAVA: Granular Neuron-Level Explainer.

ap XAI: explainable artificial intelligence.

aq IDP: imaging-derived phenotype.

ar FCN: fully convolutional network.

as pMCI: progressive mild cognitive impairment.

at sMCI: stable mild cognitive impairment.

au VBM: voxel-based morphometry.

av MWAN: multi-task weakly-supervised attention.

aw MMSE: Mini-Mental State Examination.

ax CDRSB: Clinical Dementia Rating–Sum of Boxes.

ay ADAS-Cog: Alzheimer Disease Assessment Scale–Cognitive Subscale.

az RMSE: root mean square error.

ba ADNI-GO/2: Alzheimer’s Disease Neuroimaging Initiative – Grand Opportunity / Phase 2.

bb CNN: convolutional neural network.

bc ADPC: Alzheimer Disease Prediction Challenge.

bd nADD: non-Alzheimer disease dementia.

be LEAR: learn-explain-reinforce.

bf RL: reinforcement learning.

bg PPM: Predictive Prognostic Model.

bh DA-MIDL: dual attention multi-instance deep learning.

bi GCN: graph convolutional network.

bj SHAP: Shapley Additive Explanations.

bk EMCI: early mild cognitive impairment.

bl DTI: diffusion tensor imaging.

bm ADC: apparent diffusion coefficient.

bn EHR: electronic health record.

bo ELM: extreme learning machine.

bp fMRI: functional magnetic resonance imaging.

bq DL: deep learning.

br DNN: deep neural network.

bs CSF: cerebrospinal fluid.

bt EEG: electroencephalography.

bu GBM: Gradient Boosting Machine.

bv T2-FLAIR: T2-weighted fluid-attenuated inversion recovery.

bw TADPOLE: The Alzheimer Disease Prediction of Longitudinal Evolution.

bx PREV-DEMALS: Predict to Prevent Frontotemporal Lobar Degeneration and Amyotrophic Lateral Sclerosis.

by FTD: frontotemporal dementia.

bz ALS: amyotrophic lateral sclerosis.

ca UCL: University College London.

cb NHI: National Health Insurance.

cc MAND: Multimodal Attention Network.

cd CTR: clinical trial registration.

ce SVM: support vector machine.

cf HC: healthy control.

cg VR: virtual reality.

ch WiGMM: Warped Infinite Gaussian Mixture.

ci FCRN: fully convolutional residual network.

cj MLP: multilayer perceptron.

ck LFP: local field potentials.

cl TUG: Timed Up and Go.

cm CVD: cardiovascular disease.

cn ViT: vision transformer.

co VCI: vascular cognitive impairment.

#### UK Biobank Dataset

UK Biobank enables population-level association studies and early-risk modeling. It has been widely used in AD diagnosis research, including the following notable studies:

Recent UK Biobank–based studies have applied diverse multimodal ML and deep learning approaches for AD risk prediction and diagnosis, integrating neuroimaging, genetic, clinical, and lifestyle data. These models generally achieved moderate to high performance (area under the curve [AUC] ≈0.77‐0.90) and demonstrated improved diagnostic utility compared with conventional assessment methods [79,81-83]. Several studies further emphasized the importance of genetic and hormonal factors in risk stratification [84,85]. In addition, explainable and semisupervised frameworks have enhanced model interpretability and scalability for population-level analysis, facilitating clinically relevant phenotyping and disease monitoring [86,87].

This section describes multimodal model implementation in the UK Biobank. As shown in the analysis and Table 2, UK Biobank data support AD diagnosis and risk prediction, but limitations remain: class imbalance, which may bias training, and the need for external validation to confirm generalizability beyond the UK Biobank cohort.

#### ADNI Dataset

ADNI provides a rich and diverse collection of demographic information, multimodal data, and clinical assessments. Owing to its comprehensive scope and longitudinal design, it has become one of the most widely adopted benchmark datasets for computer-aided diagnosis of AD. The following studies exemplify its use:

Recent ADNI-based studies have developed a wide range of multimodal and deep learning frameworks integrating neuroimaging, genetic, cognitive, and clinical data for AD diagnosis and MCI-to-AD progression prediction. Attention-based, multitask, ensemble, and time-to-event models have enabled accurate localization of disease-related regions, improved prognostic modeling, and enhanced interpretability through explainable artificial intelligence techniques such as SHAP (Shapley Additive Explanations) and counterfactual analysis [15,75,76,88-92,97]. Several approaches further incorporated RL, semisupervised learning, and data augmentation to improve robustness and generalizability in heterogeneous and imbalanced datasets [98-103,107]. These models typically achieved high diagnostic and prognostic performance (AUC up to ≈0.96), with some demonstrating strong external validation and clinical relevance [93-96,101,104-106,108]. Nevertheless, existing reviews and benchmarking studies have highlighted persistent limitations, including dataset bias, inconsistent evaluation protocols, and limited cross-center validation, underscoring the need for standardized and reproducible multimodal frameworks [119].

While ADNI provides a comprehensive and standardized multimodal resource for AD research and supports robust model performance, several limitations remain. These include class imbalance, underrepresentation of racially diverse populations, and limited external validation, which may bias model training and restrict generalizability across clinical settings.

#### Self-Collected Datasets

While public datasets such as ADNI provide standardized benchmarks, self-collected datasets enable more flexible acquisition of targeted modalities. Representative studies include the following.

Studies based on self-collected datasets have explored diverse multimodal fusion strategies. EEG- and local field potentials–based models, as well as hybrid MRI–PET–biomarker frameworks, demonstrated high diagnostic and staging accuracy and supported interpretable risk mapping [77,109,111,114,115]. In parallel, behavioral and digital biomarkers derived from handwriting, eye tracking, virtual reality, and motion capture have enabled noninvasive and low-cost screening with strong classification performance [22,78,110,112]. Large-scale real-world health records and hybrid deep learning models further facilitated population-level risk prediction and vascular cognitive impairment assessment, achieving robust AUC values above 0.90 [113,120]. Overall, self-collected datasets have expanded the scope of multimodal AD research by enabling flexible modality integration and novel biomarker discovery, while remaining constrained by limited sample sizes and heterogeneous acquisition protocols.

Self-collected datasets offer distinct advantages, including targeted modality acquisition, novel biomarker discovery, such as microRNA, local field potentials, and handwriting, and enhanced real-world clinical utility. However, self-collected datasets typically endure limited sample sizes, which increases susceptibility to overfitting and compromises generalizability across diverse populations.

### Multimodal Linguistic-Based Cognitive Impairment Datasets

Beyond multimodal clinical phenotyping datasets, multimodal linguistic-based cognitive impairment datasets represent an equally vital research resource. These datasets offer a noninvasive and cost-effective methodology for detecting cognitive decline, particularly valuable for identifying early-stage or subtle impairments where traditional neuroimaging or biomarker data may yield inconclusive results. Capturing spontaneous or semistructured speech and language patterns pushes the development of AI in speech data. Recent work is shown in Table 3.

**Table 3.** Multimodal linguistic-based cognitive impairment datasets related papers.

Study	Datasets	Model type	Type of task	Modalities	Outcomes	Validation	Results	Limitation
Ilias et al [121], 2023	ADReSS^a^ and ADReSSo^b^	Multimodal transformer (BERT^c^ and DeiT^d^) with optimal transport	Dementia detection (AD^e^ vs non-AD)	Audio (spectrograms) and text (transcripts)	Classification metrics and calibration	Internal: ADReSS or ADReSSo	Accuracy ≈91.25%, *F*_1_-score ≈91.06%; improved calibration vs baselines	Small, curated datasets, English-only, and potential overfitting
Poor et al [122], 2024	I-CONECT^f^	Multimodal cross-transformer with coattention	MCI^g^ prediction (MCI vs NC^h^)	Audio, text, and vision (facial video)	AUC^i^ scores	Internal: cross-validation	Trimodal AUC 85.3%, and outperformed unimodal (60.9%) and bimodal (76.3%) models	Single cohort (I-CONECT), cross-sectional, and complex architecture
Lin and Washington [123], 2024	DementiaBank (Pitt)	Wav2vec (audio) and Word2Vec (text)	Dementia classification	Audio, text, and timestamps	Accuracy and AUROC^j^	Internal: cross-validation	Text augmentation improved accuracy to ≈80% (AUROC 90%), and timestamps added minimal value	Single corpus: timestamps lacked resolution, and a modest sample size
Ortiz-Perez et al [124], 2023	DementiaBank (Pitt)	Multimodal ensemble (CNN^k^ and transformer)	Prediction of dementia signs	Audio and text	Classification accuracy	Internal: held-out test sets	Text-only transformer best (accuracy 90.36%) and audio contributed less than text	Single English dataset, broad diagnosis category, and task constrained to picture description
Ilias and Askounis [125], 2022	ADReSS (DementiaBank)	Transformer (BERT) and Siamese Network	AD identification and severity estimation	Text (transcripts)	Accuracy and interpretability (LIME^l^)	Internal: cross-validation	Single-task accuracy 87.50%, multitask accuracy 86.25%, and distinct linguistic patterns identified	Small dataset, text only, MMSE^m^ treated as categorical, and no acoustic information
Wen et al [126], 2023	DementiaBank (Pitt)	Transformer and causal counterfactual XAI^n^	AD detection	Text (part-of-speech tag features)	Accuracy; *F*_1_-score; feature importance	Internal: cross-validation	Accuracy 92.2%, *F*_1_-score 0.955, identified 12 key part-of-speech features linked to AD	Text only (part-of-speech), reliance on tagging accuracy, and no acoustic or imaging data
Chen et al [127], 2023	DementiaBank (Pitt)	SpeechFormer + + (hierarchical transformer)	Paralinguistic AD detection	Audio (acoustic features)	Accuracy; *F*_1_-score	Internal: held-out test sets	Outperformed standard transformers and CNN/RNN^o^ baselines and SOTA^p^ performance	Single corpus, complex computation, audio only, and no cross-lingual evaluation
Zheng et al [128], 2022	DementiaBank (Pitt)	N-gram, AWD-LSTM^q^, or neural models	Dementia detection	Text (context words, stop words, and part-of-speech)	Classification accuracy	Internal: held-out test data	Combined model (vocabulary and grammar) accuracy 81.54%, and grammar contributes comparably to context	Specific to task or language, and moderate performance vs multimodal approaches
Nambiar et al [129], 2022	DementiaBank (Pitt)	Deep Classifiers (BERT/ALBERT^r^ + BiLSTM^s^)	Early dementia detection	Text (transcripts)	Accuracy; *F*_1_-score	Internal: train and test splits	BERT + BiLSTM accuracy 0.812; ALBERT + BiLSTM *F*_1_-score 0.81; contextual embeddings superior	Text only; reliance on manual transcripts; single dataset
Priyadarshinee et al [130], 2023	ADReSSo-2021	ML^t^ classifiers (SVM^u^, RF^v^, and NN^w^)	AD detection	Audio and text (transcripts)	Classification accuracy	Internal: held-out test set	Text features (accuracy 88.7%) outperformed audio, and file-level features were superior to frame-level	Benchmarking context, single task, and single language
Liu et al [131], 2023	ADReSS, ADReSSo, and the local Chinese dataset	Ensemble ML (VAD^x^ pause and acoustic)	AD detection	Audio (acoustic and VAD pause features)	Accuracy	Internal: cross-validation; cross-lingual (Chinese)	Ensemble improved accuracy by ≈8% on ADReSS, and accuracy 80% on the local Chinese dataset	Small local dataset (n=10), handcrafted features, and ensemble complexity
Shah et al [23], 2023	ADReSS-M	Logistic regression and SVR	Cross-lingual AD detection; MMSE regression	Audio (duration, pause, and intelligibility) and metadata	Accuracy and RMSE^y^	External: Greek test set	English cross-validation accuracy 74.7%, Greek Test accuracy 69.57%, and MMSE RMSE 4.77 (Greek)	Small Greek sample, modest accuracy, and simple ML models vs deep learning
Mahajan and Baths [132], 2021	ADReSS	Bimodal framework (CNN-LSTM^z^ and Speech-GRU^aa^)	AD detection	Audio and text	Classification accuracy	Internal: cross-validation	Bimodal enriched model improved performance by ≈6.25% over acoustic baselines	Small dataset, potential overfitting, and single task (picture description)
Mei et al [133], 2023	ADReSS-M	Bilingual wav2vec 2.0 + XGBoost^ab^	Cross-lingual AD detection and MMSE prediction	Audio (acoustic, silence, and low-frequency bands)	Accuracy and RMSE	External: Greek test set	Accuracy 73.9% (Greek), MMSE RMSE 4.610, and low-frequency speech aided transfer	Very small Greek sample, speech-only, and challenge context
Meerza et al [134], 2022	ADReSS	FL^ac^ (LSTM^ak^ and feed-forward)	Privacy-preserving AD diagnosis	Audio (Mel-frequency and pause features)	Accuracy and fairness metrics	Internal: simulated FL clients	FL accuracy close to the centralized baseline, and q-FedAvg improved fairness	Simulated clients, single dataset, and relies on feature extraction
Chen et al [135], 2023	ADReSS-M	SVM or NN on pretrained features	Cross-lingual AD detection	Audio (paralinguistic and XLSR-53^ae^), and text (ASR^af^	Accuracy and RMSE	External: Greek test set	Accuracy 69.6% (Greek), RMSE 4.788, and paralinguistic features transferable	Performance below monolingual systems and reliance on ASR quality
Ilias et al [121], 2023	ADReSS	Multimodal transformer (ViT^ag^, BERT, and GMU^ah^)	AD detection	Audio (spectrograms) and text	Accuracy and *F*_1_-score	Internal: cross-validation	High eighties or low nineties accuracy, ViT is best for acoustic, and fusion surpassed SOTA	Small dataset, binary classification focus, and external generalization untested
Tamm et al [136], 2023	ADReSS-M^ai^	Sequence models (transfer learning)	Cross-lingual AD detection and MMSE	Audio features and demographics	Accuracy and RMSE	External: Greek test set	Accuracy 82.6% (Greek), RMSE 4.345, and ranked second in the challenge	Small Greek sample, acoustic only, and transfer limited to English-Greek
Woszczyk et al [137], 2022	ADReSS	Transformers vs traditional ML	AD detection	Audio and text	Classification accuracy	Internal: held-out test data	Data augmentation improved performance and was comparable to SOTA	Augmentations tuned for ADReSS and a single speech task
Jin et al [138], 2023	ADReSS-M	CONSEN^aj^ ensemble (acoustic and disfluency)	Multilingual AD detection and MMSE	Audio (acoustic embeddings and disfluency)	Accuracy and RMSE	External: Greek test set	First place in the challenge, accuracy 86.69% (Greek), and RMSE 3.727	Challenge dataset, ensemble complexity, and reliance on diarization quality

a ADReSS: Alzheimer Dementia Recognition Through Spontaneous Speech.

b ADReSSo: Alzheimer’s Dementia Recognition Through Spontaneous Speech only.

c BERT: Bidirectional Encoder Representations From Transformers.

d DeiT: Data-Efficient Image Transformers.

e AD: Alzheimer disease.

f I-CONECT: Identifying Cognition in the Elderly Through Conversational Engagement.

g MCI: mild cognitive impairment.

h NC: normal control.

i AUC: area under the curve.

j AUROC: area under the receiver operating characteristic curve.

k CNN: convolutional neural network.

l LIME: Local Interpretable Model-Agnostic Explanations.

m MMSE: Mini-Mental State Examination.

n XAI: explainable artificial intelligence.

o RNN: recurrent neural network.

p SOTA: state of the art.

q AWD-LSTM: Average stochastic gradient descent weight-dropped long short-term memory

r ALBERT: A Lite Bidirectional Encoder Representations From Transformers.

s BiLSTM: bidirectional long short-term memory.

t ML: machine learning.

u SVM: support vector machine.

v RF: random forest.

w NN: neural network.

x VAD: voice activity detection.

y RMSE: root mean square error.

z CNN-LSTM: convolutional neural network long short-term memory.

aa Speech-GRU: Speech Gated Recurrent Unit.

ab XGBoost: Extreme Gradient Boosting.

ac FL: federated learning.

ad LSTM: long short-term memory.

ae XLSR-53: cross-lingual speech representation-version 53

af ASR: automatic speech recognition.

ag ViT: vision transformer.

ah GMU: gated multimodal unit.

ai ADReSS-M: Alzheimer Dementia Recognition through Spontaneous Speech – Multimodal.

aj CONSEN: complementary and simultaneous ensemble.

Recent studies have shown that multimodal fusion of speech and text using transformer-based architectures substantially improves AD detection performance, with *F*_1_-scores above 0.90 on ADReSS and ADReSSo (Alzheimer’s Dementia Recognition Through Spontaneous Speech 2021 Challenge) datasets [121,132,139]. Linguistic feature engineering and interpretable language models further enhanced classification accuracy, achieving up to 92.2% accuracy and *F*_1_-scores of 0.955 using compact part-of-speech features [124-126,128,130]. Cross-lingual approaches based on language-agnostic and transfer learning methods enabled moderate generalization, with accuracies ranging from 69% to 73.9% in English-Greek transfer settings [23,127,133,136]. To support real-world deployment, lightweight and hierarchical models achieved around 80% accuracy with reduced computational cost [131,135]. In addition, data augmentation and ensemble strategies improved robustness in low-resource scenarios, yielding *F*_1_-score gains of 5%‐7% and competitive challenge performance (accuracy 86.69%) [123,137,138].

### Summarization Based on All Multimodal Datasets and Quantitative Analysis

Table 2 and Table 3 summarize the recent state-of-the-art models across the 2 major types of multimodal datasets, extracted according to the Cochrane Handbook. Full QUADAS-2 forms are available in Multimedia Appendix 5. Based on these results, the following quantitative synthesis compares performance trends across all multimodal datasets. Across the 4 major dataset categories, modality choices and model performance show clear dataset-dependent patterns as shown in Table 4. UK Biobank studies mainly combine MRI, clinical variables, and genetic features, with 2 diagnosis studies reporting an average accuracy of 71.4% (SD 5.2%) and 4 risk-prediction studies reaching an average AUC of 0.84 (SD 0.056). ADNI studies use the most comprehensive modality integrations, with 3 diagnosis studies averaging 92.5% (SD 3.8%) accuracy, 3 MCI-conversion studies achieving a mean AUC of 0.922 (SD 0.045), and risk-prediction studies reaching an average AUC of 0.81 (SD 0.06); these tasks collectively achieve the strongest results, with fusion models frequently reporting AUC values above 0.95. DementiaBank studies differ fundamentally by focusing on speech- and language-based modalities; 9 diagnosis studies report an average AUC of 0.813 (SD 0.042), and 5 cross-lingual AD-detection studies show a mean accuracy of 77% (SD 6.5%), where transformer architectures consistently outperform classical approaches, with models such as BERT + DeiT (Data-Efficient Image Transformers), BERT + ViT (vision transformer), and RoBERTa + (Robustly Optimized Bidirectional Encoder Representations From Transformers Approach) DNN (deep neural network) showing *F*_1_-scores exceeding 0.90. Self-collected datasets are typically smaller and more heterogeneous; 3 diagnosis studies report an average accuracy of 96% (SD 2.4%), and lightweight models such as EEGNet or ViT-based hybrids demonstrate strong predictive capacity when applied to EEG or structural MRI.

**Table 4.** Summary of representative modality combinations and top-performing models in multimodal AI^a^-aided AD^b^ diagnosis.

Dataset and task	Counts	Average performance	Best performance modalities	Related article
UK Biobank				
Diagnosis	2	Accuracy=71.4%	Retinal fundus images	[79,81-87,140]
Risk prediction	4	AUC^c^=84%	Clinical, biological assays, cognitive tests, and physical measures	[79,81-87,140]
Other	3	N/A^d^	Multimodal MRI^e^ (T1, T2, MRI, etc)	[79,81-87,140]
ADNI^f^				
Diagnosis	3	Accuracy=92.5%	Structural MRI features and neuropsychological tests	[15,75,76,79,80,89-92,94-108,119,141]
MCI^g^ conversion	3	AUC=92.2%	Structural MRI, clinical variables, and genetics (SNP^h^)	[15,75,76,79,80,89-92,94-108,119,141]
MMSE^i^ regression	2	No integration	Whole-brain T1-weighted MRI and clinical scores	[15,75,76,79,80,89-92,94-108,119,141]
Risk prediction	7	AUC=81%	MRI, PET^j^, clinical, and cognitive	[15,75,76,79,80,89-92,94-108,119,141]
Other	13	N/A	N/A	[15,75,76,79,80,89-92,94-108,119,141]
Dementia bank				
Diagnosis	9	AUC=81.3%	Text transcripts → part-of-speech feature vectors	Table 3
Cross-lingual AD detection	5	Accuracy=77%	Multimodal acoustic fusion	Table 3
Other	6	N/A	N/A	Table 3
Self-collected datasets				
Diagnosis	3	Accuracy=96%	MRI, PET, clinical, and genotype	[22,77,78,106,109-115,117,120,142]
Other	6	No integration	Different task	[22,77,78,106,109-115,117,120,142]

a AI: artificial intelligence.

b AD: Alzheimer disease.

c AUC: area under the curve.

d N/A: not available.

e MRI: magnetic resonance imaging.

f ADNI: Alzheimer Disease Neuroimaging Initiative.

g MCI: mild cognitive impairment.

h SNP: single-nucleotide polymorphism.

i MMSE: Mini-Mental State Examination.

j PET: positron emission tomography.

To interpret these results and limit metric inflation, note that purely internal cross-validation tends to overestimate performance: AUC is typically ≈5‐15 points higher than with external validation. Small or tightly controlled datasets also report accuracies ≈10%‐20% above those in large, heterogeneous cohorts. Severe class imbalance can further raise accuracy while lowering *F*_1_-score or sensitivity; without correction, imbalance may inflate results by ≈5%‐12%. Cross-sectional models often score higher in single-timepoint evaluations, whereas longitudinal designs usually yield lower but more stable estimates, which are more informative for follow-up and clinical use.

These findings should be interpreted in light of substantial heterogeneity and risk of bias. Variation in sample composition, task definitions, and evaluation procedures across datasets limits direct comparison of performance metrics. QUADAS-2 also indicated frequent unclear and high-risk in-patient selection, reference standards, and flow or timing, especially in studies using only internal validation or selected samples. Reported metrics, therefore, likely represent upper-bound estimates rather than expected real-world performance, and apparent gains often reflect dataset-specific effects rather than generalizable model superiority.

Overall, the evidence shows that modality effectiveness varies substantially across datasets, transformer models deliver the highest gains in speech-language tasks, and large clinical phenotyping datasets such as UK Biobank and ADNI still rely mainly on traditional machine-learning or custom fusion frameworks rather than modern cross-modal transformers. This gap highlights an opportunity to develop transformer-based multimodal integration approaches tailored to large, heterogeneous clinical datasets.

### Multimodal Fusion Taxonomy

A structured multimodal fusion taxonomy clarifies the performance of different integration strategies across datasets (Tables 2 and 3). A total of 4 main paradigms are commonly used: early, intermediate, late, and attention- or graph-based fusion.

Early fusion concatenates low-level features and performs well for aligned modalities such as MRI + PET, often achieving AUC>0.95 in ADNI studies, but is sensitive to missing data and feature-scale heterogeneity. Intermediate fusion combines latent representations from modality-specific encoders and is effective for heterogeneous inputs such as MRI + speech or EEG + clinical data, as demonstrated by high performance in ADReSS-based models, although it may be unstable in small datasets. Late fusion aggregates model outputs and is robust to missing modalities, performing well in large datasets such as the UK Biobank, but underuses fine-grained cross-modal interactions.

Across paradigms, limited modality availability and high acquisition costs remain key challenges, underscoring the need for adaptive and clinically feasible fusion strategies.

## Discussion

### Principal Findings

This review synthesized multimodal AI studies for AD across diverse dataset families, including clinical phenotyping and cognitive-linguistic datasets. Multimodal fusion generally outperformed unimodal baselines, but the gain is dataset-dependent and should be interpreted cautiously. Strong performance in curated cohorts and constrained speech benchmarks may not generalize to population-based or multicenter settings. QUADAS-2 also indicated frequent risk of bias and unclear reporting across domains, likely inflating metrics and limiting comparability. Accordingly, headline accuracy and AUC should be treated as upper-bound estimates unless supported by external validation and transparent reporting.

### Challenges and Future Directions

In recent years, multimodal models have demonstrated remarkable potential in computer-aided diagnosis and risk prediction for AD. While these methods have achieved significant successes, several challenges remain that warrant careful examination. In this section, this systematic review summarizes the common limitations identified in existing studies and proposes directions for future research to advance the field.

### Clinical and Translational Implications

Multimodal AI could support AD diagnosis through several clinical pathways. In memory clinics, models combining MRI, cognitive scores, and blood biomarkers could triage referrals, prioritizing patients for specialist review or PET. In general practice, speech-based and routine clinical-feature models could be embedded in consultations to flag early cognitive change. In radiology, MRI-clinical fusion could act as a second reader, reducing interobserver variability and supporting less experienced clinicians. Where imaging or specialist access is limited, speech, digital questionnaires, and basic clinical data could enable telemedicine-based screening and follow-up. At the population level, these models could support risk stratification and targeted monitoring. To enable real-world deployment, research should prioritize external multicenter validation, integration with electronic health records, and evaluation of regulatory feasibility, cost-effectiveness, and clinical impact.

### Ethical and Regulatory Implications

Deploying multimodal AI for AD diagnosis requires ethical and regulatory safeguards. As datasets often combine imaging, clinical records, genomics, and speech, they fall under strict privacy regimes (eg, General Data Protection Regulation in the European Union; HIPAA [Health Insurance Portability and Accountability Act] in the United States), requiring explicit consent, data minimization, and secure handling, with added complexity for sensitive modalities such as speech and genomic data. Clinical deployment is also shaped by medical-AI governance frameworks (eg, the European Union AI Act, Food and Drug Administration Software as a Medical Device guidance, and UK Medicines and Healthcare products Regulatory Agency Good Machine Learning Practice), which emphasize transparency, risk management, and postdeployment monitoring. Fairness is essential because demographic imbalance can yield uneven performance across age, ethnicity, and language groups. Interpretability (eg, imaging attention maps and linguistic saliency) supports clinical accountability and aligns with explainability expectations. Future work should incorporate privacy-preserving methods, bias audits, and regulatory-aligned validation pipelines to enable responsible clinical integration.

### Data Privacy and Data-Sharing Constraints

Access to multimodal AD data remains severely restricted by privacy regulations and ethical constraints, which limit data sharing and external validation. This restricts the sharing and usage of comprehensive datasets needed for robust external validation and generalizability.

Federated learning (FL) provides a technically viable privacy-preserving solution; however, differences in data formats and institutional infrastructures still impede its large-scale deployment. For instance, Meerza et al [134] pioneered FL for AD speech diagnosis using mel-frequency cepstral coefficients and pause features, maintaining model performance while ensuring privacy through q-FedAvg/q-FedSGD optimization. Nambiar [129] validated an ALBERT (A Lite Bidirectional Encoder Representations From Transformers) + BiLSTM (bidirectional long short-term memory) hybrid model on the ADReSS dataset, achieving strong performance without compromising data confidentiality. In parallel, multi-institutional collaborations leveraging publicly available datasets such as ADNI, UK Biobank, and OASIS have enabled richer external validation while adhering to rigorous privacy standards [15,79,88,95,100,139,140].

Despite encouraging results, FL still lacks harmonized protocols and interoperable platforms. This limits cross-center reproducibility and weakens clinical credibility. International collaboration also remains constrained by regulatory differences. Future work should prioritize unified federated frameworks with standardized protocols and privacy-preserving methods to enable secure global data collaboration [143,144].

As most datasets lack fully matched modalities per participant, multimodal fusion often relies on representation- or population-level integration rather than early fusion. Early fusion requires paired samples and is therefore infeasible across datasets. By contrast, late fusion and embedding-level integration can train unimodal models separately and combine them via meta-learners, cross-modal transformers, or probabilistic ensembles. Domain adaptation, transfer learning, and harmonization can also combine heterogeneous cohorts at the population level to improve generalizability. A standardized benchmark could further support this by defining shared preprocessing, label taxonomies, and evaluation metrics, enabling meaningful comparison or representation-stage fusion even without subject-level pairing.

### Data Imbalance

Severe class imbalance remains a major obstacle, biasing training toward the majority class and inflating accuracy while masking low sensitivity to early disease. In addition, datasets such as the UK Biobank are dominated by White European ancestry, limiting generalizability across racially and ethnically diverse populations. Addressing this requires both technical mitigation and proactive recruitment of underrepresented groups so models better reflect population heterogeneity.

Researchers have applied data-level interventions such as generative adversarial network–based augmentation, diffusion models, and resampling [123,137,138,145,146]; algorithm-level solutions, including cost-sensitive, loss-focused, ensemble, and class-weighted training schemes [147-152]; and evaluation-focused remedies [153] have been developed to mitigate biases.

Current methods frequently introduce new challenges, such as overfitting or inadequate performance in minority classes. Moreover, efforts to increase diversity remain inadequate. Future directions should focus on novel adaptive resampling methods, generative methods for synthetic minority data creation, and dedicated efforts to include and characterize underrepresented populations to ensure equitable and robust clinical applicability across diverse populations.

### Lack of Standardized and Longitudinal Data

Differences in acquisition protocols and diagnostic criteria across datasets limit comparability of imaging, cognitive, and biomarker outcomes. Longitudinal evidence is also constrained: even in relatively standardized resources such as ADNI, limited long-term follow-up hampers modeling the temporal dynamics of disease progression.

Future work should standardize key acquisition elements and diagnostic criteria across longitudinal studies and strengthen coordination across institutions. Building on this, a multimodal benchmark spanning imaging, clinical, biomarker, behavioral, and linguistic modalities would enable cross-dataset validation, improve comparability, and support reproducible evaluation of new models. These steps would strengthen temporal modeling and provide more reliable evidence for clinical translation.

### Dataset-Specific Limitations

Data imbalance is prevalent across many AD datasets, but the nature of this issue varies substantially between cohorts. This review, therefore, outlines the dataset-specific limitations of commonly used AD cohorts and corpora.

ADNI participants are generally healthier, with fewer comorbidities and a restricted age range (55‐90 y), limiting representativeness. Protocol differences across centers and evolving diagnostic standards introduce heterogeneity, while frequent reliance on subsets hampers comparability [154].

Of UK Biobank, dementia outcomes are derived mainly from health records, leading to potential misclassification and delayed ascertainment. Participants show strong volunteer bias, and PET or cerebrospinal fluid biomarkers are limited to a small subset, constraining multimodal analyses [155].

OASIS provides open neuroimaging data but with relatively small AD/MCI sample sizes and inconsistent modality coverage. Limited longitudinal depth and cross-scanner variability further reduce reproducibility [156].

Of NACC, data are aggregated from multiple centers with heterogeneous recruitment and diagnostic protocols, making harmonization challenging. The cohort is clinic-based rather than population-representative, and missing biomarker modalities are common [157].

Although high-quality, Australian Imaging, Biomarkers and Lifestyle Study is smaller than ADNI and NACC and is often used only for validation. Regional recruitment and protocol differences reduce ethnic diversity and cross-cohort comparability [158].

Of Pitt Corpus, this is the most widely used speech dataset, but remains small and imbalanced. Tasks are constrained, limiting ecological validity, and cross-linguistic generalizability is poor [159].

Of the ADReSS series, the ADReSS benchmarks provide standardized speech corpora but are modest in size and restricted to English. Narrow task design and small training partitions raise concerns of overfitting and limited external validity [18].

Of self-collected cohorts, locally collected datasets often involve small, single-site samples with heterogeneous acquisition protocols. Missing modalities, limited follow-up, and selection bias further restrict their generalizability [153].

Dataset challenges are compounded by unrepresentative cohorts, incomplete modalities, and poor cross-center consistency, limiting model robustness and cross-dataset generalization in AD diagnosis. Future work should improve data coordination and standardization, enable more practical sharing mechanisms, and adopt cross-cohort validation where feasible. Strengthening data quality and access is essential for translating multimodal AI methods into clinical use.

### Model Interpretability and Explainability

A major limitation of multimodal ML models in clinical AD diagnosis is limited interpretability and transparency. Many high-performing models provide insufficient insight into their decision processes, which can hinder clinical adoption and reduce confidence among end users.

Efforts that have been made toward model interpretability include designing inherently transparent models. For example, some studies demonstrate emerging explainability strategies, including hybrid neuro-symbolic models [160] that generate interpretable reports and post hoc methods such as SHAP, LIME (Local Interpretable Model-Agnostic Explanations), gradient-based saliency, and graph-masking techniques [161,162], which collectively enhance transparency in multimodal AD diagnosis.

Current interpretability methods often fail to produce explanations that clinicians can use reliably. Future work should prioritize clinically grounded explainability, including interactive visualizations and concise workflow-aligned natural-language summaries. Hybrid designs that combine deep learning with structured reasoning can further improve transparency by making decision logic explicit. For deployment, models should also report prediction uncertainty and demonstrate compatibility with clinical systems and regulatory requirements.

Beyond technical advances, incorporating patient and public involvement can improve multimodal AI development for AD. Patients and caregivers can help shape evaluation and result communication, not just act as end users, aligning explanations with patient priorities and addressing transparency and fairness. Engaging patient and public involvement earlier in model design may therefore support more interpretable and clinically usable diagnostic tools.

### Heterogeneous Multiview Learning Problem

Integrating data across studies is challenging because single datasets rarely cover all modalities, forcing combinations such as ADNI with UK Biobank. However, differences in cohorts, imaging protocols, and cognitive assessment frameworks create substantial heterogeneity that limits direct pooling and comparability.

This heterogeneity hinders building unified models that generalize across nonoverlapping cohorts, so single-dataset models often fail out of domain. Platform-agnostic methods that tolerate missing or inconsistent modalities are therefore needed. Proposed solutions include shared latent-space learning [163], multibranch networks [164], and mixture-of-experts architectures [165] to support partial fusion and cross-dataset adaptation, but most still assume strong cross-domain alignment or require substantial retraining under domain shift.

Despite recent progress, multimodal methods often assume strict cross-domain alignment and require extensive retraining under domain shift or missing modalities. Future work should develop robust, platform-agnostic frameworks that adapt to changing modality availability and distribution shifts with minimal performance loss and advance representation learning to derive stable joint embeddings from heterogeneous data.

### Uncertainty Quantification and Clinical Applicability

Although multimodal AD models have advanced, most studies still omit uncertainty quantification (eg, confidence or prediction intervals). Models typically provide deterministic outputs without communicating reliability, despite clinicians relying on uncertainty to guide management and treatment decisions. Future work should embed uncertainty metrics into diagnostic models to better align with clinical needs and improve interpretability, reliability, and real-world adoption.

### Risks of Data Leakage in Multimodal AI Modeling

Another limitation is data leakage, which can inflate performance. Common forms include subject-level leakage (samples from the same participant in both training and test sets), patch-level overlap in MRI slice and patch models, and transcript or utterance-level leakage in speech datasets when multiple segments come from 1 individual. Many studies did not report whether participant-independent splits were enforced. Clearer reporting of partitioning and rigorous participant-level cross-validation are therefore essential to ensure real-world generalizability.

### Conclusions

This review synthesizes evidence on multimodal AI approaches for AD across clinical, neuroimaging, genetic, and linguistic data, systematically comparing modeling strategies, validation practices, and performance trends across heterogeneous datasets. In contrast to prior modality-specific reviews, the findings show that multimodal models generally outperform unimodal approaches, although performance varies substantially with dataset characteristics, modality availability, and cross-source alignment. High accuracies are often reported in curated or internally validated cohorts, whereas population-based and externally validated studies yield more modest but clinically realistic results, reflecting substantial heterogeneity and risk of bias.

Despite these limitations, the evidence demonstrates that multimodal AI captures complementary biological and behavioral signals relevant to AD, offering clear advantages for diagnosis and risk prediction. Transformer-based architectures and speech- or behavior-derived modalities show promise for scalable and noninvasive early detection. However, meaningful clinical translation will require harmonized benchmarking, transparent reporting, and rigorous external validation. Overall, this review advances the field by contextualizing performance gains within their methodological constraints and by outlining practical directions for developing robust, interpretable, and generalizable multimodal AI systems. These insights support the responsible integration of AI into real-world dementia screening, risk prediction, and early intervention strategies.

## Supplementary material

10.2196/85414 Multimedia Appendix 1 Full database search strategies for PubMed, Scopus, IEEE Xplore, and ACM Digital Library, including complete Boolean queries, search fields, filters, and publication date limits used for study identification.

10.2196/85414 Multimedia Appendix 2 Performance evaluation for AD diagnosis. AD: Alzheimer disease.

10.2196/85414 Multimedia Appendix 3 Complete QUADAS-2 risk-of-bias assessments for all included studies, summarizing judgments across patient selection, index test, reference standard, and flow or timing, with detailed study-level ratings. QUADAS-2: Revised Quality Assessment of Diagnostic Accuracy Studies Tool.

10.2196/85414 Multimedia Appendix 4 Overview of traditional machine-learning models applied in Alzheimer disease research, including SVM, decision trees, HMMs, KNN, logistic regression, GMMs, and foundational CNN or RL descriptions, with methodological principles and limitations. CNN: convolutional neural network; GMM: gaussian mixture models; HMM: hidden markov model ; KNN: k-nearest neighbors; RL: reinforcement learning; SVM: support vector machine.

10.2196/85414 Multimedia Appendix 5 Cochrane Handbook 5.3.3–aligned data-extraction tables summarizing study design, datasets, participants, modalities, preprocessing, model architectures, validation schemes, outcomes, and limitations for all included studies.

10.2196/85414 Checklist 1 Completed PRISMA 2020, PRISMA-S checklist, and PRISMA expanded checklist specifying reporting locations for all required items, including eligibility criteria, search methods, extraction procedures, bias assessments, and synthesis reporting. PRISMA: Preferred Reporting Items for Systematic Reviews and Meta-Analyses; PRISMA-S: Preferred Reporting Items for Systematic Reviews and Meta-Analyses extension for literature searches.

## Abbreviations

AD: Alzheimer disease
AdaBoost: Adaptive Boosting
ADNI: Alzheimer’s Disease Neuroimaging Initiative
ADReSS: Alzheimer’s Dementia Recognition Through Spontaneous Speech
ADReSSo: Alzheimer’s Dementia Recognition Through Spontaneous Speech 2021 Challenge
AI: artificial intelligence
ALBERT: A Lite Bidirectional Encoder Representations From Transformers
AUC: area under the curve
BERT: Bidirectional Encoder Representations From Transformers
BiLSTM: bidirectional long short-term memory
DeiT: Data-Efficient Image Transformers
DNN: DNN
EEG: electroencephalography
FL: federated learning
HIPAA: Health Insurance Portability and Accountability Act
LightGBM: Light Gradient-Boosting Machine
LIME: Local Interpretable Model-Agnostic Explanations
MCI: mild cognitive impairment
ML: machine learning
MRI: magnetic resonance imaging
NACC: National Alzheimer’s Coordinating Centre
OASIS: Open Access Series of Imaging Studies
PET: positron emission tomography
PRISMA: Preferred Reporting Items for Systematic Reviews and Meta-Analyses
PRISMA-S: Preferred Reporting Items for Systematic Reviews and Meta-Analyses literature search extension
QUADAS-2: Revised Quality Assessment of Diagnostic Accuracy Studies tool
RL: reinforcement learning
RoBERTa+: Robustly Optimized Bidirectional Encoder Representations From Transformers Approach
SHAP: Shapley Additive Explanations
ViT: vision transformer
XGBoost: Extreme Gradient Boosting
