# Supplementary material for: Multimodal AI for Alzheimer Disease Diagnosis: Systematic Review of Datasets, Models, and Modalities
Source: J Med Internet Res. 2026 Mar 25;28:e85414. doi: 10.2196/85414 (PMC13018777; doi:10.2196/85414)

**Search Terms in each database**

1. **PubMed Search (447)**

**Search key words:** (("Alzheimer Disease"[Mesh] OR Alzheimer*[tiab] OR dementia[tiab] OR "mild cognitive impairment"[tiab] OR MCI[tiab]) AND (diagnos*[tiab] OR detect*[tiab] OR classif*[tiab] OR "risk prediction"[tiab] OR screening[tiab] OR prognosis[tiab] OR "disease progression"[tiab]) AND ("artificial intelligence"[tiab] OR "machine learning"[tiab] OR "deep learning"[tiab] OR "neural network*"[tiab] OR transformer*[tiab] OR "large language model*"[tiab] OR "self-supervised"[tiab] OR "reinforcement learning"[tiab] OR "ensemble learning"[tiab]) AND (MRI[tiab] OR fMRI[tiab] OR PET[tiab] OR EEG[tiab] OR "magnetic resonance"[tiab] OR "neuroimaging"[tiab] OR biomarker*[tiab] OR speech[tiab] OR voice[tiab] OR language[tiab] OR transcript*[tiab] OR genetics[tiab] OR "electronic health record*"[tiab] OR wearable*[tiab] OR sensor*[tiab] OR "clinical data"[tiab]) AND ("multimodal"[tiab] OR "multisource"[tiab] OR "data fusion"[tiab] OR "cross-modal"[tiab])) AND ("2019/01/01"[Date - Publication] : "2025/11/01"[Date - Publication])

| Year | count |
| --- | --- |
| 2019 | 28 |
| 2020 | 21 |
| 2021 | 46 |
| 2022 | 51 |
| 2023 | 69 |
| 2024 | 106 |
| 2025 | 151 |

**Link:** https://pubmed.ncbi.nlm.nih.gov/?term=%28%28%22Alzheimer+Disease%22%5BMesh%5D+OR+Alzheimer*%5Btiab%5D+OR+dementia%5Btiab%5D+OR+%22mild+cognitive+impairment%22%5Btiab%5D+OR+MCI%5Btiab%5D%29+AND+%28diagnos*%5Btiab%5D+OR+detect*%5Btiab%5D+OR+classif*%5Btiab%5D+OR+%22risk+prediction%22%5Btiab%5D+OR+screening%5Btiab%5D+OR+prognosis%5Btiab%5D+OR+%22disease+progression%22%5Btiab%5D%29+AND+%28%22artificial+intelligence%22%5Btiab%5D+OR+%22machine+learning%22%5Btiab%5D+OR+%22deep+learning%22%5Btiab%5D+OR+%22neural+network*%22%5Btiab%5D+OR+transformer*%5Btiab%5D+OR+%22large+language+model*%22%5Btiab%5D+OR+%22self-supervised%22%5Btiab%5D+OR+%22reinforcement+learning%22%5Btiab%5D+OR+%22ensemble+learning%22%5Btiab%5D%29+AND+%28MRI%5Btiab%5D+OR+fMRI%5Btiab%5D+OR+PET%5Btiab%5D+OR+EEG%5Btiab%5D+OR+%22magnetic+resonance%22%5Btiab%5D+OR+%22neuroimaging%22%5Btiab%5D+OR+biomarker*%5Btiab%5D+OR+speech%5Btiab%5D+OR+voice%5Btiab%5D+OR+language%5Btiab%5D+OR+transcript*%5Btiab%5D+OR+genetics%5Btiab%5D+OR+%22electronic+health+record*%22%5Btiab%5D+OR+wearable*%5Btiab%5D+OR+sensor*%5Btiab%5D+OR+%22clinical+data%22%5Btiab%5D%29+AND+%28%22multimodal%22%5Btiab%5D+OR+%22multisource%22%5Btiab%5D+OR+%22data+fusion%22%5Btiab%5D+OR+%22cross-modal%22%5Btiab%5D%29%29+AND+%28%222019%2F01%2F01%22%5BDate+-+Publication%5D+%3A+%222025%2F11%2F01%22%5BDate+-+Publication%5D%29&filter=years.2019-2025

1. **Scopus (1086)**

**Search key words:**

TITLE-ABS-KEY ( ( "Alzheimer*"  OR  "Alzheimer disease"  OR  dementia  OR  "mild cognitive impairment"  OR  mci )  AND  ( diagnos*  OR  detect*  OR  classif*  OR  "risk prediction"  OR  screening  OR  prognosis  OR  "disease progression" )  AND  ( "artificial intelligence"  OR  "machine learning"  OR  "deep learning"  OR  "neural network*"  OR  transformer*  OR  "large language model*"  OR  "self-supervised"  OR  "reinforcement learning"  OR  "ensemble learning" )  AND  ( mri  OR  fmri  OR  pet  OR  eeg  OR  "magnetic resonance"  OR  neuroimaging  OR  biomarker*  OR  speech  OR  voice  OR  language  OR  transcript*  OR  genetics  OR  "electronic health record*"  OR  wearable*  OR  sensor*  OR  "clinical data" )  AND  ( "multimodal"  OR  "multisource"  OR  "data fusion"  OR  "cross-modal" ) )  AND  PUBYEAR  >  2018

LINK:

<https://www.scopus.com/results/results.uri?sort=plf-f&src=s&sid=92f60c0ae323fc53acbc19d2b6dc3a07&sot=a&sdt=cl&sl=722&s=TITLE-ABS-KEY%28%28%22Alzheimer*%22+OR+%22Alzheimer+disease%22+OR+dementia+OR+%22mild+cognitive+impairment%22+OR+MCI%29AND%28diagnos*+OR+detect*+OR+classif*+OR+%22risk+prediction%22+OR+screening+OR+prognosis+OR+%22disease+progression%22%29AND%28%22artificial+intelligence%22+OR+%22machine+learning%22+OR+%22deep+learning%22+OR+%22neural+network*%22+OR+transformer*+OR+%22large+language+model*%22+OR+%22self-supervised%22+OR+%22reinforcement+learning%22+OR+%22ensemble+learning%22%29AND%28MRI+OR+fMRI+OR+PET+OR+EEG+OR+%22magnetic+resonance%22+OR+neuroimaging+OR+biomarker*+OR+speech+OR+voice+OR+language+OR+transcript*+OR+genetics+OR+%22electronic+health+record*%22+OR+wearable*+OR+sensor*+OR+%22clinical+data%22%29AND%28%22multimodal%22+OR+%22multisource%22+OR+%22data+fusion%22+OR+%22cross-modal%22%29%29AND+PUBYEAR+%26gt%3B+2018&origin=resultslist&editSaveSearch=&txGid=105284fd8bd7dc983f3ec209507047db&sessionSearchId=92f60c0ae323fc53acbc19d2b6dc3a07&limit=10&yearFrom=2019&yearTo=2025>

| Year | count |
| --- | --- |
| 2019 | 51 |
| 2020 | 61 |
| 2021 | 87 |
| 2022 | 120 |
| 2023 | 159 |
| 2024 | 249 |
| 2025 | 359 |

1. **IEEE Xplore (2229)**

**Search command:** (("Alzheimer*" OR "Alzheimer disease" OR dementia OR "mild cognitive impairment" OR MCI) AND (diagnosis* OR detect* OR classify* OR "risk prediction" OR screening) AND ("artificial intelligence" OR "machine learning" OR "deep learning" OR transformer* OR "large language model*" OR "self-supervised" OR "reinforcement learning") AND (MRI OR PET OR EEG OR "neuroimaging" OR biomarker* OR speech OR voice OR language OR "multimodal" OR "data fusion"))

**Filters applied:** 2020-2025

**LINK:**

<https://ieeexplore.ieee.org/search/searchresult.jsp?action=search&matchBoolean=true&queryText=(((%22Alzheimer*%22%20OR%20%22Alzheimer%20disease%22%20OR%20dementia%20OR%20%22mild%20cognitive%20impairment%22%20OR%20MCI)%20AND%20(diagnosis*%20OR%20detect*%20OR%20classify*%20OR%20%22risk%20prediction%22%20OR%20screening)%20AND%20(%22artificial%20intelligence%22%20OR%20%22machine%20learning%22%20OR%20%22deep%20learning%22%20OR%20transformer*%20OR%20%22large%20language%20model*%22%20OR%20%22self-supervised%22%20OR%20%22reinforcement%20learning%22)%20AND%20(MRI%20OR%20PET%20OR%20EEG%20OR%20%22neuroimaging%22%20OR%20biomarker*%20OR%20speech%20OR%20voice%20OR%20language%20OR%20%22multimodal%22%20OR%20%22data%20fusion%22)))&highlight=true&returnType=SEARCH&matchPubs=true&returnFacets=ALL&ranges=2020_2025_Year>

| Year | count |
| --- | --- |
| 2020 | 115 |
| 2021 | 177 |
| 2022 | 261 |
| 2023 | 496 |
| 2024 | 677 |
| 2025 | 503 |

1. **ACM Digital Library (2067)**

**Search command:** [[All: "alzheimer*"] OR [All: "Alzheimer disease"] OR [All: dementia] OR [All: "mild cognitive impairment"] OR [All: mci]] AND [[All: diagnosis*] OR [All: detect*] OR [All: classify*] OR [All: "risk prediction"] OR [All: screening]] AND [[All: "machine learning"] OR [All: "deep learning"] OR [All: "artificial intelligence"] OR [All: transformer*] OR [All: "large language model*"] OR [All: "self-supervised"] OR [All: "reinforcement learning"]] AND [[All: speech] OR [All: voice] OR [All: language] OR [All: mri] OR [All: pet] OR [All: eeg] OR [All: "multimodal"] OR [All: "data fusion"] OR [All: "cross-modal"]] AND [E-publication Date: (01/01/2020 TO 12/31/2025)]]

**LINK:** <https://dl.acm.org/action/doSearch?fillQuickSearch=false&expand=dl&AllField=%28%22Alzheimer*%22+OR+%22Alzheimer+disease%22+OR+dementia+OR+%22mild+cognitive+impairment%22+OR+MCI%29+AND+%28diagnosis*+OR+detect*+OR+classify*+OR+%22risk+prediction%22+OR+screening%29+AND+%28%22machine+learning%22+OR+%22deep+learning%22+OR+%22artificial+intelligence%22+OR+transformer*+OR+%22large+language+model*%22+OR+%22self-supervised%22+OR+%22reinforcement+learning%22%29+AND+%28speech+OR+voice+OR+language+OR+MRI+OR+PET+OR+EEG+OR+%22multimodal%22+OR+%22data+fusion%22+OR+%22cross-modal%22%29&AfterYear=2020&BeforeYear=2025&queryID=30%2F9957794450&startPage=0&content=standard&target=default&sortBy=>

| Year | count |
| --- | --- |
| 2020 | 200 |
| 2021 | 244 |
| 2022 | 273 |
| 2023 | 352 |
| 2024 | 533 |
| 2025 | 465 |

1. **Cochrane Library (515)**

**Search manager:**

#1 Alzheimer Disease[MeSH] OR Dementia [MeSH] OR MCI[MeSH]

#2 (machine learning OR deep learning OR artificial intelligence OR transformer* OR large language model* OR self supervised OR multimodal OR data fusion)

#3 (diagnos* OR screening OR early detection OR risk prediction)

#4 #1 AND #2 AND #3

LINK:

<https://www.cochranelibrary.com/advanced-search/search-manager?p_p_id=58_INSTANCE_MODAL&p_p_lifecycle=0&p_p_state=normal&saveLastPath=false&_58_INSTANCE_MODAL_redirect=%2Fadvanced-search%2Fsearch-manager>

1. **arXiv (1081)**

**Search command:** (Alzheimer OR dementia OR "mild cognitive impairment" OR MCI) AND ("machine learning" OR "deep learning" OR transformer OR "large language model" OR "self-supervised" OR "multimodal" OR "data fusion")

**Subject:** Computer Science(cs), Electrical Engineering and Systems Science (eess), Quantitative Biology(q-bio), Mathematics(math), Statistcs(stat)

**Date range <** 2025-12-31

**LINK:**

<https://arxiv.org/search/advanced?advanced=&terms-0-operator=AND&terms-0-term=Alzheimer+OR+dementia+OR+%22mild+cognitive+impairment%22+OR+MCI&terms-0-field=all&terms-1-operator=AND&terms-1-term=%22machine+learning%22+OR+%22deep+learning%22+OR+transformer+OR+%22large+language+model%22+OR+%22self-supervised%22+OR+%22multimodal%22+OR+%22data+fusion%22&terms-1-field=title&classification-computer_science=y&classification-eess=y&classification-mathematics=y&classification-physics_archives=all&classification-q_biology=y&classification-statistics=y&classification-include_cross_list=include&date-year=&date-filter_by=date_range&date-from_date=2020-01-01&date-to_date=2025-12-31&date-date_type=submitted_date&abstracts=show&size=50&order=-announced_date_first>

arXive_downloade_demo:


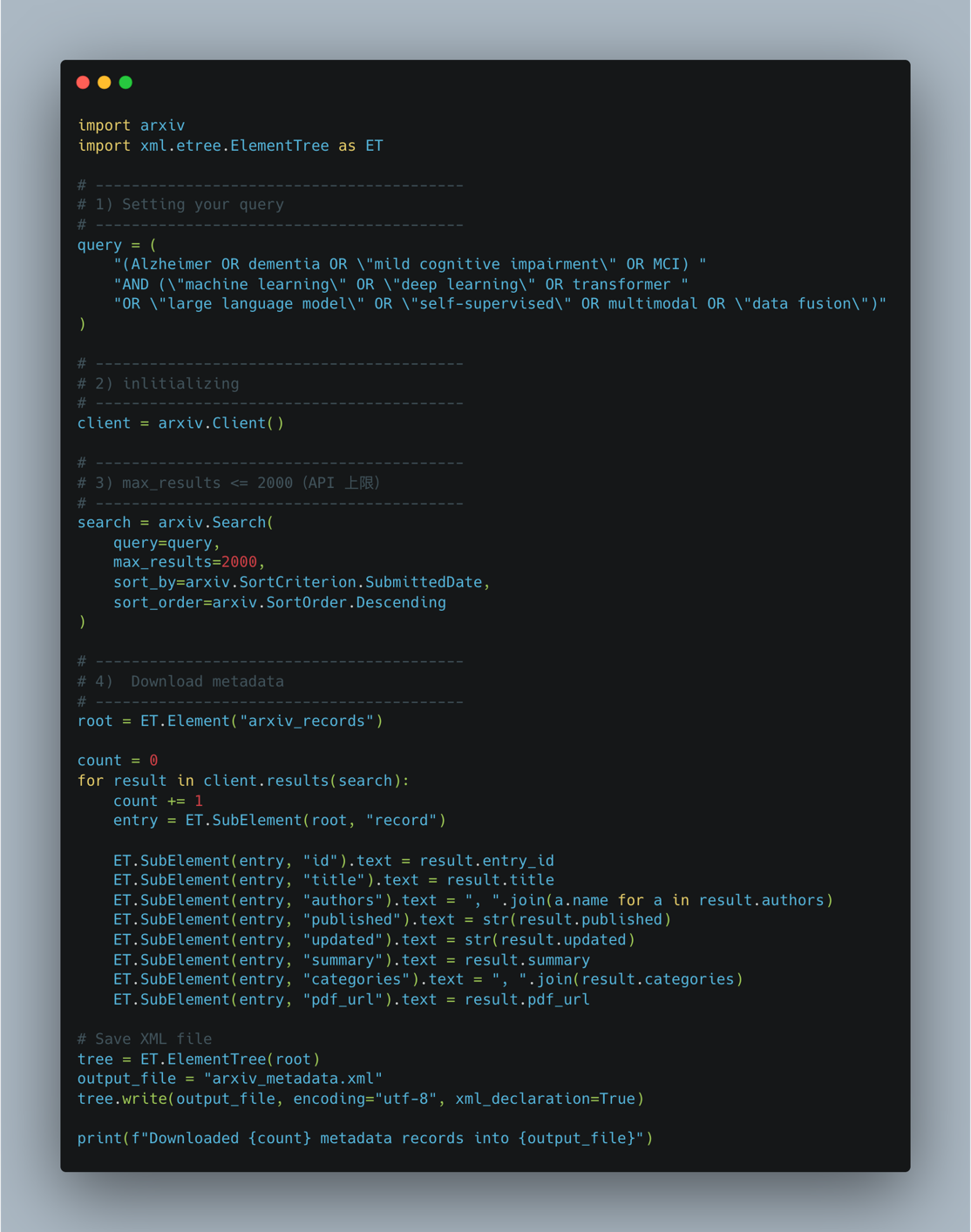


XML2Bib_demo:


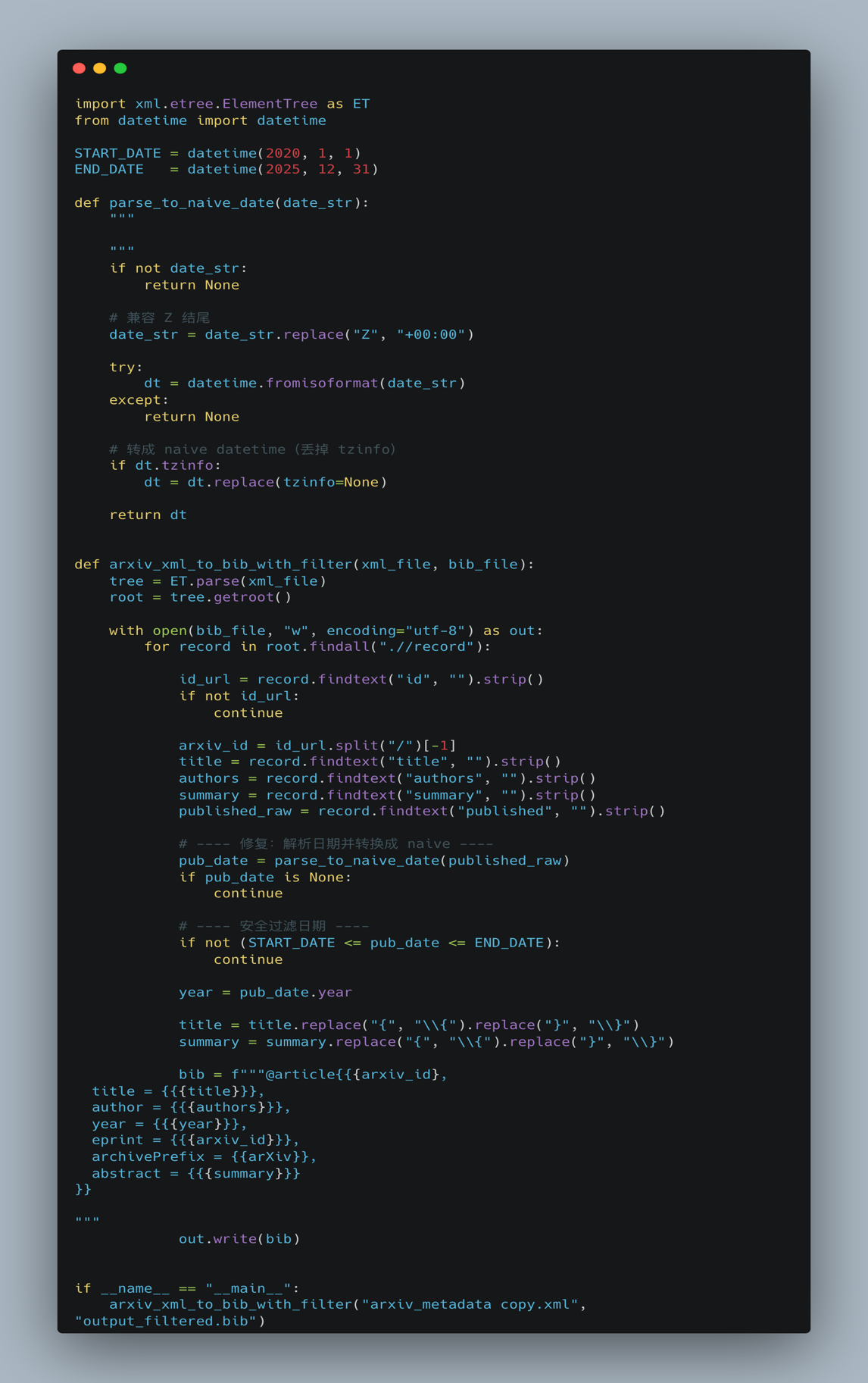

Supplement: Multimedia Appendix 1 — Full database search strategies for PubMed, Scopus, IEEE Xplore, and ACM Digital Library, including complete Boolean queries, search fields, filters, and publication date limits used for study identification. [file jmir-v28-e85414-s001.docx]
