# Supplementary material for: Multimodal AI for Alzheimer Disease Diagnosis: Systematic Review of Datasets, Models, and Modalities
Source: J Med Internet Res. 2026 Mar 25;28:e85414. doi: 10.2196/85414 (PMC13018777; doi:10.2196/85414)
Supplement: Multimedia Appendix 2 — Performance evaluation for AD diagnosis. AD: Alzheimer disease. [file jmir-v28-e85414-s002.docx]

## Performance Evaluation Metrics

These evaluation metrics not only guide model selection and optimisation but also provide a standardised basis for comparing results across different studies and datasets. Performance metrics help determine how well a model can differentiate between disease stages, predict progression, or generalise to unseen data. Table 1 presents a comprehensive overview of evaluation metrics commonly used in AD diagnosis models, which can be broadly divided into two categories. Metrics such as Mean Absolute Error (MAE), Mean Squared Error (MSE), Root Mean Squared Error (RMSE), Root Mean Squared Logarithmic Error (RMSLE), Normalised MSE (NMSE), Correlation Coefficient (CC), Weighted Correlation, Adjusted Rand Index (ARI), Normalised Mutual Information (NMI), and Area Under the Curve (AUC). These metrics capture model performance in probabilistic, continuous, or unsupervised learning contexts, which play a crucial role in complex, data-rich healthcare applications.

Table 1 Performance evaluation for AD diagnosis

| Name | Equation | Interpretation |
| --- | --- | --- |
| Accuracy | $\frac{TP+TN}{TP+FP+TN+FN}$ | Overall percentage of correct predictions |
| Precision | $\frac{TP}{TP+FP}$ | The fractions that are positive among predicted positives |
| Recall  / Sensitivity /True Positive Rate | $\frac{TP}{TP+FN}$ | Fraction of actual positives correctly identified (range: 0–1; higher is better) |
| Specificity | $\frac{TN}{TN+FP}$ | Fraction of actual negatives correctly identified (range: 0–1; higher is better) |
| F1 score | $\frac{2}{REC^{-1}+PRE^{-1}}$ | Harmonic means precision and recall (range: 0–1; higher is better) |
| Matthews Correlation Coefficient | $\frac{TP\cdot TN-FP\cdot FN}{\sqrt{\left( TP+FP \right)\left( TP+FN \right)\left( TN+FP \right)(TN+FN)}}$ | Correlation of the confusion matrix (range: –1 to 1; closer to 1 is better) |
| Balanced Accuracy | $\frac{1}{2}\left( REC+SPE \right)=\frac{1}{2}\left( \frac{TP}{TP+FN}+\frac{TN}{TN+FP} \right)$ | Average of sensitivity and specificity (range: 0–1; higher is better) |
| Mean Absolute Error | $\frac{1}{n}\sum_{i=1}^{n} \left\vert y_{i}-\hat{y_{i}} \right\vert$ | Average magnitude of prediction errors for continuous outputs (non-negative; lower is better) |
| Mean Squared Error | $\frac{1}{n}\sum_{i=1}^{n} \left( y_{i}-\hat{y_{i}} \right)^{2}$ | Average squared prediction error (non-negative; lower is better) |
| Normalised Mean Squared Error | $\frac{MSE}{\sigma_{y}^{2}}$ where $\sigma_{y}^{2}=Var\left( y \right)$ | Mean Squared Error normalised by the variance of the target variable (lower is better) |
| Mean Squared Error | $\sqrt{MSE}$ | Square root of Mean Squared Error (non-negative; lower is better) |
| Root Mean Squared Logarithmic Error | $\sqrt{\frac{1}{n}\sum_{i=1}^{n} \left[ \ln\left( y_{i}+1 \right)-\ln\left( \hat{y_{i}}+1 \right) \right]^{2}}$ | Root Mean Squared Error on a log scale (non-negative; lower is better) |
| Normalised Mutual Information | $\frac{2I\left( X; Y \right)}{H\left( X \right)+H\left( Y \right)}$ $I$ is mutual information | Information‑theoretic similarity between predicted and true labels (range: 0–1; higher is better) |
| Adjusted Rand Index | $\frac{\sum_{i, j} \left( \begin{aligned} N_{ij} \\ 2 \end{aligned} \right)-\frac{\sum_{i} \left( \begin{aligned} N_{i} \\ 2 \end{aligned} \right)\sum_{j} \left( \begin{aligned} N_{j} \\ 2 \end{aligned} \right)}{\left( \begin{aligned} N \\ 2 \end{aligned} \right)}}{\frac{1}{2}\left[ \sum_{i} \left( \begin{aligned} N_{i} \\ 2 \end{aligned} \right)+\sum_{j} \left( \begin{aligned} N_{j} \\ 2 \end{aligned} \right) \right]-\frac{\sum_{i} \left( \begin{aligned} N_{i} \\ 2 \end{aligned} \right)\sum_{j} \left( \begin{aligned} N_{j} \\ 2 \end{aligned} \right)}{\left( \begin{aligned} N \\ 2 \end{aligned} \right)}}$ | Measures agreement between clustering (range: 0–1; higher is better) |
| Correlation Coefficient | $\frac{n\sum x_{i}y_{i}-\sum x_{i}\sum y_{i}}{\sqrt{n\sum x_{i}^{2}-\left( \sum x_{i} \right)^{2}}\sqrt{n\sum y_{i}^{2}-\left( \sum y_{i} \right)^{2}}}$ | Linear correlation between two continuous variables (range: –1 to 1; closer to 1 is better) |
| Weighted Correlation Coefficient | $corr\left( x,y;w \right)=\frac{cov\left( x,y;w \right)}{\sqrt{cov\left( x,x;w \right)cov\left( y,y;w \right)}}$ where $w$ represents a weight vector | Pearson correlation that assigns weights to observations (range: –1 to 1; closer to 1 is better) |
| Area Under ROC Curve | $\frac{1}{\left\vert P \right\vert\cdot\left\vert N \right\vert}\sum_{p\in P} \sum_{n\in N} \left[ 1\left( s\left( p \right)>s\left( n \right) \right)+\frac{1}{2}\cdot1\left( s\left( p \right)=s\left( n \right) \right) \right]$ | The probability that a classifier ranks a random positive classification higher than a random negative classification (range: 0–1; higher is better) |

TP: number of correctly identified positive cases. TN: number of correctly identified negative cases. FP: number of negative cases incorrectly identified as positive. FN: number of positive cases incorrectly identified as negative. $y_{i}$: true (actual) value of the 𝑖-th observation. $\hat{y_{i}}$: the predicted value produced by the model for the 𝑖-th observation. *H(X):* the entropy of X, which measures the amount of uncertainty or randomness in variable X. 𝐻(𝑌): the entropy of Y, which measures the amount of uncertainty or randomness in variable Y. *N*: the total number of data points, N_ij_: the number of points shared between cluster C_i_ in partition P and class C_j_^*^ in ground truth partition P^*^. *s(p):* the score (or prediction probability) assigned by the model to a positive sample. *s(n):* the score assigned to a negative sample.
