# Supplementary material for: Multimodal AI for Alzheimer Disease Diagnosis: Systematic Review of Datasets, Models, and Modalities
Source: J Med Internet Res. 2026 Mar 25;28:e85414. doi: 10.2196/85414 (PMC13018777; doi:10.2196/85414)
Supplement: Multimedia Appendix 3 — Complete QUADAS-2 risk-of-bias assessments for all included studies, summarizing judgments across patient selection, index test, reference standard, and flow or timing, with detailed study-level ratings. QUADAS-2: Revised Quality Assessment of Diagnostic Accuracy Studies Tool. [file jmir-v28-e85414-s003.docx]

# QUADAS-2 Risk of Bias Table

| **Study** | **Patient selection** | **Index test** | **Reference standard** | **Flow & timing** |
| --- | --- | --- | --- | --- |
| Xue et al., 2024 | High | Low | Low | Unclear |
| Shi et al., 2018 | High | Unclear | Unclear | Unclear |
| Allwright et al., 2023 | Low | Unclear | Unclear | Low |
| Gu et al., 2025 | Low | High | Unclear | Low |
| You et al., 2022 | Low | Unclear | Low | Low |
| Calvo et al., 2024 | Low | Low | Low | Unclear |
| Yi et al., 2025 | High | Unclear | Low | Unclear |
| Yousefzadeh et al., 2024 | Low | Unclear | Low | Unclear |
| Gong et al., 2023 | Low | Unclear | Low | Unclear |
| Lian et al., 2022 | High | High | Unclear | Unclear |
| Lian et al., 2022 | High | High | Unclear | Unclear |
| Li et al., 2019 | High | Unclear | Unclear | Unclear |
| Qiu et al., 2022 | High | Unclear | Unclear | Unclear |
| Oh et al., 2023 | High | Unclear | Unclear | Unclear |
| Lian et al., 2020 (H-FCN) | High | Unclear | Unclear | Unclear |
| Avsec et al., 2021 (Enformer) | Unclear | Unclear | Unclear | Unclear |
| Yang et al., 2021 | High | Unclear | Unclear | Unclear |
| Lee et al., 2024 | High | Unclear | Unclear | Unclear |
| Zhu et al., 2021 (DA-MIDL) | High | High | Unclear | Unclear |
| Zhang et al., 2024 | High | Unclear | Unclear | Unclear |
| Velazquez & Lee, 2022 | High | High | Unclear | Unclear |
| Zhang et al., 2024 | High | High | Unclear | Unclear |
| Bi et al., 2020 | High | Unclear | Unclear | Unclear |
| Bi et al., 2022 | High | High | Unclear | Unclear |
| Hashmi & Barukab, 2023 | High | High | Unclear | Unclear |
| Wang et al., 2024 | High | High | Unclear | Unclear |
| Hatami et al., 2024 | High | Unclear | Unclear | Unclear |
| Tabarestani et al., 2020 | High | Unclear | Low | Low |
| Burkhart et al., 2024 | High | Unclear | Low | Low |
| El-Sappagh et al., 2021 | High | Unclear | Low | Unclear |
| Lee et al., 2024 | High | Unclear | Unclear | Unclear |
| Yuan et al., 2021 | High | Unclear | Unclear | Unclear |
| Cirincione et al., 2024 | High | Unclear | Unclear | Unclear |
| Cassani & Falk, 2020 | Unclear | Unclear | Unclear | Unclear |
| Cilia et al., 2021 | Unclear | Unclear | Unclear | Unclear |
| Kmetzsch et al., 2022 | Unclear | Unclear | Unclear | Unclear |
| Mengoudi et al., 2020 | Unclear | Unclear | Unclear | Unclear |
| Tsai et al., 2024 (MAND) | Unclear | Unclear | Unclear | Low |
| Park et al., 2024 | Low | Unclear | Unclear | Unclear |
| Wu et al., 2022 | Unclear | Unclear | Unclear | Unclear |
| Zhang et al., 2025 | High | Unclear | Unclear | Unclear |
| Fabietti et al., 2023 | Unclear | Unclear | Low | Unclear |
| Seifallahi et al., 2022 | Unclear | Unclear | Low | Unclear |
| Fan et al., 2024 | Unclear | Unclear | Unclear | Unclear |
| Beebe-Wang et al., 2021 | Low | Unclear | Unclear | Low |
| Battineni et al., 2021 | High | Unclear | Unclear | Unclear |

# QUADAS-2 Risk of Bias – Table 4 (Linguistic/Speech Multimodal Studies)

| Study | Patient selection | Index test | Reference standard | Flow & timing |
| --- | --- | --- | --- | --- |
| Ilias & Askounis, 2023 | High | Unclear | Low | Unclear |
| Far Poor et al., 2024 | Low | High | Low | Unclear |
| Lin & Washington, 2024 | High | High | Low | Unclear |
| Ortiz-Perez et al., 2023 | High | Unclear | Low | Unclear |
| Ilias & Askounis, 2022 | High | High | Low | Unclear |
| Wen et al., 2023 | High | Unclear | Low | Unclear |
| Chen et al., 2023 | High | Unclear | Low | Unclear |
| Zheng et al., 2022 | High | Unclear | Low | Unclear |
| Nambiar et al., 2022 | High | Unclear | Low | Unclear |
| Priyadarshinee et al., 2023 | High | High | Low | Unclear |
| Liu et al., 2023 | High | Unclear | Low | Unclear |
| Shah et al., 2023 | High | Unclear | Low | Unclear |
| Mahajan & Baths, 2021 | High | High | Low | Unclear |
| Mei et al., 2023 | High | Unclear | Low | Unclear |
| Meerza et al., 2022 | High | Unclear | Low | Unclear |
| Chen et al., 2023 | High | Unclear | Low | Unclear |
| Ilias et al., 2023 | High | High | Low | Unclear |
| Tamm et al., 2023 | High | Unclear | Low | Unclear |
| Hlédiková, Woszczyk et al., 2022 | High | Unclear | Low | Unclear |
| Jin et al., 2023 | High | Unclear | Low | Unclear |
