# Supplementary material for: Multimodal AI for Alzheimer Disease Diagnosis: Systematic Review of Datasets, Models, and Modalities
Source: J Med Internet Res. 2026 Mar 25;28:e85414. doi: 10.2196/85414 (PMC13018777; doi:10.2196/85414)
Supplement: Multimedia Appendix 4 — Overview of traditional machine-learning models applied in Alzheimer disease research, including SVM, decision trees, HMMs, KNN, logistic regression, GMMs, and foundational CNN or RL descriptions, with methodological principles and limitations. CNN: convolutional neural network; GMM: gaussian mixture models; HMM: hidden markov model ; KNN: k-nearest neighbors; RL: reinforcement learning; SVM: support vector machine. [file jmir-v28-e85414-s004.docx]

## Traditional machine learning models

**Support Vector Machine (SVM)** is a supervised learning model initially proposed by Cortes [1], widely utilised for both classification and regression tasks. The core principle of SVM involves identifying an optimal hyperplane in a high-dimensional feature space that maximally separates data points from different classes. Due to its robust generalisation capabilities, SVM has been extensively applied in computer-aided diagnosis systems, particularly for medical image classification and disease detection [2], [3], [4], [5], [6]). However, SVM exhibits notable limitations under certain conditions. Specifically, in scenarios where the dimensionality of the feature space exceeds the number of training samples, the model is prone to overfitting. Additionally, conventional SVM does not inherently provide probabilistic outputs, which constrains its interpretability in clinical decision-making processes. These drawbacks underscore the need for hybrid or enhanced models that integrate SVM with dimensionality reduction techniques or probabilistic calibration methods to improve performance and interpretability.

**Decision Tree (DT)** is a widely utilised, interpretable machine learning technique applicable to both classification and regression tasks. It operates by recursively partitioning the feature space based on a set of decision rules, which are derived from prior probabilities and the expected value of target outcomes. Due to its inherent simplicity and transparency, DT models are frequently employed in scenarios involving datasets with high proportions of missing values [7], [8], [9], [10]. Their intuitive structure requires minimal domain-specific expertise and is computationally efficient, making them particularly suitable for preliminary data exploration and decision support systems in clinical and diagnostic settings. However, a well-known limitation of decision trees is their tendency to overfit, especially when applied to large datasets with complex, non-linear relationships, unless appropriately regularised through techniques such as pruning or ensemble learning.

**Hidden Markov Model (HMM)** is a classical machine learning framework widely employed in both classification and regression tasks. In HMMs, the underlying system is modelled as a set of hidden states that are not directly observable, while the observable outputs are probabilistically dependent on these latent states. Each hidden state is associated with a specific probability distribution over the set of possible observed outputs. By generating a sequence of observations, the model provides insights into the underlying sequence of hidden states. Owing to its ability to effectively model temporal dependencies and stochastic processes, HMM is particularly suitable for analysing series data. Consequently, it has been extensively applied to computer-aided diagnosis of AD [11], [12], [13], [14], where temporal patterns in verbal communication can serve as critical biomarkers for early detection and disease progression monitoring.

In addition to HMM-based approaches, various other machine learning algorithms such as k-Nearest Neighbors (KNN), Logistic Regression (LR), and Gaussian Mixture Models (GMMs) have been applied in the field of AI to assist in the diagnosis of AD [15], [16], [17], [18], [19]. Although these traditional models have demonstrated effectiveness in specific tasks, they often struggle to capture the complex and comprehensive features present in large-scale multimodal datasets. To overcome these limitations, researchers have proposed deep learning models that can automatically extract rich and latent feature representations from diverse data sources.

**Convolutional Neural Network (CNN)** is specifically designed for the analysis of image data, leveraging spatial hierarchies by capturing local patterns through convolutional operations. By exploiting spatial correlations among adjacent pixels, CNNs are particularly effective in extracting discriminative features from medical imaging. Given that neuroimaging modalities play a critical role in the diagnosis and staging of AD, a wide range of CNN architectures and their variants have been proposed. These models have demonstrated high performance in AD classification and prediction tasks, often surpassing traditional approaches in terms of accuracy and robustness [20], [21], [22], [23], [24]. However, despite their widespread adoption, CNNs exhibit several limitations beyond overfitting. Firstly, their inherent inductive bias toward locality and translation invariance, while effective for capturing low-level features, may restrict their capacity to model long-range dependencies and global contextual information that an aspect increasingly relevant in capturing subtle neurodegenerative patterns across distributed brain regions. Secondly, CNNs often require extensive architecture tuning and large labelled datasets to achieve generalisability, making them less flexible in data-scarce medical contexts, and challenges remain in balancing computational efficiency, interpretability, and performance on small, heterogeneous clinical datasets.

## Reinforcement learning

Reinforcement learning (RL), a subfield of machine learning, is centred on the concept of enabling an agent to learn an optimal policy through continuous interaction with its environment, with the objective of maximising cumulative rewards. Given that AD is a chronic, progressive neurodegenerative disorder, its long-term management and the need for personalised intervention strategies align well with the sequential decision-making capabilities inherent in RL. A core component of RL is the value function, which estimates the expected cumulative benefit of a given state or action. In recent applications to AD, researchers have begun using deep neural networks to approximate value functions, enabling the agent to handle complex, high-dimensional patient data. By simulating the dynamic process of “treatment–feedback–optimisation,” RL offers a more adaptive and flexible framework for devising individualised, long-term therapeutic strategies in AD care [25], [26]).

1. Cortes C, Vapnik V. Support-vector networks. Mach Learn 1995 Sept;20(3):273–297. doi: 10.1007/BF00994018

2. Sharma A, Kaur S, Memon N, Jainul Fathima A, Ray S, Bhatt MW. Alzheimer’s patients detection using support vector machine (SVM) with quantitative analysis. Neuroscience Informatics 2021 Nov;1(3):100012. doi: 10.1016/j.neuri.2021.100012

3. Park B, Kim Y, Park J, Choi H, Kim S-E, Ryu H, Seo K. Integrating Biomarkers From Virtual Reality and Magnetic Resonance Imaging for the Early Detection of Mild Cognitive Impairment Using a Multimodal Learning Approach: Validation Study. J Med Internet Res 2024 Apr 17;26:e54538. doi: 10.2196/54538

4. Gao X, Liu H, Shi F, Shen D, Liu M. Brain Status Transferring Generative Adversarial Network for Decoding Individualized Atrophy in Alzheimer’s Disease. IEEE J Biomed Health Inform 2023 Oct;27(10):4961–4970. doi: 10.1109/JBHI.2023.3304388

5. Romano MS, Tuch SAN, Bustamante LMC. Selection of Acoustic, Temporal, and Complexity Features for Machine Learning Classification of Alzheimers Dissease on a Spanish Population Through Automatic Analysis of Reading-Elicited Speech.

6. Lazli L. Improved Alzheimer Disease Diagnosis With a Machine Learning Approach and Neuroimaging: Case Study Development. JMIRx Med 2025 Apr 21;6:e60866–e60866. doi: 10.2196/60866

7. Hossain F, Halder RK, Uddin MN. An integrated machine learning based adaptive error minimization framework for Alzheimer’s stage identification. Intelligence-Based Medicine 2025;11:100243. doi: 10.1016/j.ibmed.2025.100243

8. Fulkar B, Dhale T, Pacharaney U, Deshmukh S. Early Detection of Chronic Diseases Using Machine and Deep Learning Algorithms. 2025 4th International Conference on Sentiment Analysis and Deep Learning (ICSADL) Bhimdatta, Nepal: IEEE; 2025. p. 1656–1661. doi: 10.1109/ICSADL65848.2025.10933005

9. Sathiya A, Basha CH, S Vasanth, Sharmila P JJ, S P, Indhumathi R. Enhancing Alzheimer’s Disease Detection Using Optimized Attribute Selection and Random Forest Classifier for Improved Accuracy. 2025 International Conference on Visual Analytics and Data Visualization (ICVADV) Tirunelveli, India: IEEE; 2025. p. 1174–1179. doi: 10.1109/ICVADV63329.2025.10961844

10. Saleh AW, Gupta G, Khan SB, Alkhaldi NA, Verma A. An Alzheimer’s disease classification model using transfer learning Densenet with embedded healthcare decision support system. Decision Analytics Journal 2023 Dec;9:100348. doi: 10.1016/j.dajour.2023.100348

11. Baucum M, Khojandi A, Papamarkou T. Hidden Markov models as recurrent neural networks: An application to Alzheimer’s disease. 2021 IEEE 21st International Conference on Bioinformatics and Bioengineering (BIBE) Kragujevac, Serbia: IEEE; 2021. p. 1–6. doi: 10.1109/BIBE52308.2021.9635256

12. Wijeratne PA, Alexander DC. Learning transition times in event sequences: the Event-Based Hidden Markov Model of disease progression. arXiv; 2021. doi: 10.48550/arXiv.2011.01023

13. Cai Z, Zeng D, Marder KS, Honig LS, Wang Y. Dynamic Classification of Latent Disease Progression with Auxiliary Surrogate Labels. arXiv; 2024. doi: 10.48550/ARXIV.2412.08088

14. Chen Y, Pham TD. Development of a brain MRI-based hidden Markov model for dementia recognition. BioMed Eng OnLine 2013;12(Suppl 1):S2. doi: 10.1186/1475-925X-12-S1-S2

15. Vats NA, Yadavalli A, Gurugubelli K, Vuppala AK. ACOUSTIC FEATURES, BERT Model AND THEIR COMPLEMENTARY NATURE FOR ALZHEIMER’S DEMENTIA DETECTION. 2021 Thirteenth International Conference on Contemporary Computing (IC3-2021) Noida India: ACM; 2021. p. 267–272. doi: 10.1145/3474124.3474162

16. Xiao R, Cui X, Qiao H, Zheng X, Zhang Y, Zhang C, Liu X. Early diagnosis model of Alzheimer’s disease based on sparse logistic regression with the generalized elastic net. Biomedical Signal Processing and Control 2021 Apr;66:102362. doi: 10.1016/j.bspc.2020.102362

17. Shah Z, Qi S-A, Wang F, Farrokh M, Tasnim M, Stroulia E, Greiner R, Plitsis M, Katsamanis A. Exploring Language-Agnostic Speech Representations Using Domain Knowledge for Detecting Alzheimer’s Dementia. ICASSP 2023 - 2023 IEEE International Conference on Acoustics, Speech and Signal Processing (ICASSP) Rhodes Island, Greece: IEEE; 2023. p. 1–2. doi: 10.1109/ICASSP49357.2023.10095593

18. Ablimit A, Botelho C, Abad A, Schultz T, Trancoso I. Exploring Dementia Detection from Speech: Cross Corpus Analysis. ICASSP 2022 - 2022 IEEE International Conference on Acoustics, Speech and Signal Processing (ICASSP) Singapore, Singapore: IEEE; 2022. p. 6472–6476. doi: 10.1109/ICASSP43922.2022.9747167

19. Lahmiri S. Integrating convolutional neural networks, kNN, and Bayesian optimization for efficient diagnosis of Alzheimer’s disease in magnetic resonance images. Biomedical Signal Processing and Control 2023 Feb;80:104375. doi: 10.1016/j.bspc.2022.104375

20. Suwalka D, Pandita D, Godse S, Patil RR, Salam Khan A, Kumar A. AI Applications and Simulation-Based Learning Integrating Future of Nursing Education. 2024 International Conference on Intelligent &amp; Innovative Practices in Engineering &amp; Management (IIPEM) Singapore, Singapore: IEEE; 2024. p. 1–6. doi: 10.1109/IIPEM62726.2024.10925639

21. Chaudhari A, Saratkar S, Thute T. AI-Enhanced Imaging Techniques for Understanding Alzheimer’s Progression. 2025 International Conference on Machine Learning and Autonomous Systems (ICMLAS) Prawet, Thailand: IEEE; 2025. p. 1174–1179. doi: 10.1109/ICMLAS64557.2025.10969042

22. Ango R, C KKR, Fatima S, Nag A. Brain Connectivity Analysis in Alzheimer’s disease using Graph Convolutional Network. 2024 4th International Conference on Soft Computing for Security Applications (ICSCSA) Salem, India: IEEE; 2024. p. 133–139. doi: 10.1109/ICSCSA64454.2024.00028

23. Chattopadhyay T, Joshy NA, Ozarkar SS, Buwa KS, Feng Y, Laltoo E, Thomopoulos SI, Villalon‐Reina JE, Joshi H, Venkatasubramanian G, John JP, Thompson PM. Deep Learning Algorithms for Alzheimer’s Disease Detection based on Diffusion MRI: Tests in Indian and North American Cohorts. Alzheimer’s &amp; Dementia 2024 Dec;20(S2):e089294. doi: 10.1002/alz.089294

24. Ma D, Zhang H, Wang L. Editorial: Deep learning methods and applications in brain imaging for the diagnosis of neurological and psychiatric disorders. Front Neurosci 2024 Oct 1;18:1497417. doi: 10.3389/fnins.2024.1497417

25. Xiao X, Li Y, Wu Q, Liu X, Cao X, Li M, Liu J, Gong L, Dai X. Development and validation of a novel predictive model for dementia risk in middle-aged and elderly depression individuals: a large and longitudinal machine learning cohort study. Alz Res Therapy 2025 May 13;17(1):103. doi: 10.1186/s13195-025-01750-6

26. Williams C, Anik FI, Hasan MdM, Rodriguez-Cardenas J, Chowdhury A, Tian S, He S, Sakib N. Advancing Brain-Computer Interface Closed-Loop Systems for Neurorehabilitation: A Systematic Review of AI and Machine Learning Innovations in Biomedical Engineering (Preprint). JMIR Biomedical Engineering; 2025. doi: 10.2196/preprints.72218
