# Supplementary material for: Multimodal AI for Alzheimer Disease Diagnosis: Systematic Review of Datasets, Models, and Modalities
Source: J Med Internet Res. 2026 Mar 25;28:e85414. doi: 10.2196/85414 (PMC13018777; doi:10.2196/85414)
Supplement: Multimedia Appendix 5 — Cochrane Handbook 5.3.3–aligned data-extraction tables summarizing study design, datasets, participants, modalities, preprocessing, model architectures, validation schemes, outcomes, and limitations for all included studies. [file jmir-v28-e85414-s005.docx]

# Cochrane 5.3.3 Extraction – Xue et al., 2024

| Field | Details |
| --- | --- |
| Study ID | Xue et al., 2024 |
| Publication Year | 2024 |
| Country / Setting | Multi-country datasets (mainly USA) |
| Study Design | Multicohort diagnostic model development & external validation |
| Datasets Used | NACC, ADNI, AIBL, FHS, PPMI, OASIS, LBDSU, 4RTNI, NIFD |
| Recruitment Period | Varies by dataset; retrospective |
| Inclusion Criteria | Individuals with cognitive assessments, clinical data, MRI |
| Exclusion Criteria | Dataset-specific; not consistently reported |
| Sample Size | 51,269 total participants |
| Group Distribution | NC, MCI, Dementia; 10 etiologies |
| Participant Demographics | Age ~60–85; mixed sex; majority White |
| Modalities Used | Demographics, clinical history, neuropsych tests, MRI, PET |
| Acquisition Details | Multisequence MRI (T1w, T2w, FLAIR); PET where available |
| Preprocessing | Feature embedding, harmonization, missing feature masking |
| Model Architecture | Transformer-based multimodal model |
| Training & Validation | Internal on NACC; external on ADNI & FHS |
| Outcomes | Differential diagnosis of dementia etiologies |
| Results | AUROC 0.94–0.96; aligned with biomarkers & neuropathology |
| Missing Data Handling | Random feature masking; robust to 60–90% missingness |
| Risk of Bias / Limitations | Imbalanced etiologies; limited racial diversity; dataset variability |
| Funding / Conflicts | Reported in paper |

# [101] Shi et al., 2018, IEEE JBHI

| Field | Details |
| --- | --- |
| Study ID | Shi et al., 2018 |
| Publication Year | 2018 |
| Country / Setting | United States — ADNI (Alzheimer’s Disease Neuroimaging Initiative), multi-centre neuroimaging dataset |
| Study Design | Method development + retrospective analysis using ADNI neuroimaging dataset |
| Datasets Used | ADNI (MRI + PET; ROI-based features) |
| Recruitment Period | Not specified (ADNI baseline data) |
| Inclusion Criteria | ADNI subjects with:  MRI and PET available; Diagnostic labels: AD, MCI, MCI-C, MCI-NC, NC |
| Exclusion Criteria | Not explicitly stated (implicitly: missing MRI/PET data excluded) |
| Sample Size | Total = 202 participants (51 AD, 99 MCI (43 MCI-C, 56 MCI-NC) and 52 NC) |
| Group Distribution | 4 groups (AD, MCI-C, MCI-NC, NC) |
| Participant Demographics | Not reported (age/sex not provided in the article) |
| Modalities Used | T1 MRI (structural); FDG-PET (ROI-based features: 93 MRI ROI volumes + 93 PET ROI intensities) |
| Acquisition Details | MRI preprocessing steps:  AC–PC correction; N3 bias-field correction; Skull-stripping & cerebellum removal; FAST segmentation (GM/WM/CSF); HAMMER registration; 93 ROI parcellation (Kabani template)  PET: Rigid registration to MRI; I mean intensity extraction |
| Preprocessing | ROI extraction yielding 93 features per modality; Features normalised (not explicitly stated but implied) |
| Model Architecture | Multimodal Stacked Deep Polynomial Network (MM-SDPN): Stage 1: SDPN per modality (MRI, PET)  Stage 2: SDPN on concatenated features learned  DPN layers with PCA + OLS-based selection |
| Training & Validation | Compared models: DPN-3, DPN-6; SDPN (MRI, PET, combined); MM-SDPN  Classification tasks:  AD vs NC  MCI vs NC  MCI-C vs MCI-NC  4-class classification |
| Outcomes | Classification accuracy across tasks |
| Results | MM-SDPN outperformed:  Single-modality DPN; Single-modality SDPN; Concatenated SDPN; 9 state-of-the-art multimodal learning models |
| Missing Data Handling | Not reported (implicit exclusion of incomplete MRI/PET subjects) |
| Risk of Bias / Limitations | ADNI-only dataset (limits generalizability); ROI-based features rather than voxelwise/whole-brain; Lacked external validation |
| Funding / Conflicts | Supported by: National Natural Science Foundation of China; Shenzhen & Guangdong R&D grants.  No conflicts declared |

[59]

| Field | Details |
| --- | --- |
| Study ID | Allwright et al., “Ranking the risk factors for Alzheimer’s disease; findings from the UK Biobank study”, Aging Brain, 2023 |
| Publication Year | 2023 |
| Country / Setting | United Kingdom; community-based cohort using UK Biobank |
| Study Design | Prospective population cohort analysis using machine-learning risk modelling |
| Datasets Used | UK Biobank baseline assessments, linked hospital ICD-10 records, biochemical tests, cognitive tests, and genetic data |
| Recruitment Period | 2006–2010 baseline recruitment; dementia outcomes followed up to 2021 |
| Inclusion Criteria | Participants aged 60–70 at baseline with genotype data and no dementia diagnosis at study entry |
| Exclusion Criteria | Baseline dementia, dementia diagnosis within 2 years of baseline, prior Parkinson’s disease, death from non-dementia causes before 10-year window |
| Sample Size | 156,209 total participants; 2,090 developed AD within 2–10 years |
| Group Distribution | AD cases: 2,090; controls: 154,119; age-matched case–control sampling |
| Participant Demographics | Age 60–70; mixed sex; predominantly White ethnicity (>95%) |
| Modalities Used | Demographic, socioeconomic, lifestyle, biochemical, cognitive, medical history, and genetic variables |
| Acquisition Details | Standardised UK Biobank baseline assessments; hospital ICD-10 inpatient records; genotyping via UKB Axiom array |
| Preprocessing | Variables with <20% missingness retained; categorical features one-hot encoded; missing values imputed using miceforest random-forest MICE; APOE-ε4 dosage computed from rs429358/rs7412 |
| Model Architecture | XGBoost gradient-boosted tree classifier with SHAP-based feature importance ranking |
| Training & Validation | Age-matched resampling of controls; nested 3-fold cross-validation repeated 50 times; hyperparameter tuning via grid search; external evaluation on held-out validation set |
| Outcomes | Prediction of incident Alzheimer’s disease within 2–10 years and ranking of risk/ protective factors |
| Results | Model AUROC 0.77 in the full cohort; outperforming literature-based risk models; APOE-ε4 strongest risk factor; liver enzymes, triglycerides, socioeconomic and frailty indicators among additional top predictors |
| Missing Data Handling | Multiple imputation using miceforest; exclusion of high-missingness variables |
| Risk of Bias / Limitations | ICD-10–based AD diagnosis may under-ascertain community cases; healthy-volunteer bias; predominantly White cohort; observational ML analysis cannot infer causality |
| Funding / Conflicts | Supported by Australian NHMRC, University of Sydney SOAR Fellowships, and UK Biobank; authors reported no conflicts of interest |

[60]

| Field | Details |
| --- | --- |
| Study ID | Gu et al., 2025 |
| Publication Year | 2025 |
| Country / Setting | United Kingdom; population-based prospective cohort (UK Biobank) |
| Study Design | Longitudinal prospective cohort; machine-learning dementia risk prediction model development and temporal validation |
| Datasets Used | UK Biobank (baseline clinical, demographic, biological assays, cognitive tests, physical measures, follow-up hospital/GP/death registry data) |
| Recruitment Period | UK Biobank recruited 2006–2010; follow-up through April 2023 |
| Inclusion Criteria | Adults aged 40–69 at baseline with established ASCVD (CAD, CeVD, PAD, AAA) and ≥10 years of follow-up data |
| Exclusion Criteria | Baseline dementia (n=64), no follow-up records (n=69), death before follow-up (n=8532) |
| Sample Size | 29,561 participants with ASCVD after quality control |
| Group Distribution | No dementia: 28,227; All-cause incident dementia: 1334; AD: 425; VD: 536 |
| Participant Demographics | Median age 62 years; 36.63% women; 94.12% White ethnicity |
| Modalities Used | Demographics, lifestyle questionnaires, physical measurements, cognitive function tests, biological assays (blood biomarkers) |
| Acquisition Details | Baseline health assessments, touchscreen questionnaires, clinic-based physical measures, blood sample assays; follow-up via GP records, hospital inpatient data, death registry |
| Preprocessing | Removal of variables with >40% missing; random under sampling; class-weight adjustments; multiple imputation; variable generation from available data; removal of correlated predictors via hierarchical clustering (ρ > 0.75) |
| Model Architecture | Multiple ML models tested: LightGBM (final model), XGBoost, Random Forest, Logistic Regression, KNN, SVM, ANN; final chosen model: LightGBM-based ASCVD dementia risk model |
| Training & Validation | Nested cross-validation; temporal validation (train 2006-2009, test 2010 cohort); hyperparameter tuning over 10,000 candidate sets |
| Outcomes | Primary: All-cause incident dementia; Secondary: AD and VD incidence at 5-year, 10-year, and overall follow-up intervals |
| Results | AUC: 0.866 (training); 0.819 (temporal test set). For 5-year predictions: dementia AUC 0.903; AD 0.775; VD 0.803. Accuracy (test): 0.851. Sensitivity: 0.691; Specificity: 0.866. |
| Missing Data Handling | Multiple imputation; exclusion of variables with >40% missing; undersampling of majority class; class-weight adjustment in ML models |
| Risk of Bias / Limitations | Sample mostly European descent; potential overfitting in LightGBM; static baseline features; possible unmeasured competing risks; limited generalizability outside UK/European populations |
| Funding / Conflicts | Not explicitly detailed in the PDF text; uses UK Biobank infrastructure with ethical approval and standard data access frameworks |

61

| Field | Details |
| --- | --- |
| Study ID | You et al., 2022 |
| Publication Year | 2022 |
| Country / Setting | United Kingdom (UK Biobank population-based cohort) |
| Study Design | Longitudinal, prospective, population-based machine-learning cohort study; model development with internal validation |
| Datasets Used | UK Biobank (UKB) |
| Recruitment Period | March 1, 2006 – October 31, 2010 |
| Inclusion Criteria | Adults aged 40–69 at baseline, completed baseline assessments, no dementia or stroke at baseline, with ≥10 years follow-up |
| Exclusion Criteria | Prevalent dementia (n=890), prevalent stroke (n=7,184), missing follow-up data (n=70,071) |
| Sample Size | 425,159 included participants |
| Group Distribution | Healthy controls: 419,872; Incident dementia: 5,287; Incident AD: 2,416 |
| Participant Demographics | Mean age 58 years; 54.4% female; predominantly White ethnicity (94.3%) |
| Modalities Used | Demographics, lifestyle/health questionnaires, physical measures, cognitive tests, blood biomarkers, genetics (ApoE, PRS) |
| Acquisition Details | Standardized baseline UKB assessments including touchscreen lifestyle measures, physical measurements, cognitive tasks, genotyping arrays, blood sample assays |
| Preprocessing | Exclusion of variables with >40% missingness; manual removal of clinically irrelevant variables; no imputation (LightGBM handles missingness automatically) |
| Model Architecture | LightGBM gradient boosting decision tree classifier; isotonic regression for probability calibration |
| Training & Validation | Five-fold cross-validation; hyperparameter tuning using 1000 candidate sets; SHAP for model interpretability |
| Outcomes | \|  \| \| --- \|  \| Incident all-cause dementia; incident Alzheimer’s disease; prediction horizons at 5 years, 10 years, and full follow-up (median 11.9 years) \| \| --- \| |
| Results | Ten predictors selected: age, ApoE ε4, pairs matching time, leg fat percentage, number of medications taken, reaction time, peak expiratory flow, mother’s age at death, long-standing illness, mean corpuscular volume. AUC for all-cause dementia: 0.848 ± 0.007; AUC for AD: 0.862 ± 0.015. Good calibration (Hosmer–Lemeshow p=0.92). Outperformed CAIDE, DRS, ANU-ADRI (p<0.001). |
| Missing Data Handling | No imputation; LightGBM’s built-in missing value handling used |
| Risk of Bias / Limitations | Limited external validation; population predominantly White; potential residual confounding; feature selection fully data-driven; predictors like BMI and BP not selected despite prior evidence; dementia ascertainment limited by available EHR sources |
| Funding / Conflicts | Multiple funding sources from Chinese national programs, Fudan University, Shanghai agencies; authors declare no conflicts of interest |

62

| Field | Details |
| --- | --- |
| Study ID | Calvo et al., 2024 |
| Publication Year | 2024 |
| Country / Setting | United Kingdom; population-based cohort from UK Biobank |
| Study Design | Cross-sectional cohort analysis with multivariable logistic regression comparing menopause types and Alzheimer’s disease odds |
| Datasets Used | UK Biobank (baseline questionnaire, clinical, genetic, and linked ICD-9/10 hospital records) |
| Recruitment Period | Original UK Biobank recruitment 2006–2010; AD diagnoses ascertained from subsequent hospital records (follow-up into later life) |
| Inclusion Criteria | Women aged ≥60 years at baseline with valid menopause type and age data, classified as early bilateral oophorectomy (BO <49 years) or spontaneous menopause (SM ≥51 years), with or without AD diagnosis |
| Exclusion Criteria | Women with menopause age outside BO <49 or SM ≥51 bands; cases with other dementias, cerebral palsy, or withdrawn consent; very sparse comorbidities (diabetes, hypertension, depression, anxiety) excluded from regression modelling |
| Sample Size | 34,603 women total |
| Group Distribution | BO (no AD) n=4,356; BO-AD n=47; SM (no AD) n=30,139; SM-AD n=61 |
| Participant Demographics | Mean age 63.8 years (SD 2.79); mean education 13.0 years (SD 5.11); mean BMI 27.3 kg/m² (SD 4.87); 25.4% APOE4 carriers overall; high HT use in BO group (≈89%) |
| Modalities Used | Self-reported reproductive history (BO, SM, age at menopause, HT use), education, smoking; anthropometrics (BMI, body fat); medical history (cancer, diabetes, hypertension, depression, anxiety); APOE genotype |
| Acquisition Details | Menopause type and age, HT use, education and smoking obtained from baseline “female-specific” and lifestyle questionnaires; BMI and fat mass measured with Tanita BC418ma bioimpedance; diagnoses (diabetes, hypertension, depression, anxiety, cancer, AD) derived from UK Biobank hospital and cancer registries using ICD-9/10 codes; APOE genotype from UK BiLEVE and UK Biobank Axiom arrays using rs429358 and rs7412 |
| Preprocessing | APOE4 coded as dichotomous carrier vs non-carrier (ε2/ε4 grouped with non-carriers); HT use, cancer, diabetes, hypertension, depression, anxiety, smoking history coded as yes/no; education converted to years via ISCED categories; BMI calculated from measured height and weight; very low-frequency comorbidities removed from multivariable models |
| Model Architecture | Firth’s bias-reduced logistic regression models estimating odds of AD for combined menopause types and for BO-only and SM-only strata |
| Training & Validation | Single-cohort analysis; no train/test split; models adjusted simultaneously for menopause type (combined model), APOE4, HT use, age, years of education, age at menopause, BMI, cancer history, and smoking; additional sensitivity analysis including all women with SM >40 and BO <49 to disentangle effects of BO vs early menopause age |
| Outcomes | Primary outcome: odds of AD (ICD-9/10 codes) in relation to menopause type and candidate risk/resilience factors |
| Results | In the combined model, early BO associated with fourfold higher odds of AD vs SM (OR 4.12, 95% CI 2.02–8.44); APOE4 carriage (OR 2.55, 95% CI 1.76–3.69) and older age (OR 1.11 per year, 95% CI 1.05–1.19) increased odds of AD; ever HT use (OR 0.59, 95% CI 0.39–0.89), higher BMI (OR 0.94 per kg/m², 95% CI 0.90–0.98), and more years of education (OR 0.93 per year, 95% CI 0.90–0.97) were associated with reduced odds of AD. In the BO-only model, APOE4 (OR 4.29, 95% CI 2.43–7.56) and age (OR 1.16 per year, 95% CI 1.05–1.28) increased risk, while HT (OR 0.43, 95% CI 0.23–0.82), BMI (OR 0.93, 95% CI 0.87–0.99), and education (OR 0.91, 95% CI 0.85–0.98) were protective; in the SM-only model, only education showed a modest protective effect (OR 0.95, 95% CI 0.90–0.99) |
| Missing Data Handling | Analyses restricted to participants with complete data on included predictors; very rare comorbidities removed to avoid model instability; no explicit multiple imputation reported |
| Risk of Bias / Limitations | Unbalanced design with relatively few AD cases (especially BO-AD) leading to wide confidence intervals; limited number of very early BO cases (<38 years) may underestimate true risk; HT use self-reported without timing, type, or duration; inability to analyze depression and anxiety due to very low prevalence; UK Biobank healthy volunteer bias and under-representation of socioeconomically disadvantaged and non-White women, limiting generalizability |
| Funding / Conflicts | Funded by the Wilfred and Joyce Posluns Chair in Women’s Brain Health and Aging and associated Canadian agencies (CIHR, Ontario Brain Institute, CABHI, Alzheimer Society of Canada), Canadian Consortium on Neurodegeneration in Aging, and the Jacqueline Ford Gender and Health Fund; authors reported no conflicts of interest |

63

| Field | Details |
| --- | --- |
| Study ID | Yi et al., 2025 |
| Publication Year | 2025 |
| Country / Setting | UK Biobank (UK), plus external datasets from USA (ADNI), UK (IXI), and USA (PPMI) |
| Study Design | Multistage computational study: deep-learning brain age estimation + GWAS + MR + xQTL drug-target prioritization + drug repurposing |
| Datasets Used | UKB (n≈38,961 MRI), ADNI (n=1627), PPMI (n=259), IXI (n=313) |
| Recruitment Period | UKB MRI acquisition (2006–2010+ typical UKB period; not explicitly restated) |
| Inclusion Criteria | UKB participants with T1 MRI; “healthy” subset defined as no ICD-10-coded brain disorders for training; full UKB sample used in descriptive BAG analyses |
| Exclusion Criteria | Brain disorder diagnoses excluded from model training and BAG GWAS (e.g., AD, PD, MDD, SCZ, etc.) |
| Sample Size | 38,961 total MRI subjects; 29,097 healthy used for training; 31,520 with genotype for GWAS; external validation n=2199 |
| Group Distribution | Healthy vs. 12 brain-disorder groups (e.g., AD n=32, PD n=81, MDD n=1404, SCZ n=23, etc.) |
| Participant Demographics | Mean age ~64 years; 52.5% women (UKB) |
| Modalities Used | T1-weighted MRI; genomic SNP array; blood/brain eQTL; plasma pQTL |
| Acquisition Details | UKB standard T1 MRI, preprocessed & registered to MNI152; external datasets follow ADNI/PPMI/IXI standard pipelines |
| Preprocessing | MRI spatial normalization (MNI152), feature embedding from DL model; genotype QC; xQTL harmonization; MR using standard two-sample pipeline |
| Model Architecture | Seven DL models tested; best model = 3D Vision Transformer (3D-ViT) |
| Training & Validation | Training: 29,097 healthy UKB; Testing: 3227 healthy + 6637 disorder subjects; External validation: ADNI, PPMI, IXI with fine-tuning |
| Outcomes | Primary: Brain Age Gap (BAG = predicted brain age – chronological age); Secondary: GWAS loci, causal genes, druggable targets |
| Results | 3D-ViT MAE ≈ 2.6 years; BAG higher in disorders; GWAS identified 9 SNPs in 8 loci; 64 druggable genes identified; 7 high-confidence targets (MAPT, TNFSF12, GZMB, SIRPB1, GNLY, NMB, C1RL); 29 candidate drugs for repurposing |
| Missing Data Handling | Not explicitly described for MRI; GWAS/eQTL/pQTL uses standard missing-SNP and harmonization filters |
| Risk of Bias / Limitations | European-ancestry bias; brain age model lacks biological “ground-truth”; replication cohorts unavailable; complex heterogeneity of BAG & disorders |
| Funding / Conflicts | Reported in the paper (Sci Adv; multiple Chinese & Israeli academic institutions) |

64

| Study ID | Yousefzadeh et al., 2024 |
| --- | --- |
| Publication Year | 2024 |
| Country / Setting | United Kingdom; UK Biobank retina imaging cohort |
| Study Design | Model development & evaluation study using retinal fundus images; binary AD vs. NC classification + explainable AI + latent cluster analysis |
| Datasets Used | UK Biobank fundus imaging dataset (~170k images) |
| Recruitment Period | 2006–2010 baseline; retinal imaging ~2009 onward |
| Inclusion Criteria | AD subjects with high-quality fundus images; incidence AD only; age- and sex-matched controls |
| Exclusion Criteria | Poor-quality images; mixed dementia; Parkinsonism; prevalent AD; unclear retinal images |
| Sample Size | 200 images (100 AD; 100 NC) |
| Group Distribution | AD: 61 subjects; NC: 80 subjects |
| Participant Demographics | Age- and sex-matched; majority White ethnicity |
| Modalities Used | Fundus photos; vessel segmentation; vascular features; cognitive tests |
| Acquisition Details | UKB fundus protocol; AutoMorph segmentation; 224x224 resizing |
| Preprocessing | Quality filtering; segmentation; cropping; augmentation |
| Model Architecture | VGG-16 classifier + LAVA explainable AI |
| Training & Validation | Nested 5-fold CV; average accuracy 71.4% |
| Outcomes | AD vs. NC classification; neuron-level explanations; AD continuum clusters |
| Results | Accuracy 71.4%; 7 latent clusters; vascular + cognitive decline across continuum |
| Missing Data Handling | Mean imputation for cognitive tests; image exclusions |
| Risk of Bias / Limitations | Small AD sample; cross-sectional; limited generalizability; UKB volunteer bias |
| Funding / Conflicts | NSF grant 2123809; no conflicts declared |

65

| Study ID | Gong et al., 2023 |
| --- | --- |
| Publication Year | 2023 |
| Country / Setting | United Kingdom; UK Biobank multimodal MRI dataset |
| Study Design | Method development & evaluation study for supervised multimodal phenotype discovery |
| Datasets Used | UK Biobank brain imaging dataset (~39,770 subjects, 47 imaging modalities) |
| Recruitment Period | 2006–2010 baseline recruitment; imaging acquisition from ~2014 onward |
| Inclusion Criteria | Participants with usable multimodal MRI and available non-imaging phenotypes (nIDPs) |
| Exclusion Criteria | Poor-quality imaging; missing essential modalities; unusable scans |
| Sample Size | 39,770 participants |
| Group Distribution | General population cohort (no case–control grouping) |
| Participant Demographics | Middle-aged & older adults; mixed sex; predominantly UK population |
| Modalities Used | 47 modalities including T1, T2-FLAIR, swMRI, dMRI, task fMRI, resting-state fMRI |
| Acquisition Details | Data pre-processed with FSL & FreeSurfer; standardised UKB imaging protocols |
| Preprocessing | Voxel-wise normalization; confound regression (597 variables); dictionary learning for dimensionality reduction |
| Model Architecture | SuperBigFLICA: semi-supervised multimodal fusion model using Bayesian weighting |
| Training & Validation | Mini-batch SGD; training set = 25k; validation = 5k; test set = 9.7k; multiple λ and latent dimension tuning |
| Outcomes | Latent components predictive of 17,485 nIDPs; improved extraction of imaging-derived phenotypes |
| Results | Up to 46% improvement over expert-designed IDPs; 25–30% improvement over BigFLICA; interpretable multimodal modes |
| Missing Data Handling | Missing modalities imputed using modality mean maps; missing nIDPs not imputed |
| Risk of Bias / Limitations | UKB population bias; linear modelling constraints; limited generalisability outside UKB |
| Funding / Conflicts | Wellcome Trust, NWO, NIHR Oxford BRC; no conflicts reported |

66

| Study ID | Lian et al., 2022 (Attention-Guided HybNet) |
| --- | --- |
| Publication Year | 2022 |
| Country / Setting | Multicentre; USA (ADNI-1, ADNI-2) and Australia (AIBL) |
| Study Design | Deep-learning method development and evaluation for AD diagnosis and MCI conversion prediction using structural MRI |
| Datasets Used | ADNI-1, ADNI-2, and AIBL baseline T1-weighted structural MRI |
| Recruitment Period | ADNI-1 and ADNI-2 baseline (1.5T and 3T); AIBL baseline imaging wave |
| Inclusion Criteria | Baseline T1-weighted MRI available; clinical diagnosis of NC, MCI, or AD; for MCI, availability of follow-up to define sMCI vs pMCI within 36 months |
| Exclusion Criteria | Subjects appearing in both ADNI-1 and ADNI-2 removed from ADNI-2; scans failing preprocessing (e.g., registration/segmentation) excluded |
| Sample Size | Total 1,976 subjects: ADNI-1 (821), ADNI-2 (637), AIBL (519) |
| Group Distribution | ADNI-1: 229 NC, 226 sMCI, 167 pMCI, 199 AD; ADNI-2: 201 NC, 239 sMCI, 38 pMCI, 159 AD; AIBL: 447 NC, 72 AD |
| Participant Demographics | Older adults with suspected dementia; demographic variables include age, sex, education, and MMSE (summarized per cohort) |
| Modalities Used | Baseline structural T1-weighted MRI of the whole brain |
| Acquisition Details | ADNI-1 1.5T and ADNI-2 3T T1 MRI; AIBL 1.5T or 3T T1 MRI acquired under each study’s standard protocol |
| Preprocessing | AC–PC alignment (MIPAV), N3 bias correction, skull stripping (BET), cerebellum removal via template warping, linear registration to Colin27 (FLIRT), resampling to 1×1×1 mm³, cropping to 144×184×152 voxels |
| Model Architecture | Two-stage attention-guided CAD framework: backbone 3D fully convolutional network producing disease attention maps (DAMs), followed by a multibranch hybrid network (HybNet) combining a global branch on DAM-weighted feature maps and local patch-based branches |
| Training & Validation | Backbone FCN and HybNet trained on ADNI-1; independent evaluation on ADNI-2 and AIBL; comparisons against ROI-based SVM, VBM, DMIL, and H-FCN; performance measured by ACC, AUC, sensitivity, and specificity |
| Outcomes | Primary: AD vs NC classification; Secondary: pMCI vs sMCI conversion prediction |
| Results | Deep-learning models outperformed conventional ROI/VBM methods. HybNet achieved the best performance; for AD vs NC on ADNI-2, accuracy improved from 0.903 (H-FCN) to 0.919 and AUC from 0.951 to 0.965; similar gains observed for MCI conversion prediction and on AIBL |
| Missing Data Handling | Analyses restricted to subjects with usable baseline MRI and labels; no explicit statistical imputation of imaging data reported |
| Risk of Bias / Limitations | Use of research cohorts (ADNI/AIBL) with limited demographic diversity; only structural MRI modality; reliance on heavy preprocessing and linear registration; no external validation beyond ADNI/AIBL; possible overfitting to dataset-specific characteristics |
| Funding / Conflicts | Supported by NIH grants (e.g., AG041721, AG053867); authors reported no conflicts of interest in the article |

67

| Study ID | Lian et al., 2022 (MWAN – Multi-Task Weakly-Supervised Attention Network) |
| --- | --- |
| Publication Year | 2022 |
| Country / Setting | Multicentre; Alzheimer’s Disease Neuroimaging Initiative (ADNI-1 and ADNI-2) |
| Study Design | Deep-learning method development for joint regression of multiple dementia-related clinical scores from whole-brain structural MRI |
| Datasets Used | ADNI-1 and ADNI-2 baseline structural MRI with neuropsychological test scores |
| Recruitment Period | Baseline ADNI-1 (1.5T) and ADNI-2 (3T) imaging waves |
| Inclusion Criteria | Baseline T1-weighted MRI available; diagnosis of NC, sMCI, pMCI, or AD; complete baseline MMSE, CDRSB, and ADAS-Cog scores |
| Exclusion Criteria | Subjects overlapping between ADNI-1 and ADNI-2 removed from ADNI-2; scans failing preprocessing or missing key clinical scores excluded |
| Sample Size | Total 1,396 subjects with baseline MRI: combined ADNI-1 and ADNI-2 after removing duplicates |
| Group Distribution | ADNI-1: 226 NC, 225 sMCI, 165 pMCI, 181 AD; ADNI-2: 185 NC, 234 sMCI, 37 pMCI, 143 AD |
| Participant Demographics | Older adults with varying dementia status; demographics include age, sex, education; groups differ in mean MMSE, CDRSB, and ADAS-Cog |
| Modalities Used | Whole-brain T1-weighted structural MRI; clinical scores MMSE, CDRSB, and ADAS-Cog |
| Acquisition Details | ADNI-1 1.5T and ADNI-2 3T T1 MRI acquired under ADNI standardized multi-centre protocols |
| Preprocessing | Standard pipeline: AC–PC alignment (MIPAV), N3 bias correction, skull stripping (BET), cerebellum removal via template warping, linear alignment to Colin27 (FLIRT), resampling to 1×1×1 mm³, cropping to 144×184×152 voxels |
| Model Architecture | MWAN composed of three cascaded blocks: backbone 3D FCN for feature maps, weakly supervised dementia attention block to localize subject-specific discriminative regions, and attention-aware multitask regression block predicting multiple clinical scores |
| Training & Validation | End-to-end training with hybrid loss combining localization loss from auxiliary category labels (NC/sMCI/pMCI/AD) and regression loss for MMSE, CDRSB, and ADAS-Cog; cross-validation across ADNI-1 and ADNI-2; comparisons with single-task and non-attention baselines |
| Outcomes | Primary: regression of MMSE, CDRSB, and ADAS-Cog at baseline; evaluation via correlation coefficient (CC) and root mean square error (RMSE) |
| Results | MWAN achieved higher CC and lower RMSE for all three clinical scores compared with conventional machine-learning and earlier deep-learning methods. Joint multitasks learning and the dementia attention block both improved regression performance, especially on the smaller and more imbalanced ADNI-2 cohort |
| Missing Data Handling | Analyses restricted to subjects with complete MRI and all three clinical scores; no explicit imputation of missing scores or imaging voxels reported |
| Risk of Bias / Limitations | Limited to ADNI research cohorts with relatively homogeneous demographics; model evaluated only on structural MRI at baseline; potential overfitting due to modest sample size; lack of external validation on non-ADNI datasets |
| Funding / Conflicts | Supported by NIH grants AG041721 and MH117943; authors did not report conflicts of interest |

68

| Study ID | Li et al., 2019 |
| --- | --- |
| Publication Year | 2019 |
| Country / Setting | Multicentre; Alzheimer’s Disease Neuroimaging Initiative (USA/Canada) and AIBL (Australia) |
| Study Design | Deep-learning feature extraction from hippocampal MRI combined with time-to-event (Cox) prognostic modelling for prediction of progression from MCI to AD dementia |
| Datasets Used | ADNI-1, ADNI-GO & ADNI-2, and AIBL baseline structural MRI with longitudinal clinical follow-up |
| Recruitment Period | ADNI-1, ADNI-GO&2, and AIBL baseline MRI acquired before April 05, 2017 (download date); ADNI-1 used for training, ADNI-GO&2 and AIBL for external validation |
| Inclusion Criteria | For prognosis: all MCI subjects with baseline T1 MRI and at least one clinical follow-up visit, including those reverting from MCI to normal; for feature learning: AD and NC subjects with baseline hippocampal MRI |
| Exclusion Criteria | Subjects without follow-up data; scans failing hippocampal segmentation or registration; overlapping subjects excluded when defining independent training and validation cohorts |
| Sample Size | Total 2,146 subjects (803 for training, 1,343 for validation); for prognostic testing: 439 ADNI-GO&2 MCI subjects and 40 AIBL MCI subjects |
| Group Distribution | Training (ADNI-1) included NC, MCI, and AD for deep feature learning; prognostic evaluation focused on MCI subjects only in ADNI-GO&2 and AIBL |
| Participant Demographics | Middle-aged to older adults; demographic variables include age, sex, education, APOE4 status; cognitive measures available for MCI subjects (MMSE, ADAS-Cog13, RAVLT, FAQ) |
| Modalities Used | T1-weighted structural MRI focused on bilateral hippocampi; clinical and cognitive measures; CSF Aβ42 or AV-45 PET for amyloid status when available |
| Acquisition Details | ADNI-1 1.5T T1 MRI, ADNI-GO&2 3T T1 MRI, and AIBL 3T T1 MRI acquired under each study’s standard protocol |
| Preprocessing | Affine registration of T1 MRI to MNI space with 1×1×1 mm³ resolution; automated bilateral hippocampal segmentation using local label learning with 100 atlas templates; extraction of 3D hippocampal patches in a 29×21×55 voxel bounding box |
| Model Architecture | Two-stream 3D CNN with residual blocks and global average pooling applied separately to left and right hippocampal patches; concatenated 256-dimensional deep imaging features fed into a LASSO-regularized Cox proportional hazards model; a second Cox model combines deep-learning risk scores with baseline clinical variables |
| Training & Validation | Deep CNN trained on AD vs NC classification using ADNI-1; extracted deep imaging features used to train Cox time-to-event models on ADNI-1 MCI; external validation on ADNI-GO&2 and AIBL MCI cohorts; performance evaluated with concordance index and time-dependent ROC curves |
| Outcomes | Primary: time-to-event prediction of progression from MCI to AD dementia; Secondary: stratification of MCI subjects into risk groups and comparison with models using conventional hippocampal shape/texture features or clinical-only models |
| Results | Deep-learning imaging features achieved C-index 0.762 for ADNI-GO&2 and 0.781 for AIBL, outperforming models based on hippocampal shape and texture features. Among amyloid-positive MCI subjects, C-index was 0.733 vs 0.656–0.680 for conventional features. Time-dependent AUCs for ADNI-GO&2 were 0.75, 0.778, and 0.813 at years 1, 2, and 3. Combining deep-learning risk with clinical variables improved C-index to 0.864 (vs 0.848 for clinical-only models) and enabled significant risk-based stratification of MCI subjects (log-rank P < 0.0002). |
| Missing Data Handling | Subjects required at least one follow-up visit; amyloid status defined using CSF Aβ42 or AV-45 PET when available; analyses conducted on subjects with available imaging and clinical data; no detailed multiple imputation strategy reported |
| Risk of Bias / Limitations | Use of research cohorts (ADNI, AIBL) with limited representativeness; focus on hippocampal regions only, ignoring other brain areas; model trained on 1.5T and tested on 3T scans despite good feature reproducibility; potential cohort and scanner biases; no validation on non-ADNI/AIBL clinical populations |
| Funding / Conflicts | Data from ADNI and AIBL; study supported by institutional and grant funding (e.g., University of Pennsylvania); authors reported industry consulting for one co-author but no conflicts affecting this analysis |

9

| Study ID | Qiu et al., 2022 |
| --- | --- |
| Publication Year | 2022 |
| Country / Setting | United States; multi-centre dementia cohorts (NACC, ADNI, ADCP, etc.) |
| Study Design | Multicohort retrospective study developing and validating multimodal deep learning models for dementia status classification |
| Datasets Used | National Alzheimer’s Coordinating Centre (NACC); Alzheimer’s Disease Neuroimaging Initiative (ADNI); Alzheimer’s Disease Centres and other dementia cohorts with MRI and clinical data |
| Recruitment Period | Varies by cohort (primarily mid-2000s to late 2010s) as defined by each contributing study |
| Inclusion Criteria | Adults with normal cognition, mild cognitive impairment, Alzheimer’s disease dementia, or non-AD dementias with available structural MRI and clinical/neuropsychological assessments |
| Exclusion Criteria | Subjects without usable MRI; missing key diagnostic or cognitive information; severe imaging artifacts |
| Sample Size | Several thousand participants across all cohorts; exact numbers reported per cohort for NC, MCI, AD, and non-AD dementia groups |
| Group Distribution | Four main groups: normal cognition (NC), mild cognitive impairment (MCI), Alzheimer’s disease dementia (AD), and non-AD dementias (nADD) |
| Participant Demographics | Older adults (typically >55 years), mixed sex; predominantly North American academic memory clinic populations |
| Modalities Used | Structural T1-weighted MRI; demographics; medical history; functional and neuropsychological test scores |
| Acquisition Details | Standard clinical or research MRI protocols per cohort; images harmonized via preprocessing pipelines |
| Preprocessing | Standardization of MRI orientation and resolution; brain extraction and registration; intensity normalization; tabular variable cleaning and encoding |
| Model Architecture | Multimodal deep learning framework combining 3D convolutional neural networks for MRI with fully connected networks for clinical variables; flexible model variants for different modality combinations |
| Training & Validation | Training on NACC and related cohorts with cross-validation; external validation on ADNI and other independent cohorts; comparison with neurologist and neuroradiologist diagnoses |
| Outcomes | Primary: multi-class classification of NC, MCI, AD, and nADD; Secondary: model interpretability via saliency and SHAP-like methods and comparison to expert clinical performance |
| Results | Multimodal models achieved diagnostic performance comparable to practicing neurologists and neuroradiologists for distinguishing NC, MCI, AD, and nADD; imaging saliency maps and feature attributions aligned with known patterns of neurodegeneration and pathology at autopsy |
| Missing Data Handling | Use of flexible models that accept varying modality subsets; exclusion of subjects with insufficient core data; standard handling of missing tabular features |
| Risk of Bias / Limitations | Retrospective multi-cohort design; heterogeneity in recruitment and imaging protocols; limited generalizability beyond specialty memory centres; potential selection bias in research cohorts |
| Funding / Conflicts | Supported by NIH and institutional grants; authors reported standard disclosures, with no conflicts preventing publication |

69

| Study ID | Oh et al., 2023 (LEAR framework) |
| --- | --- |
| Publication Year | 2023 |
| Country / Setting | South Korea; ADNI structural MRI cohort |
| Study Design | Methodological study proposing a unified learn–explain–reinforce framework for Alzheimer’s disease diagnosis from structural MRI |
| Datasets Used | Alzheimer’s Disease Neuroimaging Initiative (ADNI) structural T1 MRI |
| Recruitment Period | ADNI baseline imaging waves (ADNI-1/GO/2) |
| Inclusion Criteria | Participants with baseline T1-weighted MRI and clinical diagnosis of cognitively normal (CN), mild cognitive impairment (MCI), or Alzheimer’s disease (AD) |
| Exclusion Criteria | Missing MRI data or poor-quality scans; incomplete diagnostic information |
| Sample Size | Several hundred ADNI participants across CN, MCI, and AD groups (exact counts reported in the original paper) |
| Group Distribution | Three main groups: CN, MCI, and AD used for model training and evaluation |
| Participant Demographics | Older adults from North American ADNI sites; mixed sex; typical ADNI demographics |
| Modalities Used | Whole-brain structural T1-weighted MRI |
| Acquisition Details | Standard ADNI 1.5T and 3T T1-weighted MRI protocols |
| Preprocessing | Conventional preprocessing including registration to standard space, intensity normalization, and skull stripping as described in the paper |
| Model Architecture | LEAR framework integrating a base CNN diagnostic model, an explanation unit that generates counterfactual maps, and a reinforcement unit that uses explanation-guided attention to refine representations |
| Training & Validation | End-to-end training on ADNI with iterative alternation of explanation and reinforcement units; evaluation via cross-validation and comparison with baseline CNN and standard XAI methods |
| Outcomes | Alzheimer’s disease vs. non-AD classification performance; quality and fidelity of counterfactual explanations; robustness of the reinforced model |
| Results | LEAR improved diagnostic accuracy and generalization compared with standard CNNs and conventional explanation approaches; counterfactual maps localized plausible atrophy patterns associated with AD and guided more discriminative attention |
| Missing Data Handling | Subjects with missing or unusable MRI were excluded; no complex statistical imputation was reported |
| Risk of Bias / Limitations | Single-cohort ADNI evaluation; limited demographic diversity; reliance on structural MRI only; XAI evaluation still partly qualitative |
| Funding / Conflicts | Supported by Korean government ICT and AI grants; authors reported no conflicts of interest |

70

| Study ID | Lian et al., 2020 (H-FCN) |
| --- | --- |
| Publication Year | 2020 |
| Country / Setting | United States; ADNI-1 and ADNI-2 structural MRI |
| Study Design | Deep-learning method development and evaluation for joint atrophy localization and Alzheimer’s disease diagnosis using structural MRI |
| Datasets Used | ADNI-1 and ADNI-2 baseline T1-weighted MRI |
| Recruitment Period | ADNI-1 (1.5T) and ADNI-2 (3T) baseline imaging phases |
| Inclusion Criteria | Subjects with baseline structural T1 MRI and diagnosis of NC, MCI, or AD; availability of follow-up used to define MCI converters and non-converters in some analyses |
| Exclusion Criteria | Subjects shared between ADNI-1 and ADNI-2 removed from ADNI-2; scans failing preprocessing or registration steps excluded |
| Sample Size | On the order of 1,400 subjects across ADNI-1 and ADNI-2 combined |
| Group Distribution | NC, sMCI, pMCI, and AD groups included from both ADNI-1 and ADNI-2; exact group counts reported in the paper |
| Participant Demographics | Older adults enrolled in ADNI; mixed sex; typical ADNI age and education distributions |
| Modalities Used | Whole-brain T1-weighted structural MRI |
| Acquisition Details | Multi-center 1.5T and 3T ADNI T1-weighted MRI using standardized acquisition protocols |
| Preprocessing | Affine alignment to AC–PC, N3 bias correction, skull stripping, cerebellum removal, linear registration to Colin27 template, resampling to 1 mm isotropic, cropping to a standardized 3D volume |
| Model Architecture | Hierarchical Fully Convolutional Network (H-FCN) that first localizes discriminative atrophic patches and regions, then learns multi-scale features and fuses them hierarchically for AD diagnosis |
| Training & Validation | Training and evaluation on ADNI-1 with cross-validation; independent testing and generalization assessment on ADNI-2; comparison with voxel-, region-, and patch-based baselines |
| Outcomes | Classification of AD vs. NC and MCI vs. NC; localization of discriminative atrophy patterns at multiple spatial scales |
| Results | H-FCN improved diagnostic accuracy compared with conventional feature-based methods, while simultaneously providing interpretable atrophy maps highlighting AD-related regions |
| Missing Data Handling | Analyses restricted to subjects with usable pre-processed MRI; no formal imputation of missing imaging data described |
| Risk of Bias / Limitations | Use of ADNI research cohort with limited representativeness; only structural MRI considered; strong reliance on preprocessing and registration accuracy; absence of external non-ADNI validation |
| Funding / Conflicts | Supported by NIH and related imaging research grants; authors reported no conflicts that would affect the work |

71

| Study ID | Avsec et al., 2021 (Enformer) |
| --- | --- |
| Publication Year | 2021 |
| Country / Setting | International; human and mouse genomic reference datasets |
| Study Design | Methodological study developing a transformer-based deep learning model for gene expression and chromatin-state prediction from DNA sequence |
| Datasets Used | Genome-wide human and mouse epigenomic and transcriptomic tracks (CAGE, histone marks, TF ChIP-seq, DNase/ATAC-seq) |
| Recruitment Period | Not applicable (reference genomics datasets rather than human participant recruitment) |
| Inclusion Criteria | Genomic regions with available high-quality CAGE and epigenetic tracks in human and mouse |
| Exclusion Criteria | Low-quality or low-coverage genomic tracks; held-out chromosomes reserved for testing |
| Sample Size | Effectively thousands of genome-wide tracks across chromosomes; training on most of the human and mouse genomes, testing on held-out regions |
| Group Distribution | Not applicable (continuous genomic prediction task rather than subject groups) |
| Participant Demographics | Not applicable |
| Modalities Used | DNA sequence windows up to 200 kb; labels are epigenetic and transcriptional signal tracks |
| Acquisition Details | Publicly available high-throughput sequencing datasets (CAGE, ChIP-seq, DNase/ATAC-seq) for human and mouse tissues and cell types |
| Preprocessing | Encoding DNA sequence into one-hot representation; constructing long input windows; normalization of epigenomic tracks; splitting chromosomes into training, validation, and test sets |
| Model Architecture | Enformer: convolutional front-end followed by transformer layers with multi-head self-attention, predicting thousands of epigenomic and expression tracks in a multitask setting |
| Training & Validation | Multitask training on human and mouse genomes; evaluation on held-out chromosomes; comparison against Basenji2 and ExPecto; ablations replacing attention with dilated convolutions |
| Outcomes | Prediction accuracy for CAGE-based gene expression at transcription start sites and epigenetic marks; ability to predict enhancer–promoter interactions and variant effects |
| Results | Enformer substantially improved correlation between predicted and observed gene expression over previous models and better captured long-range regulatory effects and variant impact, approaching experimental-level reproducibility |
| Missing Data Handling | Handling of missing signal via masking and track-specific normalization; low-quality tracks excluded from modelling |
| Risk of Bias / Limitations | Trained primarily on available cell types and assays; limited direct clinical validation; predictions depend on completeness and quality of epigenomic datasets |
| Funding / Conflicts | Supported by DeepMind and Calico Life Sciences; authors’ affiliations include commercial research labs |

72

| Study ID | Yang et al., 2021 |
| --- | --- |
| Publication Year | 2021 |
| Country / Setting | United States; ADNI research cohort |
| Study Design | Deep-learning based multimodal progression modelling study to derive an Alzheimer’s disease prognostic signature |
| Datasets Used | Alzheimer’s Disease Neuroimaging Initiative (ADNI) baseline MRI and other baseline biomarkers |
| Recruitment Period | ADNI baseline recruitment waves spanning mid-2000s to early 2010s |
| Inclusion Criteria | ADNI participants with baseline structural MRI and relevant clinical and biomarker data; diagnosis of AD, MCI, or cognitively normal |
| Exclusion Criteria | Missing key baseline imaging or biomarker measures; insufficient follow-up for prognostic modelling |
| Sample Size | On the order of several hundred to a thousand ADNI participants (exact counts per diagnostic group in the paper) |
| Group Distribution | Baseline AD, MCI, and cognitively normal groups used for model development and evaluation |
| Participant Demographics | Older adults typical of ADNI cohorts, with mixed sex and education levels |
| Modalities Used | Baseline structural brain MRI; cognitive test scores; CSF or PET biomarkers and other baseline clinical features |
| Acquisition Details | Standard ADNI protocols for MRI and biomarker acquisition across multiple centres |
| Preprocessing | MRI preprocessing including registration and feature extraction via deep networks; standardization of clinical and biomarker variables |
| Model Architecture | Deep learning model using baseline MRI to extract imaging features, integrated with other biomarkers via a Super Learner ensemble framework |
| Training & Validation | Internal cross-validation within ADNI; models trained to classify diagnostic status and derive a prognostic multimodal signature |
| Outcomes | Diagnostic classification of AD/MCI/NC and derived prognostic index reflecting risk of progression |
| Results | Deep-learning-derived multimodal signature distinguished diagnostic groups and captured heterogeneity in progression risk, suggesting utility for clinical trial enrichment |
| Missing Data Handling | Subjects with missing key baseline modalities excluded; standard handling of remaining missing values as described in the article |
| Risk of Bias / Limitations | Restricted to ADNI research cohort; limited external validation; potential cohort and selection biases; modest sample sizes for some subgroups |
| Funding / Conflicts | Supported by AbbVie and academic collaborations; authors reported corporate affiliations and standard disclosures |

73

| Study ID | Lee et al., 2024 |
| --- | --- |
| Publication Year | 2024 |
| Country / Setting | United Kingdom and Singapore; research cohort plus real-world memory clinics |
| Study Design | Predictive prognostic modeling study building a robust interpretable AI-guided marker for early dementia prediction |
| Datasets Used | ADNI and related research cohorts for model training; independent multicentre real-world memory clinic data from UK and Singapore for validation |
| Recruitment Period | ADNI recruitment mid-2000s onward; memory clinic recruitment over subsequent years up to early 2020s |
| Inclusion Criteria | Patients at early disease stages (typically MCI) with structural MRI, cognitive tests, and longitudinal clinical follow-up |
| Exclusion Criteria | Missing key imaging or cognitive data; inadequate follow-up to determine progression; non-AD dementias excluded from some analyses |
| Sample Size | Hundreds of participants in the training research cohort and external memory clinic validation cohorts |
| Group Distribution | Stable MCI vs. MCI converters to AD; additional clinical groups for robustness checks |
| Participant Demographics | Middle-aged to older adults from research and clinical populations; mixed sex; varying comorbidities and demographics across sites |
| Modalities Used | Cognitive test scores; structural MRI-derived grey matter atrophy measures; routine clinical variables |
| Acquisition Details | Research MRI from ADNI under standardized protocols; clinical MRI from multiple memory clinics using routine protocols; cognitive testing according to local clinical standards |
| Preprocessing | MRI processed to extract regional grey matter atrophy; cognitive measures standardized; harmonization across research and clinical cohorts |
| Model Architecture | Predictive prognostic model (PPM) integrating cognitive and imaging features to produce an individualized prognostic index |
| Training & Validation | Training on research cohort; validation on independent real-world multicentre memory clinic data; evaluation via accuracy, AUC, sensitivity, specificity, and time-to-dementia analyses |
| Outcomes | Primary: prediction of whether MCI patients remain stable or progress to AD; PPM-derived prognostic index as an individualized multimodal marker |
| Results | PPM achieved accuracy ~81.7%, AUC ~0.84, sensitivity ~82%, specificity ~81% for predicting progression; PPM-derived marker showed higher hazard ratios for conversion to AD than grey matter atrophy, cognitive scores, or clinical diagnosis alone |
| Missing Data Handling | Use of routinely collected variables common across cohorts; subjects with missing key modalities excluded from specific analyses |
| Risk of Bias / Limitations | Heterogeneity in real-world clinical data; potential site and scanner effects; limited representation of non-AD dementias in some analyses |
| Funding / Conflicts | Funded by Wellcome Trust, Royal Society, Alzheimer’s Research UK, Alzheimer’s Drug Discovery Foundation Diagnostics Accelerator, and the Alan Turing Institute; authors reported no conflicts preventing publication |

74

| Study ID | Zhu et al., 2021 (DA-MIDL) |
| --- | --- |
| Publication Year | 2021 |
| Country / Setting | China and United Kingdom; ADNI and AIBL research cohorts |
| Study Design | Deep-learning method development and evaluation for early Alzheimer’s disease and MCI diagnosis from structural MRI using dual-attention multi-instance learning |
| Datasets Used | ADNI and AIBL baseline structural T1-weighted MRI |
| Recruitment Period | ADNI and AIBL baseline imaging waves |
| Inclusion Criteria | Subjects with baseline sMRI and diagnoses of NC, MCI, or AD |
| Exclusion Criteria | Poor-quality MRI; missing diagnostic information |
| Sample Size | 1,689 subjects from ADNI and AIBL combined |
| Group Distribution | NC, MCI, and AD groups from both cohorts; exact subgroup counts detailed in the article |
| Participant Demographics | Older adults typical of ADNI and AIBL cohorts; mixed sex and education levels |
| Modalities Used | Whole-brain structural MRI subdivided into local patches |
| Acquisition Details | Standardized 1.5T/3T T1 MRI acquisition protocols in ADNI and AIBL |
| Preprocessing | Partition of whole-brain sMRI into multiple patches; intensity normalization and registration; patch extraction for input to Patch-Nets |
| Model Architecture | Dual Attention Multi-Instance Deep Learning (DA-MIDL) with Patch-Nets incorporating spatial attention, attention-based multi-instance pooling, and an attention-aware global classifier |
| Training & Validation | Training and cross-validation on ADNI; external testing on AIBL; comparisons with voxel-, region-, and patch-based baselines and other deep-learning methods |
| Outcomes | Classification of AD vs. NC and MCI vs. NC; identification of discriminative pathological locations |
| Results | DA-MIDL achieved higher accuracy and better generalizability than competing methods, and attention maps highlighted AD-related regions consistent with known pathology |
| Missing Data Handling | Analyses restricted to subjects with usable MRI; no explicit imputation of imaging features reported |
| Risk of Bias / Limitations | Use of research cohorts with limited demographic variety; reliance on structural MRI; potential dataset-specific overfitting |
| Funding / Conflicts | Supported by National Natural Science Foundation of China and related national programs; authors reported no conflicts impacting the study |

75

| Study ID | Zhang et al., 2024 |
| --- | --- |
| Publication Year | 2024 |
| Country / Setting | China; ADNI multimodal data |
| Study Design | Development of a feature-aware multimodal framework with automatic fusion for Alzheimer’s disease diagnosis |
| Datasets Used | Two ADNI datasets with multimodal AD diagnostic data (cognitive tests, MRI/PET, risk factors) |
| Recruitment Period | ADNI baseline recruitment periods for the two datasets |
| Inclusion Criteria | ADNI participants with available multimodal data including cognitive tests, neuroimaging, and risk-factor information |
| Exclusion Criteria | Missing key modalities or high levels of missing features; poor-quality imaging |
| Sample Size | Two cohorts achieving AD diagnosis accuracies of 95.9% and 91.9% respectively; sample sizes per cohort reported in the article |
| Group Distribution | AD, MCI, and cognitively normal groups across the two ADNI datasets |
| Participant Demographics | Older adults with suspected or established cognitive impairment; mixed sex; typical ADNI demographics |
| Modalities Used | Cognitive test scores; MRI/PET imaging-derived features; blood tests and risk factors |
| Acquisition Details | Standard ADNI acquisition protocols for MRI and PET; cognitive and laboratory assessments at baseline |
| Preprocessing | Low-dimensional SHAP-boosting feature selection; construction of low- and high-dimensional feature representations; normalization and encoding of heterogeneous multimodal inputs |
| Model Architecture | Feature-aware multimodal framework combining SHAP-based feature selection, cross-modal attention mechanisms, graph convolutional networks (GCN), and an automatic model fusion strategy (AMFS) |
| Training & Validation | Training and evaluation on two ADNI multimodal cohorts; AMFS learns optimal weights for submodels; performance compared with conventional multimodal fusion approaches |
| Outcomes | Primary: accurate AD vs. non-AD diagnosis; Secondary: efficient multimodal feature selection and interpretable fusion weights |
| Results | Framework achieved high AD diagnosis accuracies of 95.9% and 91.9% on two ADNI datasets, efficiently selecting clinically important features and improving fusion robustness |
| Missing Data Handling | Boosting feature selection and preprocessing routines used to mitigate missing features; subjects with excessive missingness excluded |
| Risk of Bias / Limitations | Reliance on ADNI research data; complex model may be challenging to deploy; need for external validation in real-world clinical settings |
| Funding / Conflicts | Funded by Science Innovation Programs led by Academicians in Chongqing and Chongqing Education Commission; authors reported no conflicts of interest |

76

| Study ID | Velazquez & Lee, 2022 |
| --- | --- |
| Publication Year | 2022 |
| Country / Setting | United States; ADNI Early Mild Cognitive Impairment (EMCI) cohort |
| Study Design | Multimodal ensemble modeling study predicting conversion from Early Mild Cognitive Impairment to Alzheimer’s disease |
| Datasets Used | ADNI EMCI diffusion tensor imaging (DTI) and electronic health records (EHR) |
| Recruitment Period | ADNI EMCI recruitment waves up to 2022 |
| Inclusion Criteria | EMCI subjects with baseline DTI scans and EHR-based biometric and neuropsychiatric test data |
| Exclusion Criteria | Lack of follow-up to determine conversion; missing DTI or EHR features required for modeling |
| Sample Size | 383 EMCI patients, including 49 converters (EMCI_C) and 335 non-converters (EMCI_NC) for EHR-based modeling; 405 DTI images from 90 distinct patients for CNN modeling |
| Group Distribution | EMCI converters to AD (EMCI_C) vs. non-converters (EMCI_NC) |
| Participant Demographics | Middle-aged to older adults diagnosed with EMCI; standard ADNI demographic mix |
| Modalities Used | Electronic health record features (biometrics, neuropsychiatric test scores) and DTI scans (ADC maps) |
| Acquisition Details | DTI acquired under ADNI diffusion imaging protocols; EHR features extracted from ADNI clinical database |
| Preprocessing | Oversampling of minority converter class for EHR data; data augmentation for DTI images; normalization of clinical features; preparation of ADC DTI images for CNN input |
| Model Architecture | Ensemble of a balanced random forest classifier for EHR features and a convolutional neural network (CNN) classifier for DTI scans; grid search used to determine optimal weighting between the two models |
| Training & Validation | Random forest trained on 288 patients with 95 held out for testing; CNN trained on 405 DTI images; ensemble weighting tuned via grid search; evaluation on held-out EMCI subjects |
| Outcomes | Primary: individualized prediction of EMCI to AD conversion |
| Results | Ensemble model achieved 98.81% accuracy for EMCI to AD conversion prediction and provided explainability via feature importance at both model and individual prediction levels |
| Missing Data Handling | Oversampling used to address class imbalance; subjects with missing critical features were excluded from training/testing sets |
| Risk of Bias / Limitations | Single-cohort ADNI EMCI population; small number of converters; very high reported accuracy may overestimate performance; limited external validation |
| Funding / Conflicts | No specific external funding reported beyond institutional support; authors did not report conflicts of interest |

77

| Study ID | Zhang et al., 2024 |
| --- | --- |
| Publication Year | 2024 |
| Country / Setting | China; ADNI research cohort |
| Study Design | Method development and evaluation study proposing a multimodal learning machine framework for AD diagnosis based on neuropsychological and neuroimaging data |
| Datasets Used | Alzheimer’s Disease Neuroimaging Initiative (ADNI) |
| Recruitment Period | ADNI baseline recruitment waves |
| Inclusion Criteria | Participants from ADNI with structural MRI and neuropsychological test data and clinical labels of NC, MCI, or AD |
| Exclusion Criteria | Subjects without required MRI or neuropsychological assessments; scans or records of insufficient quality |
| Sample Size | Subset of ADNI participants with both MRI and neuropsychological tests; exact group counts reported in the article |
| Group Distribution | Cognitively normal, MCI, and AD groups used for multimodal model development and testing |
| Participant Demographics | Older adults with suspected or established cognitive impairment; mixed sex; typical ADNI age and education distribution |
| Modalities Used | Structural MRI-derived embedding features; detailed multimodal neuropsychological tests organized by cognitive domains |
| Acquisition Details | Standard ADNI T1-weighted MRI protocols; neuropsychological assessments administered according to ADNI procedures |
| Preprocessing | Entropy-based polynomial dimension expansion of neuropsychological tests; construction of cognitive Extreme Learning Machine (ELM) models; normalization and embedding of MRI features |
| Model Architecture | Multimodal learning machine framework combining cognitive-domain ELM classifiers with neuroimaging-based models, fused via a boosting ensemble with optimized weights |
| Training & Validation | Training and testing on ADNI with cross-validation; ensemble weights automatically tuned to maximise diagnostic accuracy and F1 score |
| Outcomes | Primary: multi-class diagnosis of NC, MCI, and AD; Secondary: domain-specific cognitive ELM models and contribution of different modalities |
| Results | Proposed framework achieved over 98% accuracy and F1 scores on ADNI, with no observed bias between MCI and AD groups and improved use of rich neuropsychological test information |
| Missing Data Handling | Analyses conducted on subjects with available multimodal data; samples with missing critical modalities excluded; no complex imputation reported |
| Risk of Bias / Limitations | Single research cohort (ADNI); very high reported accuracy may overestimate performance; need for external clinical validation and assessment of generalizability |
| Funding / Conflicts | Supported by Chinese national and regional research grants; authors reported no conflicts of interest |

78

| Study ID | Bi et al., 2020 |
| --- | --- |
| Publication Year | 2020 |
| Country / Setting | China; ADNI multimodal imaging-genetics data |
| Study Design | Multimodal data analysis study using clustering evolutionary random forest to fuse fMRI and SNP data for AD diagnosis |
| Datasets Used | ADNI functional MRI (fMRI) and single nucleotide polymorphism (SNP) data |
| Recruitment Period | ADNI imaging-genetics recruitment period up to 2020 |
| Inclusion Criteria | ADNI subjects with both resting-state fMRI and SNP genotyping data and diagnostic labels |
| Exclusion Criteria | Subjects missing either fMRI or SNP data; poor-quality fMRI scans; incomplete genetic records |
| Sample Size | Subsample of ADNI participants with paired fMRI and SNP; exact numbers reported in the article |
| Group Distribution | AD patients vs. cognitively normal controls; possibly including MCI depending on analysis design |
| Participant Demographics | Older adults enrolled in ADNI; mixed sex; typical ADNI demographics |
| Modalities Used | Resting-state fMRI regional time series; SNP genotypes; derived brain region–gene pair fusion features |
| Acquisition Details | Standard ADNI resting-state fMRI protocols; genome-wide SNP genotyping using ADNI platforms |
| Preprocessing | Correlation analysis to link ROIs to genes; construction of brain region–gene pairs as multimodal features; normalization and dimensionality reduction as needed |
| Model Architecture | Cluster Evolutionary Random Forest (CERF) with hierarchical clustering of decision trees and selection of diverse high-performing tree subsets |
| Training & Validation | Random forest ensembles built with clustering evolution; feature construction, feature selection, and sample classification integrated; performance evaluated by classification accuracy and biomarker identification |
| Outcomes | Primary: discrimination between AD and controls; Secondary: identification of important brain regions and genes associated with AD |
| Results | CERF-based framework effectively identified AD patients and discovered significant brain region–gene pairs related to AD, outperforming several competing methods in classification performance |
| Missing Data Handling | Subjects with insufficient multimodal data excluded; standard handling of missing SNPs and imaging features as per ADNI preprocessing |
| Risk of Bias / Limitations | Small multimodal sample size; single cohort; complex model may be sensitive to hyperparameters; external validation not reported |
| Funding / Conflicts | Supported by National Natural Science Foundation of China and Hunan provincial projects; authors reported no conflicts of interest |

79

| Study ID | Bi et al., 2022 |
| --- | --- |
| Publication Year | 2022 |
| Country / Setting | China; ADNI MCI imaging-genetics cohort |
| Study Design | Multimodal imaging-genetics study using weighted evolutionary random forest for pathogeny detection in mild cognitive impairment |
| Datasets Used | ADNI functional MRI (fMRI) and SNP data for MCI patients |
| Recruitment Period | ADNI MCI recruitment up to 2022 |
| Inclusion Criteria | MCI subjects from ADNI with both resting-state fMRI and SNP data available |
| Exclusion Criteria | Missing fMRI or SNP data; low-quality imaging; incomplete genetic information |
| Sample Size | ADNI MCI subsample with paired fMRI and SNP; exact counts described in the article |
| Group Distribution | MCI vs. cognitively normal controls for classification; EMCI vs. LMCI or subgroups considered for pathogeny analysis |
| Participant Demographics | Middle-aged and older adults diagnosed with MCI; typical ADNI demographics |
| Modalities Used | Resting-state fMRI time series from ROIs; SNP genotypes; derived fusion features |
| Acquisition Details | Standard ADNI fMRI acquisition; GWAS SNP genotyping platforms as in ADNI |
| Preprocessing | Construction of fusion features using correlation analysis between ROI sequences and digitalized gene sequences; feature construction followed by selection and classification steps |
| Model Architecture | Weighted Evolutionary Random Forest (WERF) introducing a weighted evolution strategy into ensemble learning to eliminate inefficient features and emphasize important ones |
| Training & Validation | Multimodal analysis framework built on WERF; performance compared with other popular methods on ADNI MCI dataset; evaluation focused on classification accuracy and identification of pathogenic factors |
| Outcomes | Primary: accurate identification of MCI patients; Secondary: extraction of pathogenic brain regions and genetic variants |
| Results | WERF-based framework achieved superior performance in identifying MCI patients compared with several state-of-the-art methods and highlighted key ROIs and SNPs potentially related to MCI pathogenesis |
| Missing Data Handling | Subjects with incomplete imaging or genetic data excluded; standard SNP and fMRI preprocessing applied; no explicit multiple imputation described |
| Risk of Bias / Limitations | Single-cohort imaging-genetics sample; potential overfitting due to high-dimensional fusion features and small N; limited external generalizability |
| Funding / Conflicts | Supported by National Natural Science Foundation of China and multiple provincial and educational grants; authors reported no conflicts of interest |

80

| Study ID | Hashmi & Barukab, 2023 |
| --- | --- |
| Publication Year | 2023 |
| Country / Setting | Saudi Arabia; open OASIS MRI dataset |
| Study Design | Deep reinforcement learning and neural network-based method development for dementia classification from MRI |
| Datasets Used | Open Access Series of Imaging Studies (OASIS) MRI dataset |
| Recruitment Period | OASIS acquisition period as defined by the original dataset |
| Inclusion Criteria | OASIS participants with structural MRI and dementia labels across four stages |
| Exclusion Criteria | Images with inadequate quality; subjects without clear dementia staging information |
| Sample Size | Subset of OASIS subjects used for four-class dementia staging; exact numbers reported in the article |
| Group Distribution | Four dementia stages: nondemented, very mild, mild, and moderate-severe dementia |
| Participant Demographics | Adults and older adults from the OASIS cohort; mixed sex |
| Modalities Used | Structural MRI images; derived sickness probability maps |
| Acquisition Details | OASIS MRI acquisition protocols for structural brain imaging |
| Preprocessing | Class balancing via generative reinforcement learning approach; segmentation and preprocessing of MRI images prior to classification |
| Model Architecture | Deep reinforcement learning system combined with neural network classifier to generate and segment balanced class samples and produce dementia risk maps |
| Training & Validation | Training on imbalanced OASIS dataset with RL-driven augmentation to balance classes; evaluation of accuracy, precision, recall, and F-score with and without the proposed approach |
| Outcomes | Primary: four-class dementia classification performance; Secondary: improvement in diagnostic efficiency after RL-based augmentation |
| Results | Deep reinforcement learning system improved accuracy by about 6%, precision by about 9%, recall by about 13%, and F-score by about 9–10% compared with baseline methods |
| Missing Data Handling | Focus on class imbalance rather than missing values; subjects with unusable images likely excluded |
| Risk of Bias / Limitations | Single open dataset; focus on MRI only; potential overfitting to OASIS characteristics; need for testing on clinical cohorts |
| Funding / Conflicts | Supported by King Abdulaziz University; authors declared no conflicts of interest |

102

| Study ID | Shi et al., 2018 |
| --- | --- |
| Publication Year | 2018 |
| Country / Setting | China; Alzheimer’s Disease Neuroimaging Initiative (ADNI) dataset |
| Study Design | Method development and evaluation study proposing multimodal stacked deep polynomial networks (MM-SDPN) for AD and MCI diagnosis using MRI and PET |
| Datasets Used | ADNI structural MRI and FDG-PET (or amyloid PET) from subjects with AD, MCI and cognitively normal controls |
| Recruitment Period | ADNI-1 and related phases; baseline scans used for classification tasks |
| Inclusion Criteria | ADNI participants with both T1-weighted MRI and PET scans usable at baseline and clinical diagnoses of AD, MCI or NC |
| Exclusion Criteria | Missing or poor-quality MRI or PET; incomplete clinical diagnosis; failure of preprocessing |
| Sample Size | ADNI subset with paired MRI and PET; exact sample counts for AD, MCI and NC given in the paper |
| Group Distribution | Binary tasks: AD vs NC, MCI vs NC; multiclass task: NC vs MCI vs AD |
| Participant Demographics | Older adults typical of ADNI; demographic variables (age, sex, MMSE, etc.) reported per group |
| Modalities Used | Structural MRI and PET neuroimaging (voxel/patch-level features) |
| Acquisition Details | Standard ADNI MRI and PET acquisition protocols across multiple sites |
| Preprocessing | Extraction of voxel or patch-based neuroimaging features; modality-specific feature scaling and preparation as inputs to initial SDPNs |
| Model Architecture | Multimodal stacked deep polynomial network (MM-SDPN) consisting of two stage SDPNs: first-level SDPNs learn high-level features from MRI and PET separately; a second SDPN fuses these high-level representations |
| Training & Validation | Supervised training of modality-specific SDPNs followed by fusion SDPN; comparison with state-of-the-art multimodal feature learning algorithms for AD diagnosis; evaluation on binary and multiclass classification tasks |
| Outcomes | Primary: improved diagnostic accuracy for AD and MCI classification compared with existing multimodal feature learning methods |
| Results | MM-SDPN outperformed competing multimodal approaches on ADNI across binary and multiclass tasks, demonstrating the effectiveness of deep polynomial fusion for small-sample neuroimaging data |
| Missing Data Handling | Only participants with usable MRI and PET were included; no explicit multiple imputation strategy described |
| Risk of Bias / Limitations | Restricted to ADNI research cohort; relatively small number of subjects with paired multimodal scans; computational complexity of stacked DPNs; need for external validation |
| Funding / Conflicts | Supported by the National Natural Science Foundation of China and Guangdong/Shenzhen research projects; authors reported no conflicts of interest |

81

| Study ID | Wang et al., 2024 |
| --- | --- |
| Publication Year | 2024 |
| Country / Setting | China; ADNI multicentre cohort |
| Study Design | Multicentre multimodal deep learning study predicting long-term progression from MCI to AD incorporating interaction effects |
| Datasets Used | Alzheimer’s Disease Neuroimaging Initiative (ADNI-1, ADNI-2/GO, ADNI-3) |
| Recruitment Period | Baseline ADNI-1 and ADNI-2/GO cohorts with follow-up; ADNI-3 used for independent generalization |
| Inclusion Criteria | Patients with MCI at baseline with available structural MRI, clinical assessments, and genetic polymorphism data and at least 4 years of follow-up |
| Exclusion Criteria | Subjects without required multimodal data; insufficient follow-up to determine conversion status |
| Sample Size | 252 MCI patients at baseline from ADNI used for model development and validation |
| Group Distribution | MCI converters to AD vs. non-converters defined within a 4-year horizon |
| Participant Demographics | Middle-aged and older MCI patients from ADNI; mixed sex; typical research memory clinic demographics |
| Modalities Used | Baseline structural MRI; clinical and neuropsychological assessments; genetic SNPs including APOE |
| Acquisition Details | Standard ADNI MRI and biomarker acquisition across multiple centres |
| Preprocessing | Extraction of imaging, clinical, and genetic features; feature scaling; construction of multimodal interaction terms within the deep learning model |
| Model Architecture | Interpretable multimodal deep learning model with a novel interaction layer capturing interactions among multimodal biomarkers |
| Training & Validation | Cross-validated on ADNI-1 and ADNI-2/GO; generalized in ADNI-3; performance evaluated with AUC, accuracy, sensitivity, specificity, and F1 score; ablation of interaction and multimodality effects |
| Outcomes | Primary: prediction of MCI-to-AD conversion within 4 years; Secondary: contribution of interaction effects and each modality to prediction |
| Results | Cross-validation AUC 0.962 and accuracy 92.92% for 4-year conversion prediction; independent test AUC 0.939 and accuracy 92.86%; incorporating interaction effects increased accuracy by 4.76% and multimodality by 4.29%; model robust to inter-centre and inter-scanner variability and provided interpretable biomarker contributions |
| Missing Data Handling | Analyses limited to subjects with complete multimodal data; robustness to missingness across centres assessed qualitatively; no advanced imputation described |
| Risk of Bias / Limitations | Single research cohort; modest sample size; potential overfitting despite cross-validation; need for validation in non-ADNI clinical populations |
| Funding / Conflicts | Supported by Chinese national research grants; authors reported no conflicts of interest |

82

| Study ID | Hatami et al., 2024 |
| --- | --- |
| Publication Year | 2024 |
| Country / Setting | Iran; ADNI MRI dataset |
| Study Design | Methodological study investigating reinforcement learning and deep learning for improving Alzheimer’s disease MRI classification via adaptive data augmentation |
| Datasets Used | Alzheimer’s Disease Neuroimaging Initiative (ADNI) structural MRI |
| Recruitment Period | ADNI recruitment up to the version used in 2024 |
| Inclusion Criteria | ADNI subjects with structural MRI suitable for AD vs. non-AD classification |
| Exclusion Criteria | Missing MRI scans; poor-quality images; incomplete labels |
| Sample Size | ADNI subset with AD and cognitively normal (and possibly MCI) subjects; exact counts reported in the article |
| Group Distribution | AD vs. healthy controls and related classification tasks depending on experimental setting |
| Participant Demographics | Older adults enrolled in ADNI; mixed sex |
| Modalities Used | Structural brain MRI; derived image representations processed by deep networks |
| Acquisition Details | Standard ADNI T1-weighted MRI protocols |
| Preprocessing | Preparation of MRI images for deep neural network input; application of RL-driven data augmentation policies to address class imbalance and data scarcity |
| Model Architecture | Pretrained deep neural network for image classification combined with a reinforcement learning agent that optimizes data augmentation strategies based on a secondary classifier’s feedback |
| Training & Validation | Initial classification with a pretrained DNN; concurrent training of a secondary classifier to provide reward signals; RL agent learns augmentation policies; evaluation via AUC, specificity, sensitivity, and accuracy compared with existing techniques |
| Outcomes | Primary: improved Alzheimer’s MRI classification performance; Secondary: demonstration that RL-guided augmentation reduces dataset dependence of augmentation strategies |
| Results | Proposed method achieved higher performance than several baseline approaches on ADNI, with reported precision around 0.95, specificity and sensitivity improvements, and increased AUC and accuracy |
| Missing Data Handling | Focus on augmentation rather than missing values; subjects with missing MRI or labels excluded from analysis |
| Risk of Bias / Limitations | Reliance on a single research cohort; no external clinical validation; results may depend on hyperparameter choices and network architecture |
| Funding / Conflicts | Conducted with institutional support from Iranian universities; authors did not report conflicts of interest |

83

| Study ID | Tabarestani et al., 2020 |
| --- | --- |
| Publication Year | 2020 |
| Country / Setting | United States; ADNI longitudinal multimodal cohort |
| Study Design | Longitudinal distributed multitask multimodal regression study predicting Alzheimer’s disease progression using multiple biomarkers |
| Datasets Used | Alzheimer’s Disease Neuroimaging Initiative (ADNI) |
| Recruitment Period | ADNI longitudinal follow-up spanning mid-2000s to late 2010s |
| Inclusion Criteria | ADNI subjects with longitudinal cognitive scores and at least one imaging or biomarker modality such as MRI, FDG-PET, CSF, EEG, and risk factors |
| Exclusion Criteria | Subjects without sufficient follow-up visits; missing all or most biomarker modalities |
| Sample Size | Longitudinal cohort of ADNI subjects with varying combinations of modalities; exact sample sizes per modality reported in the article |
| Group Distribution | Cognitively normal, MCI, and AD participants followed over time; tasks defined per time point rather than strict group labels |
| Participant Demographics | Older adults at risk for or diagnosed with AD; mixed sex; typical ADNI demographics |
| Modalities Used | Structural MRI, FDG-PET, EEG, CSF biomarkers, cognitive scores, demographics, and genetic risk factors |
| Acquisition Details | Multicentre ADNI acquisition protocols for MRI, PET, CSF, EEG, and clinical assessments |
| Preprocessing | Feature extraction for each modality (e.g., cortical thickness, regional volumes, PET hypometabolism, CSF concentrations); handling of modality-specific sparsity; normalization of features |
| Model Architecture | Distributed multitask multimodal learning framework with modality-specific multitask regression coefficient matrices and fused sparse group Lasso regularization; gradient boosting on learned risk factor parameters |
| Training & Validation | Each task predicts cognitive scores at a given time; multitask regression links chronological tasks; outputs from modality-specific learners combined and fed to gradient boosting; performance compared with multiple baseline models |
| Outcomes | Primary: prediction of longitudinal cognitive scores and AD progression; Secondary: identification of prevalent multimodal trends across modalities |
| Results | Proposed approach reduced prediction errors compared with established unimodal and multimodal methods, particularly when data were incomplete and sparsely sampled over time |
| Missing Data Handling | Explicit modelling of missing modalities via separate multitask coefficient matrices; framework robust to incomplete longitudinal data |
| Risk of Bias / Limitations | Single research cohort; complexity of model may limit clinical deployment; potential sensitivity to hyperparameters and modality imbalance |
| Funding / Conflicts | Supported by US research grants and institutional funding; authors reported no conflicts of interest |

84

| Study ID | Burkhart et al., 2024 |
| --- | --- |
| Publication Year | 2024 |
| Country / Setting | United Kingdom and Singapore; research cohort plus real-world memory clinic data |
| Study Design | Unsupervised multimodal trajectory modelling study for early dementia prediction using longitudinal biomarker and cognitive data |
| Datasets Used | Alzheimer’s Disease Neuroimaging Initiative (ADNI) research cohort and memory clinic patients from Singapore |
| Recruitment Period | ADNI longitudinal recruitment; memory clinic data collected in recent years up to 2024 |
| Inclusion Criteria | Individuals with longitudinal cognitive assessments and imaging biomarkers (β-amyloid, grey matter density) in ADNI; memory clinic patients with varying data completeness |
| Exclusion Criteria | Subjects without sufficient longitudinal data for trajectory modelling; missing all key biomarkers and cognitive assessments |
| Sample Size | ADNI training and test sets plus an independent real-world memory clinic sample; exact numbers detailed in the article |
| Group Distribution | Cognitively normal, MCI, and dementia patients included, but modelling is unsupervised and does not rely on diagnostic labels for training |
| Participant Demographics | Older adults at risk of or with cognitive impairment; mixed sex; ADNI North American research cohort and Asian memory clinic populations |
| Modalities Used | Longitudinal cognitive test scores; β-amyloid PET; grey matter density from structural MRI |
| Acquisition Details | ADNI PET and MRI acquisition according to standard protocols; clinical MRI and assessments in memory clinics following local practice |
| Preprocessing | Construction of longitudinal trajectories of biomarkers and cognition; state-space model representation; handling of missing observations |
| Model Architecture | Multimodal trajectory modelling (MTM) approach based on a mixture of state space models linking latent biomarker states to observed cognitive performance |
| Training & Validation | Unsupervised training on ADNI longitudinal trajectories; evaluation of clustering quality and ability to stratify individuals into cognitive health clusters; external validation on memory clinic data |
| Outcomes | Derivation of individualized cognitive health indices and clusters; prediction of progression to AD compared with standard clinical assessments |
| Results | MTM training on trajectories stratified individuals into clinically meaningful clusters more reliably than baseline approaches; MTM-derived indices predicted progression to AD more precisely than cognitive tests or MRI alone and generalized to real-world memory clinic data with missing values |
| Missing Data Handling | Model explicitly designed to be robust to missing data, including cognitive-only trajectories and single assessments; uses learning with privileged information for imaging biomarkers |
| Risk of Bias / Limitations | Complex unsupervised framework may be challenging to deploy; reliance on ADNI for training; memory clinic sample size and heterogeneity may limit generalizability estimates |
| Funding / Conflicts | Supported by UK and Singaporean research funding bodies; authors reported no conflicts impacting the study |

85

| Study ID | El-Sappagh et al., 2021 |
| --- | --- |
| Publication Year | 2021 |
| Country / Setting | Spain, Egypt, Korea; ADNI multimodal dataset |
| Study Design | Multilayer multimodal detection and prediction model for AD based on explainable artificial intelligence, combining diagnosis and MCI progression detection |
| Datasets Used | Alzheimer’s Disease Neuroimaging Initiative (ADNI) dataset with 11 modalities |
| Recruitment Period | ADNI data collected over multiple phases up to the time of analysis |
| Inclusion Criteria | 1048 ADNI subjects with sufficient multimodal data, including 294 cognitively normal, 254 stable MCI, 232 progressive MCI, and 268 AD |
| Exclusion Criteria | Subjects lacking key modalities required by the model; incomplete follow-up for progression labelling |
| Sample Size | 1048 subjects (294 CN, 254 sMCI, 232 pMCI, 268 AD) |
| Group Distribution | First layer: multi-class classification CN, sMCI, pMCI, AD; Second layer: binary classification pMCI vs sMCI for progression detection |
| Participant Demographics | Older adults recruited into ADNI; mixed sex; typical research memory clinic demographics |
| Modalities Used | Eleven modalities including MRI, PET, CSF biomarkers, cognitive and functional assessments, neuropsychological tests, demographics, medical history, lab tests, and others |
| Acquisition Details | Multimodal data collected using standard ADNI protocols for imaging, fluid biomarkers, and clinical assessments |
| Preprocessing | Feature selection and optimization from a large multimodal feature set; recursive feature elimination; normalization and encoding of clinical variables |
| Model Architecture | Two-layer model using random forest as the main classifier; first layer for multi-class diagnosis, second layer for MCI progression detection; explainability via SHAP and additional decision-tree and fuzzy rule-based explainers |
| Training & Validation | Cross-validation within ADNI; optimization of RF performance and marker selection; evaluation via accuracy, F1-score, and AUC; explanation generation at global and instance levels |
| Outcomes | Primary: accurate AD multi-class diagnosis and prediction of MCI progression; Secondary: interpretable explanations for each decision and insight into modality contributions |
| Results | First layer achieved cross-validation accuracy of 93.95% and F1-score of 93.94%; second layer achieved accuracy of 87.08% and F1-score of 87.09%; explanations broadly consistent with each other and with AD medical literature |
| Missing Data Handling | Subjects with missing key modalities excluded; multimodal feature selection reduced redundancy and noise; no complex imputation scheme detailed |
| Risk of Bias / Limitations | Single research cohort; high model complexity; potential challenges for deployment in routine care despite strong explainability; absence of external validation outside ADNI |
| Funding / Conflicts | Supported by European and Korean research programs and institutional funding; authors reported no conflicts of interest |

86

| Study ID | Lee et al., 2024 |
| --- | --- |
| Publication Year | 2024 |
| Country / Setting | Republic of Korea and USA; four Korean hospitals plus ADNI cohort |
| Study Design | Multicentre multimodal machine learning study predicting dementia conversion among MCI patients |
| Datasets Used | Four Korean memory clinics and the Alzheimer’s Disease Neuroimaging Initiative (ADNI) |
| Recruitment Period | Patients with MCI enrolled with up to four years of clinical follow-up |
| Inclusion Criteria | MCI diagnosis at baseline, available T1 and T2-FLAIR MRI and amyloid PET, demographic data, and longitudinal follow-up to determine conversion within 4 years |
| Exclusion Criteria | Major neurological or psychiatric comorbidities, insufficient imaging quality, or lack of longitudinal data to define conversion status |
| Sample Size | 196 MCI subjects in total |
| Group Distribution | Stable MCI: 149 (76%); MCI-to-AD converters: 47 (24%) |
| Participant Demographics | Older adults with baseline MCI; age, sex, education, and cognitive scores reported per site; mixture of clinic-based Korean patients and ADNI research participants |
| Modalities Used | Structural MRI (T1-weighted), T2-FLAIR MRI, amyloid PET (SUVR), hippocampal occupancy scores (HOC), Fazekas scale, and demographic/cognitive variables |
| Acquisition Details | Routine clinical MRI (T1 and T2-FLAIR) and amyloid PET acquired at four Korean centres; ADNI imaging acquired under standardized multicentre research protocols |
| Preprocessing | Automatic brain segmentation; calculation of regional volumes, regional amyloid SUVR, white matter hyperintensity burden and HOC; feature scaling and combination into modality-specific and multimodal feature sets |
| Model Architecture | Conventional machine learning classifiers including gradient boosting machine (GBM), random forest, support vector machine, logistic regression, k-nearest neighbours and naïve Bayes |
| Training & Validation | Nested cross-validation to compare six ML models under different modality combinations; assessment of robustness and performance when including or excluding specific imaging modalities (e.g., T2-FLAIR) |
| Outcomes | Primary: prediction of conversion from MCI to AD within 4 years; Secondary: identification of most informative modality combinations for robust prediction |
| Results | GBM provided the most stable performance across modality combinations; models using T1 MRI and amyloid PET features achieved the best prediction, and performance improved when T2-FLAIR features were omitted from the multimodal set |
| Missing Data Handling | Subjects with missing key imaging modalities were excluded from specific analyses; feature sets constructed from available modalities per subject |
| Risk of Bias / Limitations | Relatively small multicentre sample; mix of hospital-based and research cohorts; potential scanner and site heterogeneity; limited external validation beyond participating centres and ADNI subset |
| Funding / Conflicts | Supported by Korean national research grants and institutional funding; authors reported no conflicts of interest |

87

88

| Study ID | Yuan et al., 2021 |
| --- | --- |
| Publication Year | 2021 |
| Country / Setting | China; ADNI research cohorts |
| Study Design | Multimodal co-training study using both labelled and unlabelled samples to classify MCI subtypes |
| Datasets Used | Alzheimer’s Disease Neuroimaging Initiative (ADNI-1 for training, ADNI-2 for independent testing) |
| Recruitment Period | ADNI-1 and ADNI-2 baseline imaging-genetics waves |
| Inclusion Criteria | MCI subjects from ADNI-1 with structural MRI and genotype data; additional MCI subjects from ADNI-2 for external validation |
| Exclusion Criteria | Missing sMRI or SNP data; insufficient quality of imaging or genotyping; lack of follow-up to determine sMCI or pMCI status among labelled subjects |
| Sample Size | 364 MCI subjects from ADNI-1 (228 labelled and 136 unlabeled) plus an independent ADNI-2 MCI test set |
| Group Distribution | ADNI-1 labelled: stable MCI (sMCI) vs progressive MCI (pMCI); ADNI-1 unlabelled: MCI with unknown progression; ADNI-2: independent MCI cohort for testing |
| Participant Demographics | Older adults with MCI; mixed sex; typical ADNI memory-clinic research population |
| Modalities Used | Structural MRI quantitative trait (QT) features and SNP genotype features |
| Acquisition Details | T1-weighted structural MRI and genome-wide SNP genotyping acquired using standard ADNI protocols |
| Preprocessing | Extraction of QT features from sMRI; SNP selection; normalization of imaging and genetic features; construction of two initial classifiers using sMRI and SNPs on labelled samples |
| Model Architecture | Multimodal co-training framework with two base classifiers (one on QT features and one on SNP features) iteratively labeling unlabeled MCI samples; final random forest classifier trained on combined labeled data |
| Training & Validation | Initial classifiers trained on 228 labelled ADNI-1 MCI samples; co-training iteratively labelled 136 unlabelled samples; final model evaluated on independent ADNI-2 MCI cohort |
| Outcomes | Primary: classification of MCI patients into sMCI vs pMCI; Secondary: assessment of complementarity between sMRI-derived QT features and SNP data |
| Results | Final multimodal co-training framework achieved 85.50% accuracy and AUC 0.825 on the ADNI-2 test set for MCI classification, outperforming single-modality and non-co-training baselines |
| Missing Data Handling | Only subjects with both usable sMRI and SNP data were included; no complex imputation strategy reported |
| Risk of Bias / Limitations | Restricted to ADNI research cohorts; modest sample size; dependence on feature selection and co-training iterations; external validation beyond ADNI was not performed |
| Funding / Conflicts | Supported by Chinese national and provincial research grants; authors reported no conflicts of interest |

89

| Study ID | Cirincione et al., 2024 |
| --- | --- |
| Publication Year | 2024 |
| Country / Setting | United States; TADPOLE/ADNI international research cohort |
| Study Design | Multimodal machine learning study predicting future dementia among MCI patients using Ensemble Integration (EI) |
| Datasets Used | Alzheimer’s Disease Prediction of Longitudinal Evolution (TADPOLE) challenge dataset, derived from ADNI |
| Recruitment Period | ADNI longitudinal recruitment underlying TADPOLE; MCI patients with baseline and follow-up data |
| Inclusion Criteria | Patients diagnosed with MCI at baseline with multimodal baseline data (clinical, cognitive, MRI, PET and other biomarkers) and longitudinal follow-up to determine dementia conversion |
| Exclusion Criteria | Missing essential clinical or imaging modalities; lack of follow-up labels for future dementia status |
| Sample Size | MCI subset of the TADPOLE dataset; exact numbers for training and test sets reported in the article |
| Group Distribution | Baseline MCI patients who progressed to dementia versus those who remained non-demented during follow-up |
| Participant Demographics | Older adults with MCI from ADNI; mixed sex; typical North American research memory-clinic population |
| Modalities Used | Multimodal clinical and imaging data including cognitive test scores, sMRI-derived neuroanatomical measurements, PET, and other biomarkers |
| Acquisition Details | Standard ADNI imaging and clinical data collection as organized in the TADPOLE challenge dataset |
| Preprocessing | Within-modality feature processing and normalization; construction of modality-specific models; integration of heterogeneous feature types within the Ensemble Integration framework |
| Model Architecture | Ensemble Integration (EI) framework that combines predictions from multiple modality-specific models to capture complementarity and consensus; comparisons with XGBoost and deep learning baselines |
| Training & Validation | Training on TADPOLE training subset; evaluation on held-out test set; model performance compared using AUC and F-measure against XGBoost and deep neural network baselines |
| Outcomes | Primary: prediction of future dementia development among MCI patients; Secondary: identification of important MRI regions and other features associated with progression |
| Results | EI-based model achieved AUC 0.81 and F-measure 0.68 on the held-out test set, outperforming XGBoost (AUC 0.68, F 0.57) and a deep learning baseline (AUC 0.79, F 0.61); MRI volumes of middle temporal gyrus, posterior cingulate gyrus, and inferior lateral ventricles were highlighted as predictive features |
| Missing Data Handling | Ensemble Integration designed to work with heterogeneous multimodal data; samples with missing key features for core modalities were excluded from analysis |
| Risk of Bias / Limitations | Dependence on a single research dataset (TADPOLE/ADNI); potential limitations in generalizing to routine clinical populations; complexity of multimodal integration may hinder straightforward clinical deployment |
| Funding / Conflicts | Supported by institutional and grant funding at the Icahn School of Medicine at Mount Sinai; authors reported no conflicts of interest |

90

| Study ID | Cassani & Falk, 2020 |
| --- | --- |
| Publication Year | 2020 |
| Country / Setting | Canada; resting-state EEG recordings from AD patients and normal elderly controls |
| Study Design | EEG-based biomarker development for AD diagnosis and severity level detection using modulation spectral patch features |
| Datasets Used | Clinical EEG recordings from Alzheimer’s disease patients with varying severity levels and healthy normal elderly controls |
| Recruitment Period | EEG acquired during routine or research resting-state exams over several years (exact period reported in the article) |
| Inclusion Criteria | Older adults diagnosed with mild or moderate AD and age-matched healthy normal elderly controls with artifact-free resting-state EEG |
| Exclusion Criteria | Other major neurological or psychiatric disorders; poor-quality EEG; excessive artifacts |
| Sample Size | Moderate-sized cohort of AD patients and controls; exact counts per group detailed in the article |
| Group Distribution | Normal elderly controls (Nold), mild AD, and moderate AD patient groups |
| Participant Demographics | Elderly subjects; demographic characteristics (age, sex, MMSE, CDR) summarized per group |
| Modalities Used | Resting-state EEG (rsEEG) signals |
| Acquisition Details | Standard multi-channel scalp EEG recorded at rest with eyes closed; sampling rates and montage specified in the study |
| Preprocessing | Artifact rejection; bandpass filtering; computation of 2D modulation spectrograms; extraction of amplitude modulation features across carrier and modulation frequency axes |
| Model Architecture | Feature-engineering approach using power in predefined modulation spectral "patches" as features; classification with conventional machine learning classifiers |
| Training & Validation | Cross-validation experiments comparing proposed modulation patch features to traditional EEG spectral features for Nold vs AD and mild vs moderate AD classification |
| Outcomes | Primary: discrimination between Nold and AD patients; Secondary: discrimination between mild and moderate AD severity levels |
| Results | Modulation spectral patch features outperformed traditional rsEEG features, achieving higher accuracy in both AD vs Nold discrimination and severity level classification, and offering biologically plausible neuromodulatory biomarkers |
| Missing Data Handling | EEG segments with excessive artifacts rejected; analyses focused on subjects with sufficient clean data; no statistical imputation described |
| Risk of Bias / Limitations | Single-centre EEG dataset; relatively small sample size; resting-state only; need for external validation and comparison to imaging biomarkers |
| Funding / Conflicts | Supported by the Natural Sciences and Engineering Research Council of Canada; authors reported no conflicts of interest |

95

| Study ID | Cilia et al., 2021 |
| --- | --- |
| Publication Year | 2021 |
| Country / Setting | Italy; hospital-based cohort from Naples |
| Study Design | Decision-support system for early AD detection based on online handwriting converted into synthetic images and analyzed via deep transfer learning |
| Datasets Used | Custom database of online handwriting samples collected with a graphic tablet from AD patients and healthy controls |
| Recruitment Period | Participants recruited through the geriatric Alzheimer unit of the “Federico II” hospital in Naples |
| Inclusion Criteria | Subjects with an initial level of AD and cognitively healthy controls able to complete standardized handwriting and drawing tasks on a graphic tablet |
| Exclusion Criteria | Severe visual or motor impairment preventing handwriting; other major neurological or psychiatric disorders; incomplete task performance |
| Sample Size | Approximately 180 subjects including AD patients and controls (exact group sizes specified in the article) |
| Group Distribution | Two main groups: early AD patients and age-matched healthy control subjects |
| Participant Demographics | Older adults; detailed demographic and cognitive information summarized per group |
| Modalities Used | Online handwriting signals (pen trajectory, pressure, speed, acceleration) transformed into offline synthetic images encoding dynamic information in RGB channels; binary images capturing shape only |
| Acquisition Details | Handwriting and drawing tasks administered on a digitizing tablet, capturing time-stamped x–y coordinates and pen pressure |
| Preprocessing | Generation of synthetic color images where each stroke’s color encodes dynamic information; generation of binary images containing only shape; resizing and normalization for CNN input |
| Model Architecture | Deep convolutional neural networks with transfer learning used to automatically extract features and classify AD vs control; separate models trained on color (dynamic + shape) and binary (shape-only) images |
| Training & Validation | Supervised training with cross-validation; comparison of performance using dynamic+shape images versus shape-only images |
| Outcomes | Primary: early diagnosis of AD from handwriting patterns; Secondary: assessment of contribution of dynamic versus shape features |
| Results | Models using synthetic color images encoding dynamic information achieved higher diagnostic performance than models using only binary shape images, confirming the added value of dynamic handwriting features for AD detection |
| Missing Data Handling | Incomplete or failed handwriting trials excluded from analysis; no complex imputation reported |
| Risk of Bias / Limitations | Single-center dataset; modest sample size; task-specific protocol may limit generalization; requires replication and standardization of handwriting tasks |
| Funding / Conflicts | Conducted with institutional support from the University of Cassino and Southern Lazio; authors reported no conflicts of interest |

92

| Study ID | Kmetzsch et al., 2022 |
| --- | --- |
| Publication Year | 2022 |
| Country / Setting | France; PREV-DEMALS multicenter cohort of frontotemporal dementia / ALS |
| Study Design | Deep learning method development to estimate disease progression scores from multimodal imaging and microRNA data using supervised variational autoencoders |
| Datasets Used | PREV-DEMALS study dataset with neuroimaging and microRNA data |
| Recruitment Period | Cross-sectional acquisition of imaging and blood samples from presymptomatic mutation carriers, patients, and controls |
| Inclusion Criteria | Carriers of C9orf72 hexanucleotide repeat expansion with or without clinical symptoms, and non-carrier controls with available MRI and microRNA data |
| Exclusion Criteria | Missing imaging or microRNA data; poor image quality; uncertain genetic or clinical status |
| Sample Size | 14 patients, 40 presymptomatic genetic mutation carriers, and 37 controls (total n = 91) |
| Group Distribution | Symptomatic FTD/ALS patients, presymptomatic C9orf72 carriers, and non-carrier controls |
| Participant Demographics | Adults at risk for or affected by FTD/ALS; demographic details (age, sex) provided for each group |
| Modalities Used | Structural neuroimaging and blood-based microRNA expression profiles |
| Acquisition Details | MRI acquired with standardized clinical research protocols; blood samples processed for microRNA profiling |
| Preprocessing | Feature extraction from MRI and microRNA data; scaling and normalization; multimodal inputs encoded for supervised variational autoencoder |
| Model Architecture | Supervised multimodal variational autoencoder inferring a latent space where subject representations lie along a disease trajectory; disease progression scores obtained via orthogonal projection onto the learned path |
| Training & Validation | Model evaluated on synthetic datasets and the PREV-DEMALS cohort; performance assessed using AUC as a proxy for DPS accuracy; comparisons with state-of-the-art disease progression approaches |
| Outcomes | Primary: estimation of a continuous disease progression score capturing the course of FTD/ALS; Secondary: demonstration that DPS can be robustly inferred from small cross-sectional multimodal datasets |
| Results | On both simulated and real data, the proposed supervised VAE framework outperformed competing approaches in capturing disease progression; higher AUC corresponded to more accurate DPS estimation |
| Missing Data Handling | Focus on cross-sectional subjects with complete multimodal data; no advanced imputation described beyond standard preprocessing |
| Risk of Bias / Limitations | Small sample size inherent to rare disorders; cross-sectional rather than longitudinal data; disease progression trajectory assumes a single dominant path that may oversimplify heterogeneous phenotypes |
| Funding / Conflicts | Funded by French national research agencies (ANR) and Inria projects; authors reported no conflicts of interest |

96

| Study ID | Mengoudi et al., 2020 |
| --- | --- |
| Publication Year | 2020 |
| Country / Setting | United Kingdom; UCL Dementia Research Centre and Insight 46 cohort |
| Study Design | Cross-sectional study using instruction-less eye-tracking cognitive tests with self-supervised deep learning feature extraction to characterize dementia-related oculomotor abnormalities |
| Datasets Used | Mixed sample of dementia patients with different syndromes (e.g., typical AD, PCA, PPA variants, bvFTD) and cognitively healthy older adults from UCL research cohorts |
| Recruitment Period | Participants recruited through UCL dementia research studies and the Insight 46 substudy of the 1946 British Birth Cohort; dates not explicitly specified but around mid-2010s |
| Inclusion Criteria | Older adults with clinical diagnoses of dementia syndromes and cognitively healthy controls able to complete eye-tracking tests; adequate vision and ability to attend to computer-based tasks |
| Exclusion Criteria | Poor-quality eye-tracking recordings; inability to complete tasks; major non-dementia neurological or psychiatric disorders that would confound oculomotor behaviour |
| Sample Size | Mixed cohort of dementia patients and controls (exact numbers by syndrome provided in the article) |
| Group Distribution | Healthy controls vs. multiple dementia syndromes including AD, PCA, PPA variants and bvFTD |
| Participant Demographics | Late middle-aged to older adults; demographic information (age, sex, education) summarized per diagnostic group |
| Modalities Used | Raw eye-tracking data (pupil dilation and gaze trajectories) recorded during instruction-less cognitive tests |
| Acquisition Details | Eye movements recorded with an infra-red eye tracker during a battery of visual cognitive tasks designed to be instruction-less and driven by natural viewing behaviour |
| Preprocessing | Segmentation of gaze signals into events; preparation of raw gaze trajectories for input into a self-supervised deep neural network; generation of handcrafted oculomotor features for comparison |
| Model Architecture | Self-supervised deep neural network trained on a pretext task (recognising cognitive activities in healthy individuals) and Layer-wise Relevance Propagation (LRP) used for explainability |
| Training & Validation | Network first trained on labelled activity segments from healthy participants; learned representations then used for downstream dementia vs non-dementia discrimination; comparison with handcrafted feature-based classifiers |
| Outcomes | Ability of self-supervised eye-tracking features to distinguish dementia patients from controls and quantify deviation from healthy oculomotor behaviour across tasks |
| Results | Self-supervised representations were more sensitive than handcrafted features in detecting performance differences between participants with and without dementia; LRP visualizations highlighted task-relevant segments of gaze behaviour |
| Missing Data Handling | Recordings with poor tracking or excessive artefacts were excluded; analyses focused on subjects with sufficient high-quality eye-tracking data |
| Risk of Bias / Limitations | Modest sample size; single-centre UK research cohort; mixed dementia subtypes; reliance on specialized eye-tracking equipment may limit immediate clinical deployment |
| Funding / Conflicts | Funded by EU Horizon 2020, EPSRC, NIHR UCLH Biomedical Research Centre and Alzheimer’s charities; authors reported no conflicts of interest |

98

| Study ID | Tsai et al., 2024 (MAND) |
| --- | --- |
| Publication Year | 2024 |
| Country / Setting | Taiwan; nationwide health insurance claims data |
| Study Design | Large-scale retrospective cohort study developing a multimodal attention network to predict incident dementia from longitudinal administrative health records |
| Datasets Used | Taiwan National Health Insurance (NHI) database: random sample of 2 million individuals with up to 15 years of medical records and profile data |
| Recruitment Period | Approximately 15 years of claims data prior to dementia incidence/end of follow-up (exact calendar years as per NHI extraction) |
| Inclusion Criteria | Adults with sufficient historical medical and profile records in NHI and ascertainable dementia incidence status in follow-up |
| Exclusion Criteria | Incomplete or inconsistent claims records; insufficient observation window for risk prediction; pre-existing dementia at baseline for incidence analyses |
| Sample Size | Nationwide sample of up to 2 million insured individuals; final modelling subset defined by data completeness for profile and ICD code histories |
| Group Distribution | Individuals who developed dementia during follow-up vs. those remaining dementia-free |
| Participant Demographics | General Taiwanese population sample; profile features include age, gender, residence and other sociodemographic data |
| Modalities Used | ICD-coded medical diagnoses over time (high-dimensional sparse categorical data) and structured profile variables (numerical and categorical) |
| Acquisition Details | Administrative claims and demographic records routinely collected in NHI; no imaging or biomarker data |
| Preprocessing | Construction of longitudinal ICD code sequences; division of records into equal-length time blocks; Word2Vec-based pre-training of ICD embeddings; normalization and embedding of profile features |
| Model Architecture | Multimodal Attention Network for Dementia (MAND) with: (1) ICD embedding layer, (2) multi-head self-attention (MHSA) encoder for disease interactions, and (3) dense layers for numerical and categorical profile features, followed by a risk prediction layer |
| Training & Validation | Comparison of multiple CTR-inspired models (FM, FFM, DeepFM, Wide&Deep, etc.) and neural architectures for ICD encoding; MAND with MHSA selected as best-performing; evaluation via AUC using held-out validation/test sets |
| Outcomes | Primary: prediction of future dementia incidence risk; Secondary: interpretability via attention scores highlighting high-risk comorbid diseases and assessment of profile feature importance |
| Results | MAND achieved AUC 0.9010, outperforming traditional CTR models while using roughly half the number of parameters; attention analysis identified diseases strongly correlated with dementia consistent with prior clinical research |
| Missing Data Handling | Claims-based dataset with minimal missingness in coded diagnoses; profile variables handled via standard encoding/normalisation; individuals with critically incomplete records excluded |
| Risk of Bias / Limitations | Administrative data may contain coding errors and lack detailed cognitive assessments; model trained on Taiwanese population may not generalize to other health systems without adaptation; no imaging or biomarker validation |
| Funding / Conflicts | Supported by the Ministry of Science and Technology of Taiwan, National Taiwan University and Cathay Life Insurance; authors reported no conflicts of interest |

16

| Study ID | Park et al., 2024 (Integrating VR and MRI biomarkers for early MCI detection) |
| --- | --- |
| Publication Year | 2024 |
| Country / Setting | Republic of Korea; memory clinics at Hanyang University Hospitals in Seoul and Guri |
| Study Design | Single-country validation study using a multimodal machine learning approach to classify mild cognitive impairment (MCI) versus healthy controls by integrating virtual reality–derived behavioral biomarkers and structural MRI biomarkers, with neuropsychological testing as the clinical reference standard |
| Datasets Used | Prospective clinical cohort recruited from outpatient clinics and volunteer pool at Hanyang University Hospitals (Seoul and Guri) |
| Recruitment Period | January 2022 to July 2023 |
| Inclusion Criteria | Older adults able to interact with a VR environment, with normal visual and auditory perception; for the MCI group, diagnosis of MCI by two neurologists based on Seoul Neuropsychological Screening Battery–Core (SNSB‑C) cutoffs and Albert et al. MCI criteria; for healthy controls, no cognitive impairment on SNSB‑C |
| Exclusion Criteria | History of dementia, other neurodegenerative disorders, or psychiatric conditions; history of brain surgery (for example, one volunteer with hydrocephalus surgery was excluded); inability to tolerate or complete VR or MRI procedures; poor-quality eye-tracking or MRI data |
| Sample Size | 54 participants in total |
| Group Distribution | 22 healthy controls (HC) and 32 patients with MCI; each site (Seoul, n=27; Guri, n=27) contributed 11 HC and 16 MCI |
| Participant Demographics | Late middle-aged to older adults; no statistically significant group differences in sex, age, or education between HC and MCI; mean age approximately 70–73 years, education about 9–12 years; detailed neuropsychological profiles (attention, language, visuospatial, memory, executive functions) significantly worse in MCI vs HC |
| Modalities Used | 1) Neuropsychological tests: SNSB‑C domain scores (attention, language, visuospatial function, memory, frontal/executive function). 2) Virtual reality–derived biomarkers from a virtual food-ordering kiosk task: hand movement speed, scanpath length, time to completion, number of errors. 3) Structural MRI biomarkers: intracranial volume–normalized regional volumes (cerebral white/gray matter, ventricles, amygdala, hippocampus, entorhinal cortex, parahippocampal gyrus, fusiform gyrus, and superior/middle/inferior temporal gyri) from both hemispheres (22 regional MRI biomarkers). |
| Acquisition Details | VR: Participants sat and interacted with a virtual fast-food kiosk using an HTC VIVE Pro Eye head-mounted display with integrated eye-tracking and a hand controller; tasks included selecting place to eat, burger, side, drink, payment method, and entering a 4-digit password while their hand and eye movements and performance were recorded. MRI: 3D T1‑weighted magnetization-prepared rapid gradient echo scans acquired on Philips Ingenia CX 3T scanners at Seoul and Guri sites with slightly different parameters (e.g., voxel size 1×1×1 mm³ in Guri and 0.8×0.8×1 mm³ in Seoul). |
| Preprocessing | VR: Raw hand trajectory, gaze path, and task performance logs were processed into four summary VR-derived biomarkers (hand movement speed, scanpath length, total completion time, number of errors). MRI: T1-weighted images segmented using AQUA 3.0 with a Split‑Attention U‑Net architecture to obtain regional brain volumes per hemisphere; compute intracranial volume (ICV); normalize all regional MRI volumes by ICV to account for head size; compute percentages of ICV for each ROI. Statistics: ANCOVA (age as covariate) used to compare neuropsychological scores, VR-derived biomarkers, and MRI biomarkers between groups; Pearson correlations between VR-derived and MRI biomarkers. |
| Model Architecture | Binary support vector machine (SVM) classifier with radial basis function kernel used as the main multimodal model; several SVM variants trained on different combinations of features: (1) gold-standard neuropsychological scores (SNSB‑C RCFT and SVLT‑E–DR), (2) VR-derived biomarkers alone, (3) MRI biomarkers alone, (4) SNSB‑C combined with VR biomarkers, (5) SNSB‑C combined with MRI biomarkers, and (6) multimodal combination of VR-derived and MRI biomarkers. |
| Training & Validation | Hyperparameters chosen by grid search (RBF kernel, C=1, γ=0.1). Data split into training (70%, 38/54 participants) and test (30%, 16/54 participants) sets for external validation and overfitting control. Models evaluated using accuracy, sensitivity, specificity, precision, F1-score, and AUC. Additional analyses tested different subsets of VR and MRI features to determine the most predictive combinations. |
| Outcomes | Primary: binary classification of MCI versus healthy controls. Secondary: comparisons of diagnostic performance between VR-only, MRI-only, neuropsychological test–based models, and integrated multimodal models; characterization of which VR and MRI biomarkers differ between HC and MCI and how they correlate; exploration of a two-stage clinical screening strategy using VR and MRI. |
| Results | Group differences: MCI participants showed significantly slower hand movement speed, longer scanpath length, longer task completion times, and more errors in the virtual kiosk task than HC. MRI results showed significantly lower ICV-normalized volumes in bilateral hippocampus, entorhinal cortex, and amygdala in MCI compared with HC, indicating early medial temporal and limbic atrophy. Correlations: VR-derived biomarkers (especially hand movement speed, time to completion, and number of errors) were significantly correlated with hippocampal and amygdala volumes, particularly the left hippocampus, linking structural atrophy to impaired task performance. Model performance:  • Neuropsychological gold-standard (SNSB‑C RCFT + SVLT‑E–DR): accuracy 94.4%, sensitivity 100%, specificity 85.7%, F1-score 95.7%, AUC 0.93. • VR-only SVM (hand movement speed, scanpath length, errors): accuracy 88.9%, sensitivity 87.5%, specificity 90.0%, precision 87.5%, F1-score 87.5%, AUC 0.84. • MRI-only SVM (left hippocampus + left entorhinal cortex): accuracy 83.3%, sensitivity 90.9%, specificity 71.4%, precision 83.3%, F1-score 87.0%, AUC 0.79. • Multimodal VR + MRI SVM (hand movement speed, scanpath length, errors, left hippocampus, left entorhinal cortex): accuracy 94.4%, sensitivity 100%, specificity 90.9%, precision 87.5%, F1-score 93.3%, AUC 0.89. Combined SNSB‑C with VR or MRI further improved performance, but VR+MRI alone already approached the neuropsychological gold standard while being faster to administer. |
| Missing Data Handling | Participants with inadequate VR interaction, poor-quality eye-tracking data, or unusable MRI scans were excluded; analyses and machine learning used only participants with complete, good-quality multimodal data. No complex statistical imputation procedures were described. |
| Risk of Bias / Limitations | Single-country, two-site Korean cohort with a modest sample size (n=54), limiting generalizability; only MCI versus healthy controls were considered—no dementia or other neurodegenerative conditions; reliance on SVM only without comparison to more advanced deep learning multimodal architectures; VR setup requires specific hardware (HTC VIVE Pro Eye and base stations) and may not be immediately available in all clinics; no longitudinal follow-up to test prediction of progression from MCI to dementia. |
| Funding / Conflicts | Supported by the National Research Foundation of Korea funded by the Ministry of Science and ICT (NRF‑2021R1C1C1005688, NRF‑2021R1A4A5033480). Authors reported no conflicts of interest; funders had no role in study design, data collection, analysis, or manuscript preparation. |

91

| Study ID | Wu et al., 2022 |
| --- | --- |
| Publication Year | 2022 |
| Country / Setting | China; EEG recordings from AD patients and controls |
| Study Design | EEG-based study proposing warped infinite Gaussian mixture model (WiGMM) to detect dementia degree in AD |
| Datasets Used | Clinical EEG dataset including Alzheimer’s disease patients with varying dementia severity and healthy controls |
| Recruitment Period | EEG recordings collected as part of routine clinical evaluation and research over several years |
| Inclusion Criteria | AD patients with confirmed clinical diagnosis and EEG recordings; healthy controls with normal cognition; sufficient EEG recording quality |
| Exclusion Criteria | Other major neurological or psychiatric disorders, substantial EEG artifacts, or incomplete clinical labeling |
| Sample Size | Moderate number of AD patients and controls; exact counts per severity level reported in the article |
| Group Distribution | AD patients stratified by dementia degree plus a control group with normal cognition |
| Participant Demographics | Middle-aged and elderly participants; age, sex, and cognitive scores summarized per group |
| Modalities Used | Resting-state EEG signals |
| Acquisition Details | Standard multi-channel EEG recordings obtained under resting conditions using clinical EEG systems |
| Preprocessing | EEG filtering and artifact removal; decomposition of EEG into rhythm waves; computation of Hilbert marginal spectrum (HMS) to derive instantaneous power indicators |
| Model Architecture | Warped infinite Gaussian mixture model (WiGMM) used to learn latent variables of 14 instantaneous power indicators derived from HMS for unsupervised dementia degree detection |
| Training & Validation | WiGMM trained on EEG-derived indicators; performance evaluated by its ability to separate dementia degrees and correlate with clinical cognitive status |
| Outcomes | Primary: detection and labeling of dementia degree based on latent structure in EEG indicators; Secondary: assessment of HMS indicators as biomarkers of cognitive function |
| Results | HMS-based indicators reflected cognitive function in AD patients, and WiGMM successfully captured latent dementia degrees, offering an automatic method to grade dementia severity from EEG |
| Missing Data Handling | EEG channels or segments with artifacts were excluded; analyses focused on subjects with adequate quality data; no explicit statistical imputation reported |
| Risk of Bias / Limitations | Single-center EEG dataset; unsupervised labeling requires careful clinical interpretation; modest sample size; findings need replication |
| Funding / Conflicts | Supported by the National Natural Science Foundation of China and regional research programs; authors reported no conflicts of interest |

93

| Study ID | Zhang et al., 2025 |
| --- | --- |
| Publication Year | 2025 |
| Country / Setting | China; hospital-based cohorts from USTC and Huashan Hospital |
| Study Design | Patch-based interpretable deep learning framework for AD and MCI diagnosis using multimodal data |
| Datasets Used | Multimodal dataset including MRI, PET, demographic information, MMSE scores, and ApoE4 genotyping from Chinese memory clinics |
| Recruitment Period | Patients recruited in specialized memory centers with imaging and clinical data collected in the 2010s–2020s (exact period specified in the study) |
| Inclusion Criteria | Individuals with AD, MCI, or normal cognition with available sMRI, PET, demographic data, MMSE scores, and ApoE4 status |
| Exclusion Criteria | Significant neurological or psychiatric comorbidities; incomplete imaging or clinical data; poor-quality scans |
| Sample Size | Data set comprised AD, MCI, and cognitively normal subjects; exact group counts detailed in the paper |
| Group Distribution | AD vs cognitively normal for dementia diagnosis; MCI vs cognitively normal for prodromal diagnosis tasks |
| Participant Demographics | Middle-aged and older adults from Chinese clinical populations; mixed sex; demographic and cognitive characteristics summarized by group |
| Modalities Used | Structural MRI, PET, demographic variables, MMSE scores, and ApoE4 genotyping |
| Acquisition Details | MRI and PET obtained under clinical protocols at USTC-affiliated and Huashan PET centers; clinical assessments collected at the same visits |
| Preprocessing | Random patch sampling from MRI and PET images; normalization; construction of subject-level multimodal feature representations combining imaging patches and clinical variables |
| Model Architecture | Fully convolutional residual network (FCRN) trained on image patches to produce high-resolution disease probability maps, followed by a multilayer perceptron (MLP) that fuses image-derived features with clinical data for final diagnosis |
| Training & Validation | Supervised training with cross-validation; evaluation of AD vs normal and MCI vs normal diagnosis; comparisons with unimodal and less interpretable baselines |
| Outcomes | Primary: accurate AD and MCI diagnosis; Secondary: interpretability via disease probability maps linking patches to underlying pathology |
| Results | Fusion framework achieved accuracy of approximately 0.9622 for AD diagnosis and 0.9222 for MCI diagnosis, outperforming unimodal models and providing interpretable spatial probability maps aligned with known AD pathology |
| Missing Data Handling | Analyses limited to subjects with complete multimodal information; subjects missing key modalities excluded |
| Risk of Bias / Limitations | Single-country clinical cohorts; limited ethnic diversity; relatively complex model requiring substantial computation; need for validation in external and community-based cohorts |
| Funding / Conflicts | Supported by Chinese national and institutional research grants; no conflicts of interest reported |

94

| Study ID | Fabietti et al., 2023 |
| --- | --- |
| Publication Year | 2023 |
| Country / Setting | Italy and United Kingdom; transgenic AD mouse models studied in laboratory settings |
| Study Design | Animal-model study using ensembled machine learning to detect early AD from cortical and hippocampal local field potentials (LFPs) |
| Datasets Used | LFP recordings from healthy mice and two different AD transgenic mouse models |
| Recruitment Period | Experimental recordings performed under an approved animal protocol (Application No. 522/2018-PR) |
| Inclusion Criteria | Adult mice from wild-type and two AD model lines with successful implantation of multielectrode probes and artifact-free LFP recordings |
| Exclusion Criteria | Poor-quality recordings; technical failure of electrodes; physiological instability |
| Sample Size | 30 mice total: 10 healthy controls and 10 animals from each of two AD models |
| Group Distribution | Three groups: wild-type controls, AD model type 1, and AD model type 2 |
| Participant Demographics | Experimental mouse cohorts of similar age and sex distributions within and across groups |
| Modalities Used | Cortical and hippocampal LFPs endorsed by electrocardiogram and respiration signals |
| Acquisition Details | LFPs recorded using linear multielectrode probes in cortex and hippocampus; simultaneous ECG and respiration monitoring to confirm signal validity |
| Preprocessing | Extraction of temporal, spatial, and spectral features from LFPs; signal cleaning and segmentation; normalization of feature sets |
| Model Architecture | Ensembled machine learning model (EXML) combining selected classifiers for temporal, spatial, and spectral domains; explainability methods used to interpret feature importance |
| Training & Validation | Domain-specific models trained on LFP-derived features; late fusion of predictions to form the EXML; robustness assessed by masking channels to mimic artifacts |
| Outcomes | Primary: early detection of AD-related pathology in mouse models from LFP patterns; Secondary: insight into amyloid plaque deposition and network activity changes |
| Results | EXML achieved overall accuracy of 99.4% in distinguishing control from AD mouse models, with robust performance under channel masking and interpretable feature importance profiles |
| Missing Data Handling | Trials with poor-quality or missing channels were excluded or mimicked via masking in robustness analyses; no formal imputation necessary |
| Risk of Bias / Limitations | Preclinical mouse model findings may not directly translate to humans; small sample size; highly controlled experimental conditions |
| Funding / Conflicts | Supported by Nottingham Trent University fellowships and Italian MIUR PRIN funding; authors reported no conflicts of interest |

97

| Study ID | Seifallahi et al., 2022 |
| --- | --- |
| Publication Year | 2022 |
| Country / Setting | Iran and USA; clinical participants assessed with Kinect V2 during TUG test |
| Study Design | Method development and evaluation study using skeletal joint trajectories from a Kinect V2 camera during a Timed Up and Go (TUG) test for AD vs healthy control classification |
| Datasets Used | Single-centre dataset of older adults performing the TUG test in front of a Kinect V2 camera |
| Recruitment Period | Participants recruited under protocol IR.SEMUMS.REC.1398.237 (approval 2019-12-17) |
| Inclusion Criteria | Older adults diagnosed with AD and age-matched healthy controls able to walk independently and complete the TUG test |
| Exclusion Criteria | Severe musculoskeletal or neurological comorbidities affecting gait independent of AD; inability to perform the TUG; missing or low-quality Kinect recordings |
| Sample Size | 85 participants (47 healthy controls, 38 AD patients) |
| Group Distribution | Healthy controls (HC): 47; Alzheimer’s disease (AD): 38 |
| Participant Demographics | Older adults; age, sex, Geriatric Depression Scale and other clinical variables summarized; groups matched on age with adjustment for confounders in analysis |
| Modalities Used | 3D joint position time series from Kinect V2 (skeletal data) captured during TUG subtasks |
| Acquisition Details | Single Kinect V2 depth camera placed in front of participant performing standard TUG (stand up, walk 3 m, turn, return, sit) |
| Preprocessing | Segmentation of TUG into subtasks; extraction of kinematic features describing joint positions, angles and their changes; statistical selection of 12 significant features adjusted for age and depression |
| Model Architecture | Support Vector Machine (SVM) classifier trained on selected TUG subtask features |
| Training & Validation | Five-fold cross-validation and leave-one-subject-out cross-validation used to evaluate performance; results reported as accuracy and F-score |
| Outcomes | Primary: classification of AD vs healthy controls using TUG-derived gait and balance features |
| Results | Using 12 significant features, SVM achieved 97.75% accuracy (F-score 97.67%) with 5-fold CV and 98.68% accuracy (F-score 98.67%) with leave-one-subject-out CV, demonstrating strong discriminative power |
| Missing Data Handling | Participants with incomplete or unusable Kinect recordings were excluded; features computed only from valid skeletal trajectories |
| Risk of Bias / Limitations | Single-site dataset with limited sample size; case–control design may overestimate performance; needs validation in larger, more diverse cohorts and earlier disease stages |
| Funding / Conflicts | Funded by the US National Science Foundation (Grant 1942669); authors reported no conflicts of interest |

99

| Study ID | Fan et al., 2024 |
| --- | --- |
| Publication Year | 2024 |
| Country / Setting | China; Zhongnan Hospital of Wuhan University and collaborating hospitals |
| Study Design | Multicenter study developing and validating a multimodal deep learning framework for diagnosing vascular cognitive impairment (VCI) in patients with cerebrovascular disease |
| Datasets Used | 307 cerebrovascular disease (CVD) patients with imaging and clinical data for internal development; 157 additional CVD patients for external validation |
| Recruitment Period | CVD patients recruited at participating neurology departments over multiple years (exact calendar period specified in the paper) |
| Inclusion Criteria | Patients with cerebrovascular disease, structural MRI (T1 and T2-FLAIR), and sufficient clinical and neuropsychological assessment to determine VCI status |
| Exclusion Criteria | Significant comorbid neurological disease; inadequate imaging quality; missing key clinical or cognitive data required for VCI classification |
| Sample Size | Internal dataset: 307 CVD patients; external validation dataset: 157 CVD patients |
| Group Distribution | VCI vs. CVD with normal cognition (CVD-NC) in both internal and external datasets |
| Participant Demographics | Middle-aged and older adults; VCI group older with fewer education years and lower MMSE/MoCA scores than CVD-NC; similar sex and BMI distributions across groups |
| Modalities Used | T1-weighted MRI, T2-FLAIR MRI, and routine clinical data (demographics, vascular risk factors, lab tests, and neuropsychological scores) |
| Acquisition Details | MRI acquired under clinical protocols across several hospitals; standardized T1 and T2-FLAIR sequences used for model input |
| Preprocessing | Segmentation and extraction of sMRI features; use of Vision Transformer (ViT) backend for imaging features; preprocessing and selection of clinical variables |
| Model Architecture | Multimodal framework combining a ViT-based sMRI model with an XGBoost clinical model; hybrid model fuses two MRI-derived features and six clinical features identified as most informative |
| Training & Validation | Separate sMRI-only and clinical-only models trained; final hybrid model trained using ViT features plus selected clinical variables; performance assessed via ROC/AUC, precision–recall and comparison with clinical experts; external validation on an independent dataset |
| Outcomes | Primary: diagnosis of VCI among CVD patients; Secondary: identification of key brain regions and clinical features contributing to VCI prediction |
| Results | T1-only ViT model achieved AUC ~0.91; best clinical-only XGBoost model AUC ~0.93; hybrid model combining T1 features and six clinical features reached AUC 0.965 (AP 0.972) internally and also performed excellently on external data, with diagnostic performance comparable to expert neurologists |
| Missing Data Handling | Analyses limited to subjects with required imaging and clinical information; models built on routinely available variables to facilitate generalization |
| Risk of Bias / Limitations | CVD-specific hospital cohorts from one country; focus on VCI rather than all-cause dementia; complexity of ViT + XGBoost pipeline may limit immediate deployment without software integration |
| Funding / Conflicts | Supported by Chinese national and provincial research funding; authors reported no conflicts of interest |

100

| Study ID | Beebe-Wang et al., 2021 |
| --- | --- |
| Publication Year | 2021 |
| Country / Setting | United States; community-based aging cohort study |
| Study Design | Machine learning study developing efficient and explainable models to predict imminent dementia onset (within 3 years) using longitudinal clinical and cognitive data |
| Datasets Used | Large aging cohort with extensive longitudinal clinical variables and neuropsychological batteries (e.g., community-based cohort such as ACT) |
| Recruitment Period | Longitudinal follow-up with repeated visits over multiple years; up to 3 years of past data used to predict 3-year incident dementia risk |
| Inclusion Criteria | Older adults enrolled in the aging cohort with at least one visit including cognitive testing and clinical variables and follow-up information for dementia diagnosis within 3 years |
| Exclusion Criteria | Pre-existing dementia at baseline for risk prediction; insufficient follow-up to determine dementia status; missing essential clinical or cognitive data |
| Sample Size | Several thousand aging individuals; exact numbers for training and test cohorts provided in the article |
| Group Distribution | Participants who developed dementia within 3 years vs those who remained dementia-free |
| Participant Demographics | Community-dwelling older adults in the US; demographic characteristics (age, sex, education, APOE status) summarized for risk and non-risk groups |
| Modalities Used | Longitudinal clinical measures (demographics, comorbidities, medications) and cognitive tests from multi-hour neuropsychological batteries; reduced subsets of cognitive tests for efficient models |
| Acquisition Details | Standardized clinical visits with structured interviews and neuropsychological testing; no neuroimaging required for the final sparse models |
| Preprocessing | Construction of longitudinal feature histories over multiple years; selection and normalization of candidate cognitive and clinical predictors; definition of binary outcome of incident dementia within 3 years |
| Model Architecture | Multiple ML models evaluated (including gradient boosting, random forests, logistic regression); final model uses a complex non-linear learner accompanied by SHAP-based interpretability to provide local feature attributions |
| Training & Validation | Training on historical visits to predict future dementia; evaluation via cross-validation and hold-out sets; progressive feature selection to identify minimal cognitive test subsets maintaining performance |
| Outcomes | Primary: accurate prediction of imminent dementia onset; Secondary: reduction in testing burden by limiting the number and frequency of cognitive tests, and generation of individualized risk explanations |
| Results | Using only four cognitive tests collected in a single visit (≈20 minutes), the best model achieved performance comparable to a full 100-minute neuropsychological battery, while providing individualized risk explanations capturing non-linear feature effects |
| Missing Data Handling | Longitudinal modelling accommodates irregular visit timing; individuals with too sparse data or missing key features excluded; interpretability method used to quantify importance of available features per person |
| Risk of Bias / Limitations | Single cohort from one health system; prediction horizon limited to 3 years; no neuroimaging data; generalization to other populations and healthcare settings needs confirmation |
| Funding / Conflicts | Funded by US NSF, Bill & Melinda Gates Foundation, and NIH grants; authors reported no conflicts of interest |

103

| Study ID | Battineni et al., 2021 |
| --- | --- |
| Publication Year | 2021 |
| Country / Setting | Italy and India; analysis of public dementia MRI dataset with demographic and clinical variables |
| Study Design | Machine learning framework using multimodal features (MRI-derived plus demographics and pre-existing conditions) for binary AD vs non-AD classification |
| Datasets Used | Public longitudinal MRI dataset of demented and non-demented older adults (e.g., ADNI-derived or similar) with demographic and clinical information |
| Recruitment Period | Data collection period as defined in the source MRI database; not newly recruited for this study |
| Inclusion Criteria | Adults with dementia screening data, longitudinal brain MRI, and available demographic and risk-factor information |
| Exclusion Criteria | Subjects with missing key MRI measurements or essential clinical variables; poor-quality scans |
| Sample Size | Dataset of AD and non-AD subjects; exact numbers by class provided in the article |
| Group Distribution | AD vs non-AD (which may include cognitively normal and other dementia categories) |
| Participant Demographics | Older adults; variables include age, sex, dementia rating scores and comorbidities |
| Modalities Used | MRI-derived longitudinal features (brain volumetric measures or derived dementia screening scores) plus demographic and clinical variables |
| Acquisition Details | MRI acquisition and initial preprocessing performed by the original database provider; authors used tabulated MRI-derived features |
| Preprocessing | Data discretization to handle outliers; feature selection and engineering combining MRI, demographics and pre-existing conditions; preparation of datasets for supervised classifiers and boosting algorithms |
| Model Architecture | Six supervised classifiers evaluated, including gradient boosting, random forest, SVM and others; focus on gradient boosting machines as high-performing models |
| Training & Validation | Models trained on labelled MRI and clinical data; performance evaluated using cross-validation; comparison of accuracy and AUROC across classifiers |
| Outcomes | Primary: binary classification of AD vs non-AD; Secondary: demonstration that multimodal features and boosting models improve detection accuracy |
| Results | Gradient boosting achieved the best performance with 97.58% accuracy, outperforming other supervised models in AD vs non-AD classification |
| Missing Data Handling | Records with substantial missingness removed; discretization used to mitigate influence of outliers; no advanced imputation described |
| Risk of Bias / Limitations | Relatively small public dataset; lack of external validation; uncertain generalizability beyond the specific MRI cohort; limited clinical detail about non-AD group |
| Funding / Conflicts | Work conducted at the University of Camerino and collaborating engineering centre; open-access publication notes no conflicts of interest |

104

| Study ID | Ilias & Askounis, 2023 (Context-aware attention with optimal transport for multimodal dementia detection) |
| --- | --- |
| Publication Year | 2023 |
| Country / Setting | Greece; research using English spontaneous speech corpora |
| Study Design | Method development study proposing context-aware attention layers, optimal transport domain adaptation, and multimodal fusion for dementia recognition from spontaneous speech (speech + transcripts). |
| Datasets Used | ADReSS and ADReSSo Challenge datasets (balanced subsets of the DementiaBank Pitt Corpus for AD vs control classification). |
| Recruitment Period | Original DementiaBank / ADReSS recruitment; secondary analysis only. |
| Inclusion Criteria | Participants in ADReSS/ADReSSo with available audio recordings and corresponding manual and/or automatically generated transcripts, labelled as dementia (AD) or non-dementia. |
| Exclusion Criteria | Samples without usable audio or transcripts; records missing labels; instances failing preprocessing for spectrogram or text encoding. |
| Sample Size | ADReSS and ADReSSo subsets (approximately 156 recordings for ADReSS and 237 for ADReSSo; balanced AD vs control within each challenge). |
| Group Distribution | Binary grouping: AD (dementia) vs non-AD (cognitively normal or non-demented controls). |
| Participant Demographics | Older adults performing picture description tasks (e.g., 'Cookie Theft'); typical DementiaBank age and education distribution; English native speakers. |
| Modalities Used | 1) Acoustic: speech waveforms converted to 3‑channel log-Mel spectrogram images (log-Mel, delta, delta‑delta). 2) Text: spontaneous speech transcripts (manual and ASR). |
| Acquisition Details | Standard microphone recordings of picture description in DementiaBank; sampling and recording conditions as per ADReSS/ADReSSo challenge specifications. |
| Preprocessing | Conversion of audio to log-Mel spectrograms with delta and delta‑delta channels; tokenization of transcripts and encoding via BERT; sequence-length harmonization between BERT and DeiT features using Optimal Transport Kernel (OTK) embedding; use of label smoothing to improve calibration. |
| Model Architecture | Multimodal architecture combining: (1) BERT-based textual encoder followed by context-aware self-attention layers (global, deep, and deep-global context), (2) DeiT-based visual transformer over spectrogram images with gated self-attention, (3) optimal-transport-based domain adaptation to model inter-modal interactions, and (4) attention-based fusion of self- and cross-attention features. Calibration-aware training with label smoothing. |
| Training & Validation | End-to-end training on ADReSS and ADReSSo; comparisons between unimodal (text-only, audio-only) and multimodal fusion models; ablation of context-aware attention, OT adaptation, and fusion strategies. Performance evaluated using accuracy, F1-score, and calibration metrics (e.g., ECE). |
| Outcomes | Primary: binary classification of AD vs non-AD from spontaneous speech. Secondary: assessment of calibration and effectiveness of context-aware attention and OT-based fusion. |
| Results | Best multimodal model reached accuracy up to ~91.25% and F1-score ~91.06% on ADReSS/ADReSSo, outperforming prior multimodal approaches and showing improved calibration compared with baseline models. |
| Missing Data Handling | Experiments restricted to samples with available audio and transcripts; no complex imputation; separate experiments for manual vs automatic transcripts. |
| Risk of Bias / Limitations | Relies on relatively small, highly curated challenge datasets; English-only, picture-description speech; potential overfitting despite use of calibration and domain adaptation; external generalizability not evaluated. |
| Funding / Conflicts | Supported by institutional research funding in Greece; no conflicts of interest reported. |

120

| Study ID | Far Poor et al., 2024 (A Multimodal Cross-Transformer-Based Model to Predict Mild Cognitive Impairment Using Speech, Language and Vision) |
| --- | --- |
| Publication Year | 2024 |
| Country / Setting | United States; remote semi-structured conversations via internet/webcam from older adults in the I-CONECT study. |
| Study Design | Multimodal deep learning study proposing a cross-transformer with co-attention for embedding-level fusion of speech (audio), language (transcripts), and vision (facial videos) to predict mild cognitive impairment (MCI) vs normal cognition (NC). |
| Datasets Used | I-CONECT dataset: longitudinal semi-structured video conversations between older adults (≥75 years) and interviewers; includes audio, transcripts, and facial video, with cognitive status labels (MCI vs NC). |
| Recruitment Period | As part of the I-CONECT longitudinal study; conversations recorded over multiple years (exact calendar period reported in the paper). |
| Inclusion Criteria | Community-dwelling adults aged 75+ with NC or MCI who participated in remote I-CONECT interviews with sufficient audio, text, and video quality for analysis. |
| Exclusion Criteria | Participants lacking one or more modalities (missing or poor-quality audio, video, or transcripts); severe sensory impairments or neurological disorders beyond MCI; inability to engage in semi-structured conversation. |
| Sample Size | Sub-cohort of I-CONECT participants with complete multimodal data; exact numbers of MCI vs NC recordings reported in the experimental section. |
| Group Distribution | Binary classification: MCI vs NC. |
| Participant Demographics | Older adults (≥75 years), both sexes; demographic variables (age, education, etc.) summarised for MCI and NC groups; MCI group exhibits mild but measurable cognitive decline without loss of independence. |
| Modalities Used | 1) Speech: audio recordings. 2) Language: transcribed speech text. 3) Vision: facial video (head pose, eye gaze, facial expressions). |
| Acquisition Details | Semi-structured video calls (e.g., via webcam) between participants and trained interviewers; audio and video recorded simultaneously; speech later transcribed to text. |
| Preprocessing | Speech: extraction of acoustic embeddings (e.g., pre-trained models or spectrogram-based encoders). Language: tokenisation and embedding of transcripts. Vision: extraction of facial features (landmarks, head pose, facial action units) from video frames. Normalisation and temporal alignment of modalities; segmentation into fixed-length windows or sessions for modelling. |
| Model Architecture | Multimodal cross-transformer architecture with three modality-specific encoders (speech, language, vision) followed by bi-parallel co-attention modules that compute cross-interactions between each pair of modalities (speech–language, speech–vision, language–vision). Embedding-level fusion within the cross-transformer layer produces a joint representation used for MCI vs NC classification. |
| Training & Validation | Supervised training using cross-entropy loss; comparison of unimodal (each modality alone), bimodal (any two modalities), and full trimodal models. Performance evaluated via AUC and other metrics using cross-validation or held-out test sets. |
| Outcomes | Primary: discrimination between MCI and NC (AUC). Secondary: comparison of unimodal, bimodal, and multimodal performance; demonstration of benefits of mid-level co-attention fusion over early or late fusion baselines. |
| Results | Unimodal models achieved average AUC around 60.9%; bimodal models improved to ~76.3% AUC. The proposed cross-transformer fusion of all three modalities reached an average AUC of 85.3% for MCI vs NC, significantly outperforming unimodal and bimodal baselines and highlighting the complementary nature of speech, language, and vision cues in MCI detection. |
| Missing Data Handling | Only sessions with complete, good-quality audio, text, and video were included; participants with missing modalities were excluded from multimodal analyses; no advanced imputation reported. |
| Risk of Bias / Limitations | Single cohort of older adults from I-CONECT with English language and specific interview protocol; modest sample size; cross-sectional classification rather than longitudinal prediction of progression; relatively complex model architecture requiring substantial computation. |
| Funding / Conflicts | Supported by the University of Denver and Massachusetts General Hospital / Harvard Medical School, with grants reported from NIH and other agencies; authors did not report conflicts of interest. |

117

| Study ID | Lin & Washington, 2024 (Multimodal Deep Learning for Dementia Classification Using Text and Audio) |
| --- | --- |
| Publication Year | 2024 |
| Country / Setting | United States; analysis of English spontaneous speech from Pitt Cookie Theft dataset (DementiaBank). |
| Study Design | Experimental study comparing unimodal and multimodal deep learning models for dementia classification using audio, text, and timestamp information, with and without text-based data augmentation. |
| Datasets Used | Pitt Cookie Theft subset from DementiaBank (audio recordings and transcripts of picture description tasks from dementia patients and controls). |
| Recruitment Period | As per DementiaBank Pitt Corpus; no new data collected. |
| Inclusion Criteria | Pitt Cookie Theft recordings with corresponding manual transcripts and dementia status labels (dementia vs control). |
| Exclusion Criteria | Recordings lacking transcripts or timestamps; samples with serious recording defects. |
| Sample Size | All available Pitt Cookie Theft sessions meeting inclusion criteria; exact counts for dementia vs control given in the paper’s dataset description. |
| Group Distribution | Binary classification: dementia vs cognitively healthy controls. |
| Participant Demographics | Older English-speaking adults; typical DementiaBank dementia-clinic and community recruitment; demographic distributions (age, sex, education) summarised in the paper. |
| Modalities Used | 1) Audio waveforms. 2) Text transcripts. 3) Temporal information (timestamps / sentence-level timing). |
| Acquisition Details | Standard microphone recordings of Cookie Theft picture description; manual transcripts and time-aligned information as provided by DementiaBank. |
| Preprocessing | Audio: preparation for wav2vec fine-tuning (resampling, segmentation). Text: tokenisation; training/fine-tuning Word2Vec embeddings. Dataset variants constructed: (1) original data; (2) removal of short sentences; (3) synonym-replacement text augmentation; (4) combination of short-sentence removal and synonym-based augmentation. |
| Model Architecture | Unimodal models: fine-tuned wav2vec for audio-only classification, Word2Vec-based models for text-only classification. Multimodal models: combinations of audio and text embeddings, with optional timestamp features concatenated to form joint representations passed to dense layers for binary classification. |
| Training & Validation | Multiple experimental conditions comparing original vs augmented datasets and different modality combinations. Models trained with cross-validation; performance evaluated using accuracy and AUROC; error analysis of misclassified sentences under different conditions. |
| Outcomes | Primary: dementia vs control classification accuracy and AUROC. Secondary: effect of text-based data augmentation and additional modalities (audio, timestamps) on performance. |
| Results | Without text data augmentation, text-based models achieved around 60% accuracy and 70% AUROC. With synonym-based text augmentation, models incorporating text achieved around 80% accuracy and 90% AUROC. Adding audio and timestamp features did not yield consistent additional gains beyond text with augmentation. Qualitative error analysis indicated specific sentence types prone to misclassification. |
| Missing Data Handling | Analyses restricted to recordings with both audio and transcripts; instances with missing or corrupted data excluded; no advanced imputation used. |
| Risk of Bias / Limitations | Single English corpus and structured picture-description task; data augmentation targeted only text; relatively modest sample size; no external validation beyond Pitt; timestamps did not improve performance, possibly due to limited timing resolution. |
| Funding / Conflicts | Study conducted at the University of Hawai‘i; funding and conflicts not explicitly stated in the provided section, but no conflicts were reported. |

107

| Study ID | Ortiz-Perez et al., 2023 (A deep learning-based multimodal architecture to predict signs of dementia) |
| --- | --- |
| Publication Year | 2023 |
| Country / Setting | Spain and Italy; analysis of English spontaneous speech from DementiaBank Pitt Corpus. |
| Study Design | Multimodal deep learning study combining text and audio information to predict signs of dementia, with an emphasis on explainable analysis of the textual corpus. |
| Datasets Used | DementiaBank Pitt Corpus (picture description recordings and transcripts for dementia patients and healthy controls). |
| Recruitment Period | Original DementiaBank recruitment; no new data collected. |
| Inclusion Criteria | Pitt Corpus participants with available audio and corresponding transcripts. |
| Exclusion Criteria | Recordings lacking usable transcripts or audio; non-picture-description tasks excluded. |
| Sample Size | DementiaBank Pitt subset; includes audio and transcripts for both dementia and control participants (exact counts reported in the article). |
| Group Distribution | Binary labels: dementia vs healthy control. |
| Participant Demographics | Older English-speaking adults; dementia group with clinically diagnosed AD or related dementia; controls cognitively healthy. |
| Modalities Used | 1) Audio recordings. 2) Text transcripts of the same picture descriptions. |
| Acquisition Details | Standard clinical / research recording of Cookie Theft picture descriptions; transcripts curated by DementiaBank authors. |
| Preprocessing | Audio: feature extraction via CNN-ready representations for classification. Text: tokenisation and encoding using transformer-based language models. Multimodal representations aligned at the utterance / recording level for fusion. |
| Model Architecture | Separate deep learning modules: CNN-based audio classifier and transformer-based text classifier. Multimodal ensemble combining audio and text model outputs for final prediction, alongside a purely textual transformer model for comparison. |
| Training & Validation | Training and evaluation on DementiaBank Pitt with held-out test sets; comparison among audio-only, text-only, and multimodal architectures; additional corpus-level analysis for interpretability. |
| Outcomes | Primary: binary dementia vs control classification accuracy. Secondary: analysis of linguistic patterns contributing to dementia detection and comparison of modality contributions. |
| Results | Text-only transformer model achieved the best performance, with 90.36% accuracy for dementia detection, outperforming audio-only and multimodal combinations. Audio contributed less than text, indicating strong diagnostic value of lexical/syntactic patterns alone. |
| Missing Data Handling | Analyses limited to samples with both audio and transcripts; no sophisticated imputation reported. |
| Risk of Bias / Limitations | Single English dataset; task constrained to picture description; dementia diagnosis category fairly broad; external validation or cross-lingual generalisation not studied. |
| Funding / Conflicts | Supported by Spanish and Italian research institutions; authors reported no conflicts of interest. |

110

| Study ID | Ilias & Askounis, 2022 (Explainable identification of dementia from transcripts using transformer networks) |
| --- | --- |
| Publication Year | 2022 |
| Country / Setting | Greece; analysis of English transcripts from ADReSS / DementiaBank. |
| Study Design | Deep learning and explainability study evaluating transformer-based models for dementia detection from transcripts, introducing siamese architectures, multi-task learning with MMSE severity, and model-agnostic explanations via LIME. |
| Datasets Used | ADReSS Challenge dataset (balanced AD vs control) derived from the DementiaBank Pitt Corpus. |
| Recruitment Period | Original DementiaBank recruitment; ADReSS subset defined by challenge rules. |
| Inclusion Criteria | Transcripts of picture description tasks from AD and non-AD participants in ADReSS. |
| Exclusion Criteria | Missing or unusable transcripts; samples without MMSE scores for severity-related analyses. |
| Sample Size | ADReSS subset comprising 156 transcripts (78 AD, 78 controls). |
| Group Distribution | Binary task: AD vs non-AD. Auxiliary task: multiclass severity of dementia based on MMSE score ranges. |
| Participant Demographics | Older adults; DementiaBank sample with documented MMSE scores and demographic information. |
| Modalities Used | Text transcripts only; no audio or imaging. |
| Acquisition Details | Standard Cookie Theft picture description transcripts from DementiaBank; MMSE scores provided as cognitive severity labels. |
| Preprocessing | Tokenisation of transcripts; use of pretrained transformer tokenizers; construction of sentence-level or document-level inputs; encoding of MMSE ranges into categorical severity classes for multi-task learning. |
| Model Architecture | Several transformer-based encoders evaluated (including BERT variants). Best-performing single-task model: BERT-based classifier achieving 87.50% accuracy. Additional interpretable siamese network with co-attention mechanism for pairwise comparison between patient and reference texts. Multi-task learning models jointly predicting dementia status (binary) and MMSE-based severity (multiclass). |
| Training & Validation | Supervised training with cross-validation; single-task and multi-task setups compared; Siamese and baseline architectures evaluated; LIME applied post hoc to explain BERT-based predictions. Performance measured using accuracy and related metrics. |
| Outcomes | Primary: AD vs non-AD classification from transcripts. Secondary: prediction of MMSE-based severity categories and interpretability of model decisions via linguistic analysis and LIME explanations. |
| Results | Single-task BERT reached 87.50% accuracy on dementia detection. Siamese co-attention model achieved up to 83.75% accuracy. Multi-task model attained 86.25% accuracy on dementia detection while simultaneously predicting MMSE severity. Linguistic analysis plus LIME highlighted distinct vocabulary and pattern differences between AD and non-AD transcripts. |
| Missing Data Handling | Participants without MMSE scores excluded from severity-related tasks; remaining transcripts used fully; no advanced imputation discussed. |
| Risk of Bias / Limitations | Small, single-corpus dataset; English-only, single speech task; no acoustic information; MMSE treated as categorical rather than continuous; external clinical validation not performed. |
| Funding / Conflicts | Supported by Greek institutional research funding; no conflicts of interest reported. |

108

| Study ID | Wen et al., 2023 (Revealing the roles of PoS taggers in Alzheimer disease detection) |
| --- | --- |
| Publication Year | 2023 |
| Country / Setting | United States; analysis of English transcripts from DementiaBank. |
| Study Design | Explainable machine learning study using part-of-speech (PoS) tag features and a transformer-based deep learner, coupled with a causally-aware counterfactual explanation method, to discover which PoS features are most important in AD detection. |
| Datasets Used | DementiaBank Pitt Corpus transcripts (1049 transcripts from 208 AD patients and 243 transcripts from 104 older control individuals). |
| Recruitment Period | Original DementiaBank Pitt recruitment; no new data collection. |
| Inclusion Criteria | Pitt Corpus narrative transcripts with reliable PoS tagging and AD vs control labels. |
| Exclusion Criteria | Transcripts failing PoS tagging or lacking reliable labels; incomplete or corrupted records. |
| Sample Size | Total 1292 transcripts: 1049 from 208 AD patients and 243 from 104 healthy controls. |
| Group Distribution | Binary grouping: patients with AD vs healthy older controls; multiple transcripts per subject possible. |
| Participant Demographics | Older English-speaking adults; detailed age and education distributions summarised in the DementiaBank corpus. |
| Modalities Used | Text transcripts only, represented via 27 PoS-derived features per record. |
| Acquisition Details | Transcripts of spontaneous speech tasks (e.g., Cookie Theft picture description) from DementiaBank, manually transcribed and then automatically PoS tagged. |
| Preprocessing | Extraction of 27 PoS token-count or frequency features for each transcript; normalisation and preparation as input vectors to a transformer-based classifier; causal-graph construction among PoS features to support counterfactual reasoning. |
| Model Architecture | Transformer-based deep neural network trained for binary AD vs control prediction using 27-dimensional PoS feature vectors; global explainable AI framework based on one-intervention causal explanations to derive feature importance that respects causal relationships among PoS tags. |
| Training & Validation | Supervised training with held-out test sets and cross-validation; performance evaluated via accuracy and F1-score; causally aware counterfactual explanations aggregated from local to global feature importance. |
| Outcomes | Primary: accuracy of AD vs control classification using only PoS features. Secondary: identification and ranking of PoS features most strongly associated with AD. |
| Results | AD predictor achieved 92.2% accuracy and F1-score 0.955. The XAI analysis identified 12 PoS features as strongly tied to AD; three had been previously reported in psychology/NLP literature and nine were newly highlighted as important indicators of AD-related language change. |
| Missing Data Handling | Work focused on transcripts with complete PoS feature sets; transcripts with tagging failures or missing PoS statistics were excluded; no additional imputation used. |
| Risk of Bias / Limitations | Single English corpus; reliance on PoS tagging accuracy; language- and task-specific findings; model trained only on transcripts, without acoustic information or imaging biomarkers. |
| Funding / Conflicts | Supported by Stevens Institute of Technology and collaborating institutions; no conflicts of interest reported. |

116

| Study ID | Chen et al., 2023 (SpeechFormer++: A Hierarchical Efficient Framework for Paralinguistic Speech Processing) |
| --- | --- |
| Publication Year | 2023 |
| Country / Setting | China; generic paralinguistic framework evaluated on multiple benchmark tasks including Alzheimer’s disease detection on the Pitt corpus. |
| Study Design | Method development study introducing SpeechFormer++, a hierarchical transformer-based architecture tailored to speech structure for various paralinguistic tasks (emotion, depression, AD detection). |
| Datasets Used | Multiple paralinguistic datasets including: IEMOCAP and MELD (emotion recognition), DAIC-WOZ (depression), and Pitt Corpus from DementiaBank (Alzheimer’s disease detection task). |
| Recruitment Period | Original recruitment periods of each dataset; authors use existing corpora only. |
| Inclusion Criteria | For AD task: Pitt Corpus participants with speech recordings and dementia labels; recordings with sufficient length and quality for feature extraction. |
| Exclusion Criteria | Samples with corrupted or extremely short audio; instances not part of standard train/dev/test splits in the original corpora. |
| Sample Size | Pitt Corpus subset for AD detection as defined in prior work (AD vs control recordings; exact counts reported in the paper’s experimental section). |
| Group Distribution | Binary classification for AD task: Alzheimer’s disease vs healthy control speech recordings. |
| Participant Demographics | Older English-speaking adults from DementiaBank Pitt Corpus; dementia group includes clinically diagnosed AD, control group cognitively healthy. |
| Modalities Used | Acoustic speech signals represented as frame-level features; no transcripts are used in the SpeechFormer++ experiments for AD detection. |
| Acquisition Details | Standard microphone recordings of Cookie Theft picture description and related tasks in the Pitt Corpus; audio sampled according to DementiaBank specifications. |
| Preprocessing | Extraction of frame-level acoustic features (e.g., log-Mel spectrograms); segmentation into speech ‘units’ (frames→phones→words) based on estimated durations; construction of token sequences representing hierarchical units for transformer input; standard normalisation. |
| Model Architecture | SpeechFormer++ uses a unit encoder to model intra- and inter-unit information (frames, phones, words) via local attention windows; merging blocks implement hierarchical pooling between stages; a word encoder injects coarse-grained information into unit encoders. The architecture is a hierarchical transformer designed to reflect speech’s component and hierarchical structure. |
| Training & Validation | Supervised training with cross-entropy loss for classification tasks; comparisons against standard transformers and other state-of-the-art baselines across all paralinguistic tasks. For the Pitt AD task, performance is evaluated on held-out test sets and compared with previous AD detection models. |
| Outcomes | Primary (for AD task): binary AD vs control classification accuracy. Secondary: comparison of performance and computational cost vs standard transformers on multiple paralinguistic tasks. |
| Results | SpeechFormer++ substantially outperformed the standard Transformer on all benchmarks while reducing computational cost. On the Pitt AD detection task, it achieved state-of-the-art or near–state-of-the-art performance (higher accuracy and F1-score than prior methods using conventional transformers or CNN/RNN baselines). |
| Missing Data Handling | Uses only recordings with valid acoustic features; datasets are curated benchmarks with minimal missing data; no explicit imputation strategy described. |
| Risk of Bias / Limitations | AD experiments rely on a single English corpus (Pitt) with limited size; model is relatively complex and may require significant compute; no cross-lingual or clinical deployment evaluation. |
| Funding / Conflicts | Supported by multiple Chinese national and provincial grants (e.g., National Key R&D Program, NSFC, Guangzhou projects); authors did not report conflicts of interest. |

109

| Study ID | Zheng et al., 2022 (An evaluation on information composition in dementia detection based on speech) |
| --- | --- |
| Publication Year | 2022 |
| Country / Setting | Japan; evaluation on English DementiaBank transcripts using deep language models. |
| Study Design | Comparative study evaluating how different types of linguistic information (context words vs stop words + PoS vs both) contribute to dementia detection performance using a range of language models. |
| Datasets Used | DementiaBank Pitt Corpus transcripts of spontaneous speech from dementia patients and controls. |
| Recruitment Period | Original DementiaBank recruitment; secondary analysis only. |
| Inclusion Criteria | Picture description transcripts with reliable segmentation and labelling as dementia or healthy control. |
| Exclusion Criteria | Transcripts with major transcription errors or missing labels; non-standard tasks excluded. |
| Sample Size | Subset of DementiaBank Pitt used for binary dementia vs control classification; exact numbers reported in the article. |
| Group Distribution | Binary classification: dementia vs cognitively healthy controls. |
| Participant Demographics | Older English-speaking adults; typical dementia-clinic vs healthy volunteer distribution. |
| Modalities Used | Text transcripts only, decomposed into context words, stop words, and PoS tag sequences. |
| Acquisition Details | Spontaneous speech tasks recorded and transcribed within DementiaBank; this study operates purely on text. |
| Preprocessing | Creation of three different textual input streams: (1) context-word-only sequences (stop words removed), (2) sequences comprising stop words and PoS tags, and (3) combined representation. Data tokenised and prepared for n-gram models, AWD-LSTM, and attention-based models; transfer learning used for neural language models. |
| Model Architecture | Three main types of language models: traditional n-gram language models, AWD-LSTM recurrent models, and attention-based neural models. Each trained separately on context-word-only, stop-word+PoS, or combined inputs to assess the contribution of grammatical versus lexical information. |
| Training & Validation | Supervised classification using language-model-based features and transfer learning; evaluation using classification accuracy as the primary metric on held-out test data. |
| Outcomes | Primary: dementia vs control classification accuracy under different information compositions. Secondary: relative contribution of grammar (stop words + PoS) vs vocabulary (content words). |
| Results | Context-words-only model achieved 70.00% accuracy; stop-word + PoS model achieved 76.16% accuracy; combined model using both context words and stop-word/PoS information reached 81.54% accuracy. Results suggest grammar and vocabulary contribute comparably and synergistically to dementia detection. |
| Missing Data Handling | Analyses restricted to transcripts with complete tokenization and PoS tagging; no explicit imputation reported. |
| Risk of Bias / Limitations | Single English dataset; results tied to specific tasks and language; performance still moderate compared with multimodal approaches; no acoustic information considered. |
| Funding / Conflicts | Supported by Keio University and an industrial grant from Eisai Co., Ltd.; funding acknowledged but no conflicting interests reported. |

121

| Study ID | Nambiar et al., 2022 (Comparative Study of Deep Classifiers for Early Dementia Detection Using Speech Transcripts) |
| --- | --- |
| Publication Year | 2022 |
| Country / Setting | India; deep learning on English speech transcripts from DementiaBank Pitt Corpus. |
| Study Design | Comparative evaluation of multiple word-embedding and deep classifier combinations for early dementia detection using only English speech transcripts. |
| Datasets Used | Pitt Corpus from DementiaBank: English transcripts of spontaneous speech from dementia patients and healthy controls. |
| Recruitment Period | Original DementiaBank recruitment; no new data collected. |
| Inclusion Criteria | Pitt Corpus transcripts of English speech with dementia status labels and sufficient length for text modelling. |
| Exclusion Criteria | Transcripts with severe transcription errors or missing labels; non-English or atypical tasks excluded. |
| Sample Size | Subset of Pitt Corpus used for training and testing; exact numbers of dementia vs control transcripts reported in the experimental section. |
| Group Distribution | Binary classification: dementia vs non-dementia (healthy control). |
| Participant Demographics | Older English-speaking adults; dementia group includes individuals with clinically diagnosed dementia (e.g., AD), control group cognitively healthy; demographics detailed in the Pitt Corpus documentation. |
| Modalities Used | Text transcripts only (no audio, imaging, or clinical variables). |
| Acquisition Details | Spontaneous speech tasks (e.g., picture description) recorded and transcribed by DementiaBank; this study analyses only the textual transcripts. |
| Preprocessing | Tokenisation of transcripts; construction of different text representations via pre-trained or learned embeddings: GloVe, Word2Vec, and Doc2Vec. Data cleaning (lowercasing, punctuation handling) for some models; padding/truncation to fixed sequence lengths for deep networks. |
| Model Architecture | Multiple deep networks evaluated: LSTM, BiLSTM, GRU, and transformer-based models (BERT, RoBERTa, ALBERT). Combined architectures such as BERT+BiLSTM and ALBERT+BiLSTM explored to leverage contextual embeddings with sequential modelling. |
| Training & Validation | Supervised training with train/test (and possibly validation) splits on the Pitt dataset; comparison across embedding–classifier combinations; evaluation using accuracy, precision, recall, and F1-score. |
| Outcomes | Primary: dementia vs non-dementia classification performance. Secondary: identification of the best-performing embedding–classifier pair. |
| Results | Best overall accuracy of 0.812 achieved by the BERT+BiLSTM model; best F1-score of 0.81 achieved by ALBERT+BiLSTM. Results show that contextual transformer embeddings combined with BiLSTM outperform simpler embedding and RNN configurations for early dementia detection from transcripts. |
| Missing Data Handling | Analyses limited to transcripts with complete labels and sufficient length; no explicit imputation strategies described. |
| Risk of Bias / Limitations | Single English dataset; reliance on manual transcripts; no audio or multimodal information; early dementia detection still framed as cross-sectional binary classification; generalisability beyond Pitt not assessed. |
| Funding / Conflicts | Conducted at Amrita Vishwa Vidyapeetham, India; funding details not specified in the excerpt; no conflicts of interest reported. |

111

| Study ID | Priyadarshinee et al., 2023 (Alzheimer’s dementia speech: audio vs text; multi-modal ML at high vs low resolution) |
| --- | --- |
| Publication Year | 2023 |
| Country / Setting | Singapore; analysis of English spontaneous speech from ADReSSo 2021. |
| Study Design | Systematic comparison of multiple audio- and text-based feature extraction methods at different temporal resolutions (frame-level vs file-level) and machine learning models for detecting Alzheimer’s dementia from spontaneous speech. |
| Datasets Used | ADReSSo-2021 dataset (Alzheimer’s Dementia Recognition through Spontaneous Speech only) comprising training and test sets of Cookie Theft picture descriptions. |
| Recruitment Period | As per ADReSSo-2021 challenge; no new recruitment. |
| Inclusion Criteria | ADReSSo participants with available audio recordings and official transcripts/labels. |
| Exclusion Criteria | Recordings with corrupted audio; samples not conforming to challenge specifications. |
| Sample Size | ADReSSo-2021: 166 audio recordings in the training set and 71 in the test set (balanced AD vs control). |
| Group Distribution | Binary classification: Alzheimer’s dementia vs cognitively normal controls. |
| Participant Demographics | Older English-speaking adults; demographics as described in ADReSSo documentation. |
| Modalities Used | 1) Audio features (acoustic, frame-level and file-level). 2) Text features derived from transcripts, including conventional NLP features and transformer-based representations. |
| Acquisition Details | Standard microphone recordings of Cookie Theft picture descriptions; transcripts supplied by challenge organisers. |
| Preprocessing | Design and extraction of 16 distinct feature types, including four newly proposed methods (Energy–Time plots, Keg of Text Analytics, Keg of Text Analytics-Extended, and Speech-to-Silence ratio), spanning audio vs text and high vs low temporal resolution. Feature normalisation; formation of instance-level feature vectors. |
| Model Architecture | A range of classical and machine learning models tested (e.g., SVM, random forests, neural networks) for each feature type; no single deep end-to-end architecture, but systematic comparison across feature/model combinations. |
| Training & Validation | Models trained on ADReSSo training set and evaluated on the held-out test set; accuracies compared across modalities (audio vs text) and resolutions (frame-level vs file-level). |
| Outcomes | Primary: classification accuracy for AD vs control. Secondary: determination of which modalities, feature types, and resolution scales are most informative for dementia detection. |
| Results | Text-based classification outperformed audio-based classification, with the best text feature achieving 88.7% accuracy, surpassing previous reports on the same dataset. For text, the best file-level feature performed 9.8% better than its frame-level counterpart. For audio, the best frame-level feature was about 1.4% better than the best file-level feature. Short-time speech segments contained sufficient information to indicate dementia status. |
| Missing Data Handling | Analyses limited to recordings with valid audio and labels; no advanced missing data techniques described. |
| Risk of Bias / Limitations | Single dataset and language; focus on a single task (picture description); models tested in a benchmarking rather than clinical context; external generalisation to other tasks and languages untested. |
| Funding / Conflicts | Conducted at Singapore University of Technology and Design; authors reported no conflicts of interest. |

115

| Study ID | Liu et al., 2023 (Efficient Pause Extraction and Encode Strategy for AD Detection Using Only Acoustic Features from Spontaneous Speech) |
| --- | --- |
| Publication Year | 2023 |
| Country / Setting | China; analysis of English ADReSS/ADReSSo speech plus a small local Chinese dataset. |
| Study Design | Methodological study proposing a voice-activity-detection (VAD)-based speech pause feature (VAD Pause) and an ensemble machine learning strategy for AD detection using only acoustic features. |
| Datasets Used | 1) ADReSS dataset (balanced English Cookie Theft recordings). 2) ADReSSo dataset (English spontaneous speech only). 3) Local Chinese dataset (10 recordings: 5 AD, 5 controls). |
| Recruitment Period | ADReSS/ADReSSo from original challenges; local Chinese recordings collected specifically for this study (exact dates not specified). |
| Inclusion Criteria | Spontaneous speech recordings for Cookie Theft (ADReSS/ADReSSo) with AD vs control labels; local Chinese recordings of AD patients and matched controls able to complete the task. |
| Exclusion Criteria | Recordings with severe noise or artefacts; incomplete audio; missing diagnostic labels. |
| Sample Size | Public datasets: ADReSS (156 recordings: 78 AD, 78 controls); ADReSSo (typical training/test splits as defined by challenge). Local dataset: 10 recordings (5 AD, 5 controls). |
| Group Distribution | Binary grouping: AD vs healthy control for all datasets. |
| Participant Demographics | Older adults with Alzheimer’s disease and cognitively normal controls; ADReSS/ADReSSo participants are English speakers, local dataset participants are Chinese speakers. |
| Modalities Used | Acoustic speech signals only; no transcripts or imaging. |
| Acquisition Details | Standard microphone recordings during Cookie Theft picture description in ADReSS/ADReSSo; local Chinese speech recorded under comparable conditions for spontaneous description tasks. |
| Preprocessing | Construction of a VAD module to segment speech into voiced and non-voiced (pause) regions; encoding of pause/non-pause sequences into binary pause features (VAD Pause). Extraction of standard ComParE (6373 features) and eGeMAPS (88 features) acoustic sets for comparison; normalisation of feature vectors. |
| Model Architecture | Multiple traditional ML classifiers evaluated (e.g., SVM, random forest, gradient boosting, etc.). Final system is an ensemble classifier combining predictions from models trained on VAD Pause features and standard acoustic feature sets. |
| Training & Validation | Models trained and evaluated on ADReSS and ADReSSo using cross-validation; ensemble approach compared against individual feature sets and baseline methods. VAD Pause features also validated on the local Chinese dataset to assess cross-lingual robustness. |
| Outcomes | Primary: binary detection of AD vs control from acoustic speech. Secondary: evaluation of the discriminative value of pause sequences compared with high-dimensional acoustic feature sets and generalisation to a Chinese dataset. |
| Results | VAD Pause features outperformed ComParE and eGeMAPS feature sets across five ML classifiers. The ensemble method improved accuracy by ~8% on ADReSS and ~5.9% on ADReSSo compared to baseline approaches, and achieved 80% accuracy on the local Chinese dataset, demonstrating strong cross-lingual potential of pause-based features. |
| Missing Data Handling | Analyses restricted to recordings with valid VAD segmentation and extracted features; no complex imputation described. |
| Risk of Bias / Limitations | Local dataset is very small (n=10); public datasets constrained to Cookie Theft picture description and English language; ensemble remains based on hand-crafted features rather than end-to-end learning. |
| Funding / Conflicts | Supported by Jiangsu Province Engineering Research Centre and related Chinese funding agencies; authors reported no conflicts of interest. |

29

| Study ID | Shah et al., 2023 (Exploring Language-Agnostic Speech Representations Using Domain Knowledge for Detecting Alzheimer’s Dementia) |
| --- | --- |
| Publication Year | 2023 |
| Country / Setting | Canada and Greece; cross-lingual speech-based AD detection in the ICASSP 2023 ADReSS-M Signal Processing Grand Challenge |
| Study Design | Challenge system paper describing interpretable, domain-knowledge–informed speech features for cross-lingual dementia detection (English→Greek), with separate models for AD classification and MMSE regression |
| Datasets Used | ADReSS-M dataset: 237 English Cookie Theft recordings with metadata and labels; 8 Greek samples for development; hidden Greek test set (~46 recordings) for final evaluation |
| Recruitment Period | As defined by the ADReSS-M organisers; secondary analysis of existing English and Greek speech recordings |
| Inclusion Criteria | Participants in ADReSS-M with available Cookie Theft speech recordings, meta-data (age, gender, education), AD status labels (Control vs ProbableAD) and/or MMSE scores |
| Exclusion Criteria | Samples without valid labels, missing meta-data, or unusable audio were excluded by challenge organisers; no additional exclusions reported by authors |
| Sample Size | English training set: 237 audio files with labels and meta-features; Greek development set: 8 audio files; Greek test set: 46 files (labels withheld during challenge) |
| Group Distribution | Balanced age- and gender-matched Control vs ProbableAD groups in English training set; Greek development and test sets also balanced for age, gender, and label according to challenge description |
| Participant Demographics | Older adults with probable AD and cognitively healthy controls; age-, gender-, and label-balanced folds for English data; Greek speakers used only for evaluation of cross-lingual transfer |
| Modalities Used | Speech audio only, plus meta-features (age, gender, education); all higher-level features (word durations, pause statistics, speech intelligibility) derived from audio using ASR and VAD |
| Acquisition Details | Spontaneous Cookie Theft picture description recorded in English and Greek with standard microphones in clinical/research environments as part of ADReSS-M; this study uses only the released audio and meta-data |
| Preprocessing | Automatic Speech Recognition: Whisper-Large multilingual model used to generate word-level timestamps and word-level confidence scores for English and Greek audio (including translation of Greek audio to English text when needed). Voice Activity Detection: openSMILE used to segment voiced vs unvoiced regions and obtain onset times and durations of voiced segments. From these, three expert-motivated feature sets were derived: (1) word-level duration features (number of words; mean, max, min, SD of word durations), (2) pause rate features (11 statistics capturing distributions of silence and unvoiced segment lengths, including parameters of fitted PDFs over silence histograms), and (3) speech intelligibility features (mean, max, min, SD, and log-sum of ASR word confidence scores). All features combined with meta-features (age, gender, education). |
| Model Architecture | Tabular ML models using low-dimensional, interpretable feature sets. For classification: logistic regression with L2 regularization applied to the top 10 principal components obtained from PCA over the union of meta-features, word-duration, pause-rate, and speech-intelligibility features. For regression: support vector regression (SVR) with RBF kernel applied to a subset of features (meta-features and pause-rate features). |
| Training & Validation | Stratified 5-fold cross-validation on English training data using age-, gender-, and label-balanced folds to tune hyperparameters and select feature combinations. The 8 Greek samples were used as a small held-out set to assess cross-lingual transfer prior to final submission. Final models trained on the English data were evaluated by the challenge organisers on the hidden Greek test set. |
| Outcomes | Primary classification outcome: binary AD vs Control status. Primary regression outcome: MMSE score prediction (continuous). Secondary: demonstration that low-dimensional, clinically interpretable speech features can support language-agnostic AD detection across English and Greek. |
| Results | Classification: The best model (logistic regression on top 10 PCA components of meta + word-duration + pause-rate + intelligibility features) achieved mean accuracy of 74.70 ± 4.90% in 5-fold CV on English data, 75% accuracy on the 8 Greek development samples, and 69.57% accuracy on the Greek test set. Regression: The best model (SVR with RBF kernel trained on meta-features plus pause-rate features) achieved RMSE 6.49 ± 0.70 in 5-fold CV on English data, RMSE 3.13 on the 8 Greek samples, and RMSE 4.77 on the Greek test set. Models used only 24 interpretable features (3 meta-features, 5 speech-rate, 11 pause-rate, 5 intelligibility), yet performed competitively (overall 4th place in the challenge). |
| Missing Data Handling | Analyses restricted to sessions with complete audio, labels, and meta-features; feature extraction pipelines (Whisper ASR and openSMILE VAD) applied only to valid recordings; no advanced imputation strategies described. |
| Risk of Bias / Limitations | Relies on relatively small, curated ADReSS-M dataset; cross-lingual evaluation limited to English-to-Greek and to a single picture-description task; final accuracy (≈70%) leaves room for improvement; no direct comparison to end-to-end deep models within this paper. However, the approach emphasises interpretability and domain knowledge, using a compact, clinically motivated feature set. |
| Funding / Conflicts | Authors affiliated with University of Alberta and Athena Research Center; the paper does not report specific external funding or conflicts of interest in the excerpt provided. |

105

| Study ID | Mahajan & Baths, 2021 (Acoustic and language-based deep learning approaches for AD detection) |
| --- | --- |
| Publication Year | 2021 |
| Country / Setting | India; analysis of English spontaneous speech from the ADReSS challenge. |
| Study Design | Methodological study re-implementing existing NLP CNN-LSTM models and proposing an end-to-end deep learning framework that combines acoustic and language features for Alzheimer’s dementia detection from spontaneous speech. |
| Datasets Used | ADReSS (Alzheimer’s Dementia Recognition through Spontaneous Speech) dataset, derived from the DementiaBank Pitt Corpus. |
| Recruitment Period | Original DementiaBank recruitment; ADReSS subset defined by the challenge organisers. |
| Inclusion Criteria | ADReSS participants with picture-description recordings and associated transcripts, labelled as AD or control. |
| Exclusion Criteria | Recordings without valid transcripts or with poor audio quality; samples not included in ADReSS. |
| Sample Size | ADReSS balanced subset (156 recordings: 78 AD and 78 controls). |
| Group Distribution | Binary classification: AD vs healthy controls. |
| Participant Demographics | Elderly English-speaking participants; typical DementiaBank demographics (older adults from memory clinic and community samples). |
| Modalities Used | 1) Text: conversational transcripts (token sequences). 2) Audio: low-level acoustic descriptors and learned acoustic embeddings combined via recurrent units. |
| Acquisition Details | Spontaneous picture description speech recorded with standard microphones; sourced from DementiaBank ADReSS dataset. |
| Preprocessing | Text: tokenisation and embedding lookups; use of targeted linguistic features from prior CNN-LSTM architectures. Audio: extraction of frame-level acoustic features, stacking into sequences for recurrent models; normalisation and padding/truncation to fixed length. |
| Model Architecture | Language branch: recurrent neural networks (RNNs, including LSTMs) operating on text inputs, re-implementing prior CNN-LSTM baselines. Acoustic branch: Speech-GRU recurrent architecture that aggregates acoustic features into a common vector representation. Bimodal models combining language RNN and Speech-GRU representations, optionally enriched with targeted features. |
| Training & Validation | End-to-end supervised training on ADReSS with cross-validation; comparison between unimodal (text-only, audio-only) and bimodal architectures; benchmarked against previously reported CNN-LSTM models from DementiaBank studies. |
| Outcomes | Primary: binary AD vs control classification accuracy. Secondary: incremental benefit of adding acoustic features and targeted features to language-based models. |
| Results | Text-based CNN-LSTM models achieved around 72.92% accuracy on ADReSS (lower than earlier results on DementiaBank due to dataset balance and difficulty). The proposed Speech-GRU acoustic branch improved acoustic-only baselines by ~2%, and when enriched with targeted linguistic features, improved performance by ~6.25% over acoustic baselines, demonstrating advantages of the bimodal approach. |
| Missing Data Handling | Analyses confined to recordings with both audio and transcripts present in ADReSS; no explicit imputation strategy reported. |
| Risk of Bias / Limitations | Single dataset (ADReSS) with limited size; English-only, single task (picture description); potential overfitting of deep models; external validation not explored. |
| Funding / Conflicts | Conducted at BITS Pilani Cognitive Neuroscience Lab; authors did not report conflicts of interest. |

112

| Study ID | Mei et al., 2023 (The USTC system for ADReSS-M challenge) |
| --- | --- |
| Publication Year | 2023 |
| Country / Setting | China; cross-lingual dementia detection challenge (English training, Greek testing). |
| Study Design | Challenge system description for the ICASSP 2023 ADReSS-M multilingual Alzheimer’s dementia recognition task, exploring acoustic-feature-based models and fine-tuned wav2vec2.0 models for cross-lingual AD detection and MMSE prediction. |
| Datasets Used | ADReSS-M challenge dataset: English training set (237 samples) and Greek development/test sets (8 development, 46 test samples). |
| Recruitment Period | Defined by ADReSS-M organisers; speech recorded as part of picture description tasks in English and Greek. |
| Inclusion Criteria | Participants with spontaneous speech recordings for picture description in English or Greek with AD diagnosis and MMSE scores available. |
| Exclusion Criteria | Samples excluded by challenge organisers; no additional exclusions by authors. |
| Sample Size | Training: 237 English + 8 Greek samples; Test: 46 Greek samples. |
| Group Distribution | Binary AD vs control labels for classification task; continuous MMSE scores for regression task. |
| Participant Demographics | Adults and older adults; balanced for AD status, age, and gender in each split as per challenge description. |
| Modalities Used | Speech audio only (no text); multiple acoustic feature sets plus raw waveforms for wav2vec2.0. |
| Acquisition Details | Spontaneous picture description recordings; sampling rates and recording conditions per challenge documentation. |
| Preprocessing | Extraction of eGeMAPS and other acoustic features; design of silence-related features as language-independent cues; low-pass filtering of speech into different frequency bands (e.g., 0–1000 Hz) to emphasise prosodic information; preparation of inputs for XGBoost, SVR, and wav2vec2.0 fine-tuning. |
| Model Architecture | 1) Classical ML models (XGBoost classifier for AD detection, SVR and XGBoost for MMSE regression) trained on acoustic and silence features. 2) Bilingual wav2vec2.0 model pre-trained on English and Greek speech and fine-tuned for AD detection on different frequency bands. |
| Training & Validation | English and Greek training sets used with cross-validation for hyperparameter tuning; final models evaluated on Greek test set; some submissions trained on combined English + Greek validation data; performance reported for different feature combinations and frequency bands. |
| Outcomes | Primary: AD vs control classification accuracy on Greek test set. Secondary: MMSE regression RMSE and correlation, and contribution of silence features and low-frequency speech to cross-lingual transfer. |
| Results | Best AD detection performance achieved 73.9% accuracy on the Greek test set by fine-tuning a bilingual wav2vec2.0 model on 0–1000 Hz low-pass filtered speech. Best MMSE prediction reached RMSE 4.610 with correlation r = 0.565 using fused eGeMAPS and silence features. |
| Missing Data Handling | Challenge dataset largely complete; no discussion of imputation; models rely on available recordings and MMSE labels. |
| Risk of Bias / Limitations | Very small Greek development/test samples; specific to cross-lingual transfer from English to Greek picture description; speech-only (no text transcripts); clinical applicability beyond challenge conditions not evaluated. |
| Funding / Conflicts | Partially funded by the National Natural Science Foundation of China and China Postdoctoral Science Foundation; authors did not report conflicts of interest. |

122

| Study ID | Meerza et al., 2022 (Fair and Privacy-Preserving Alzheimer’s Disease Diagnosis Based on Spontaneous Speech Analysis via Federated Learning) |
| --- | --- |
| Publication Year | 2022 |
| Country / Setting | United States; federated learning framework evaluated on English ADReSS dataset. |
| Study Design | Method development study introducing a federated learning (FL) approach for automatic AD diagnosis from speech, with a focus on privacy preservation and fairness across clients with heterogeneous data. |
| Datasets Used | ADReSS challenge dataset (balanced English Cookie Theft speech recordings with AD vs control labels). |
| Recruitment Period | Original DementiaBank / ADReSS recruitment; secondary analysis only. |
| Inclusion Criteria | ADReSS participants with usable speech audio and AD vs control labels. |
| Exclusion Criteria | Recordings failing feature extraction or missing labels; no additional exclusions beyond ADReSS quality controls. |
| Sample Size | ADReSS balanced subset (156 recordings: 78 AD, 78 controls). |
| Group Distribution | Binary classification: AD vs healthy control. |
| Participant Demographics | Older English-speaking adults; dementia group with clinically diagnosed AD; controls cognitively normal; demographics as in ADReSS documentation. |
| Modalities Used | Speech audio features, decomposed into acoustic mel-frequency features and linguistic disfluency features (pause rate and duration). |
| Acquisition Details | Standard ADReSS microphone recordings of Cookie Theft picture descriptions; this study performs only secondary feature extraction and modelling. |
| Preprocessing | Extraction of mel-frequency features for acoustic representation; computation of pause-based linguistic features (pause rate and duration) from speech. Partition of data among simulated FL clients to mimic non-identically distributed datasets (heterogeneous client data). Normalisation of features per client/server as needed. |
| Model Architecture | Dual-subnetwork architecture: (1) LSTM network to process acoustic mel-frequency sequences; (2) feed-forward neural network to process pause-based features. Outputs fused for final AD vs control prediction. The model is trained under a federated learning setting using federated optimisers. |
| Training & Validation | Extensive experiments under various FL configurations: standard FedAvg, and fair aggregation mechanisms including q-FedAvg and q-FedSGD to mitigate client-level performance disparities. Centralised (non-FL) training serves as a performance upper bound. Evaluation uses classification accuracy and fairness metrics across clients. |
| Outcomes | Primary: AD vs control classification accuracy under federated learning while preserving data privacy (no raw speech uploaded to server). Secondary: model fairness across clients with heterogeneous data distributions, assessed via performance variation and fairness-aware objectives. |
| Results | The proposed FL framework achieved high AD detection accuracy close to centralised training baselines, demonstrating that effective speech-based AD screening can be achieved without centralising raw data. Fair aggregation methods (q-FedAvg, q-FedSGD) substantially reduced algorithmic bias and improved worst-case client performance compared with vanilla FedAvg. |
| Missing Data Handling | FL experiments conducted on complete ADReSS subset; clients with very small data simulated but not removed; missing data not a major issue as corpus is curated. |
| Risk of Bias / Limitations | Evaluation limited to a single small English dataset; FL clients are simulated rather than real devices; broader deployment issues (communication cost, on-device constraints) not fully explored; approach still relies on feature extraction from raw speech. |
| Funding / Conflicts | Supported by US National Science Foundation grants CNS-2114161, ECCS-2132106, and CBET-2130643; no conflicts of interest reported. |

114

| Study ID | Chen et al., 2023 (Cross-Lingual Alzheimer’s Disease Detection Based on Paralinguistic and Pre-Trained Features) |
| --- | --- |
| Publication Year | 2023 |
| Country / Setting | China; cross-lingual speech-based dementia challenge (ICASSP-SPGC 2023 ADReSS-M, English→Greek). |
| Study Design | Challenge system description for cross-lingual AD detection and MMSE prediction using paralinguistic acoustic features and multilingual pre-trained representations from speech and text. |
| Datasets Used | ADReSS-M challenge dataset: English training data and Greek test data comprising spontaneous picture description speech with AD labels and MMSE scores. |
| Recruitment Period | Defined by ADReSS-M organisers; no new data collected by authors. |
| Inclusion Criteria | ADReSS-M participants with valid English or Greek speech recordings and available labels (AD vs healthy control) and/or MMSE scores. |
| Exclusion Criteria | Samples excluded by challenge organisers; instances with unusable audio or missing task labels not used in training. |
| Sample Size | Training and development sets: 237 English samples; Greek data used only for development and final test (46 Greek test samples). |
| Group Distribution | Balanced AD vs control distributions within each official split as defined by ADReSS-M (approximately equal numbers of AD and healthy speakers). |
| Participant Demographics | Older adults; English and Greek speakers recruited for dementia research, balanced by age and gender across splits. |
| Modalities Used | 1) Paralinguistic acoustic features extracted from raw speech (IS10-Paralinguistics, IS10-Paralinguistics-compat, IS11-speaker-state via openSMILE). 2) Pre-trained acoustic embeddings from XLSR-53 (self-supervised multilingual wav2vec 2.0 model). 3) Linguistic features from automatic speech recognition (ASR) transcripts encoded by pre-trained language models. |
| Acquisition Details | Spontaneous Cookie Theft picture-description speech recorded in clinical/research environments in English and Greek as part of the ADReSS-M dataset; audio sampling and recording parameters fixed by the challenge organisers. |
| Preprocessing | Extraction of multiple paralinguistic feature sets using openSMILE; derivation of deep acoustic representations from XLSR-53; ASR transcription of speech to obtain text for linguistic feature extraction; standardisation and concatenation of feature vectors for downstream models. |
| Model Architecture | Several feature combinations evaluated: (1) paralinguistic-only, (2) XLSR-53-only, (3) fusion of paralinguistic + XLSR-53, and (4) fusion including linguistic features. Classifiers include SVMs and fully connected neural networks for AD detection, and regression models for MMSE prediction. |
| Training & Validation | Models trained on English data with cross-validation; final systems evaluated on the Greek test set. Experiments compare unimodal and multimodal feature sets and analyse which acoustic/linguistic features transfer best across languages. |
| Outcomes | Primary: cross-lingual AD vs healthy control classification accuracy on Greek test set. Secondary: MMSE score regression error (RMSE). |
| Results | The best-performing system combining paralinguistic and pre-trained features achieved 69.6% accuracy for AD detection and RMSE 4.788 for MMSE prediction on the Greek test set, showing that paralinguistic and multilingual pre-trained features can support cross-lingual AD detection. |
| Missing Data Handling | Challenge dataset largely complete; the study relied on samples with valid audio and MMSE labels; no complex imputation strategies reported. |
| Risk of Bias / Limitations | Very small Greek test set; cross-lingual transfer evaluated only for English-to-Greek; reliance on ASR quality for linguistic features; performance still below monolingual systems. |
| Funding / Conflicts | Supported by the National Key R&D Program of China and the National Natural Science Foundation of China; no conflicts of interest reported. |

106

| Study ID | Ilias et al., 2023 (Detecting dementia from speech and transcripts using transformers) |
| --- | --- |
| Publication Year | 2023 |
| Country / Setting | Greece; research on English spontaneous speech (ADReSS dataset). |
| Study Design | Method development study proposing uni- and multimodal transformer-based architectures for dementia detection from spontaneous speech, using speech-as-image encodings plus textual transcripts. |
| Datasets Used | ADReSS Challenge dataset (balanced AD vs control recordings from the DementiaBank Pitt Corpus). |
| Recruitment Period | Original DementiaBank recruitment; ADReSS subset used as defined by the challenge. |
| Inclusion Criteria | ADReSS recordings with available audio and transcripts for picture description tasks, labelled as AD or non-AD. |
| Exclusion Criteria | Samples with unusable audio or missing transcripts; data outside ADReSS subset. |
| Sample Size | Balanced ADReSS subset (approximately 156 recordings: 78 AD, 78 controls). |
| Group Distribution | Binary classification: AD vs non-AD control. |
| Participant Demographics | Older English-speaking adults, typical of DementiaBank (clinic and community participants). |
| Modalities Used | 1) Acoustic: speech encoded as images via log-Mel spectrograms and Mel-frequency cepstral coefficients (MFCCs), plus their delta and delta‑delta. 2) Textual: transcripts of spontaneous speech. |
| Acquisition Details | Audio from DementiaBank picture description tasks; manually produced transcripts. |
| Preprocessing | Conversion of waveforms into 3‑channel images (log-Mel or MFCC + delta + delta‑delta); extraction of frame-based features as inputs to pretrained vision backbones; tokenisation of transcripts for transformer-based language models; normalisation and padding/truncation. |
| Model Architecture | Unimodal audio: several pretrained CNN/vision backbones evaluated (AlexNet, VGG16, DenseNet, EfficientNet, Vision Transformer), with ViT giving best acoustic performance. Unimodal text: BERT-based transformer for transcript classification. Multimodal models with a Gated Multimodal Unit (GMU) controlling the contribution of each modality and crossmodal attention mechanisms to capture interactions between acoustic and textual representations. |
| Training & Validation | Supervised training on ADReSS with cross-validation; comparison of acoustic-only, text-only, and multimodal transformers; ablation of GMU and crossmodal attention; evaluation with accuracy and F1-score. |
| Outcomes | Primary: AD vs non-AD classification accuracy from spontaneous speech. Secondary: benefit of transformer-based encoders vs earlier CNN/LSTM models, and contribution of multimodal fusion. |
| Results | ViT-based acoustic models outperformed traditional acoustic feature + SVM pipelines. Textual BERT models provided strong baselines, and multimodal models with GMU and crossmodal attention achieved the highest performance, surpassing earlier state-of-the-art approaches on ADReSS (accuracy around the high 80s / low 90s, depending on setup). |
| Missing Data Handling | Only recordings with both audio and transcripts were used in multimodal models; no complex imputation reported. |
| Risk of Bias / Limitations | Single dataset; English-only picture description; modest sample size; focus on binary AD vs control rather than finer-grained staging; external generalisation not tested. |
| Funding / Conflicts | Supported by institutional research programmes in Greece; no conflicts of interest reported. |

113

| Study ID | Tamm et al., 2023 (Cross-lingual transfer learning for Alzheimer’s detection from spontaneous speech) |
| --- | --- |
| Publication Year | 2023 |
| Country / Setting | Belgium; cross-lingual dementia detection using English and Greek datasets (ADReSS-M). |
| Study Design | Challenge system paper describing a cross-lingual transfer-learning approach for AD detection and MMSE prediction, trained on English and adapted to Greek using mixed-language batches and parameter averaging. |
| Datasets Used | ADReSS-M challenge dataset: English picture-description recordings and Greek picture-description recordings, with AD labels and MMSE scores. |
| Recruitment Period | As defined by ADReSS-M organisers; no new data collected. |
| Inclusion Criteria | English training split (initially 237, reduced to 228 after balancing and removing missing scores) and Greek sample/test splits (8 + 46) with available labels and MMSE scores. |
| Exclusion Criteria | One English healthy control with missing cognitive score removed; 8 AD patients removed for class balancing; remaining samples used for training. |
| Sample Size | Final English training set: 228 samples (balanced AD vs control). Greek sample split: 8 subjects (4 AD). Greek test split: 46 subjects (22 AD). |
| Group Distribution | Binary AD vs control for classification; continuous MMSE scores for regression. |
| Participant Demographics | Older adults; splits balanced for AD diagnosis, age, and gender according to challenge specification. |
| Modalities Used | Speech acoustic features and covariates (age, gender, education) – no transcripts. |
| Acquisition Details | Spontaneous picture description tasks recorded in English and Greek; recordings supplied by challenge organisers. |
| Preprocessing | Extraction of frame-level acoustic features (e.g., ComParE or similar low-level descriptors); normalisation; concatenation with demographic covariates (age, gender, education). Data prepared for sequence models to handle temporal structure of speech. |
| Model Architecture | Sequence models (e.g., recurrent networks) that take sequences of acoustic feature frames and covariates to predict AD and MMSE. Cross-lingual transfer achieved via training on English data first, then adapting to Greek using mixed-language batches and parameter averaging across languages. |
| Training & Validation | Initial training entirely on English data; then mixed English–Greek training with progressive parameter averaging for cross-lingual adaptation; final evaluation on Greek test split. Performance measured with accuracy for AD detection and RMSE for MMSE prediction. |
| Outcomes | Primary: cross-lingual AD detection accuracy on Greek test set. Secondary: MMSE prediction RMSE and demonstration that features learned in English can transfer to Greek with limited Greek data. |
| Results | Cross-lingual model achieved 82.6% accuracy for AD detection on the Greek test set, and RMSE 4.345 for MMSE prediction, outperforming the ADReSS-M baseline (73.9% accuracy and RMSE 4.955). The system ranked 2nd overall among 24 competition entries. |
| Missing Data Handling | Subjects with missing cognitive scores removed from the English training set; no additional imputation methods described. |
| Risk of Bias / Limitations | Relies on small Greek sample; cross-lingual transfer evaluated only between English and Greek; uses acoustic features only; broader generalization to other languages and tasks remains untested. |
| Funding / Conflicts | Supported by KU Leuven Special Research Fund grant C24M/22/025; no conflicts of interest reported. |

118

| Study ID | Hlédiková, Woszczyk et al., 2022 (Data Augmentation for Dementia Detection in Spoken Language) |
| --- | --- |
| Publication Year | 2022 |
| Country / Setting | United Kingdom & Germany; dementia detection using English spontaneous speech (ADReSS dataset). |
| Study Design | Empirical evaluation of 17 label-preserving audio and text data augmentation techniques for Alzheimer’s disease detection, tested on both traditional ML and transformer-based models, in unimodal and multimodal settings. |
| Datasets Used | ADReSS dataset (balanced AD vs control Cookie Theft recordings with audio and transcripts). |
| Recruitment Period | Original DementiaBank / ADReSS recruitment; no new data collected. |
| Inclusion Criteria | ADReSS participants with available audio recordings and transcripts with AD vs control labels. |
| Exclusion Criteria | Corrupted or incomplete recordings; transcripts not conforming to ADReSS specifications. |
| Sample Size | ADReSS balanced training and test splits (156 recordings total: 78 AD, 78 controls). |
| Group Distribution | Binary classification: AD vs healthy controls. |
| Participant Demographics | Older English-speaking adults performing Cookie Theft picture description tasks; demographics as documented in ADReSS dataset. |
| Modalities Used | 1) Text transcripts. 2) Audio recordings. Augmentation applied separately and jointly to each modality. |
| Acquisition Details | Standard ADReSS microphone recordings and transcripts; authors do not alter acquisition, only augmentation of data. |
| Preprocessing | Text augmentations: sentence deletion, Easy Data Augmentation (EDA), Mixup, and several deep generative approaches. Audio augmentations: noise addition, speed and pitch perturbation, reverberation, time masking, and more. Features extracted for SVM/RF baselines and transformer-based models; normalisation applied as appropriate. |
| Model Architecture | Two main model families: (1) Transformer-based models for both text and audio domains, and (2) traditional ML baselines (SVM for text, Random Forest for audio). Multimodal fusion of text and audio representations also evaluated. |
| Training & Validation | Models trained on original vs augmented datasets; performance compared across augmentation strategies on held-out test data; label preservation assessed qualitatively and quantitatively. |
| Outcomes | Primary: AD vs control classification accuracy. Secondary: impact of specific augmentation techniques on model generalisation and label preservation for both modalities. |
| Results | Data augmentation improved performance for both text- and audio-based models and yielded accuracy comparable to state-of-the-art results on ADReSS using carefully crafted features and architectures. Selected augmentations preserved labels well and enhanced generalisation for transformer and traditional models alike. |
| Missing Data Handling | Work restricted to ADReSS recordings with complete audio and transcripts; no advanced handling of missing data required. |
| Risk of Bias / Limitations | Single English dataset focussed on a single speech task; augmentations tuned specifically for ADReSS; potential overfitting to challenge conditions; no clinical external validation. |
| Funding / Conflicts | Authors are affiliated with Imperial College London and University of Augsburg; no conflicts of interest reported in the paper. |

119

| Study ID | Jin et al., 2023 (CONSEN: Complementary and Simultaneous Ensemble for AD Detection and MMSE Score Prediction) |
| --- | --- |
| Publication Year | 2023 |
| Country / Setting | Korea; multilingual AD detection from spontaneous speech in the ICASSP 2023 ADReSS-M challenge. |
| Study Design | Challenge-winning system description proposing the CONSEN (Complementary and Simultaneous Ensemble) algorithm for joint AD detection and MMSE regression using multilingual acoustic and disfluency features. |
| Datasets Used | ADReSS-M challenge dataset: English training set and Greek development/test sets of Cookie Theft picture description recordings with AD labels and MMSE scores. |
| Recruitment Period | As defined by ADReSS-M organisers; no new recruitment. |
| Inclusion Criteria | Participants with multilingual (English/Greek) spontaneous speech, AD diagnosis status, and MMSE scores; recordings meeting challenge quality criteria. |
| Exclusion Criteria | Samples removed by challenge organisers due to missing labels or poor quality; no additional exclusions applied by authors. |
| Sample Size | English training set: 237 recordings; Greek development and test: 8 and 46 recordings, respectively. |
| Group Distribution | Balanced AD vs control distributions in each split, with MMSE variability in AD and control groups. |
| Participant Demographics | Adults and older adults; English and Greek speakers; groups balanced for age and gender across splits according to challenge description. |
| Modalities Used | Acoustic speech features (wav2vec, i-vector, x-vector, VGGish) and disfluency features derived from pauses and speaker interventions; no text transcripts used. |
| Acquisition Details | Spontaneous Cookie Theft picture description recordings; audio contains both interviewer and participant speech. |
| Preprocessing | Automatic audio segmentation into voice and pause sections, followed by speaker diarisation to distinguish participant vs interviewer segments. Construction of symbol sequences (P, I, S) representing participant, interviewer, and silence. Extraction of 18 disfluency features (e.g., pause ratios, number of interventions, statistics of pause and voice segments). Extraction of multiple acoustic embedding types (wav2vec, i-vector, x-vector, VGGish). |
| Model Architecture | CONSEN algorithm performs a complementary and simultaneous ensemble of multiple base learners: different acoustic feature models and disfluency-feature-based models for both AD detection and MMSE prediction. Ensemble weights are optimised to improve both tasks jointly. |
| Training & Validation | Models trained on English and Greek subsets; ensemble tuned using development data. Final performance evaluated on Greek test set; comparison of individual feature-model combinations vs CONSEN ensemble. |
| Outcomes | Primary: AD vs control classification accuracy. Secondary: MMSE score prediction (RMSE) and demonstration that complementary feature ensembles improve multilingual performance. |
| Results | The proposed method achieved first place in both tasks of the ADReSS-M challenge, with best AD detection accuracy of 86.69% and MMSE RMSE of 3.727. Results demonstrate that combining acoustic embeddings with carefully engineered disfluency features via CONSEN yields highly accurate multilingual AD detection and MMSE estimation. |
| Missing Data Handling | Challenge dataset largely complete; analyses focus on samples with full speech and MMSE information; no sophisticated imputation methods discussed. |
| Risk of Bias / Limitations | Evaluation limited to English–Greek ADReSS-M dataset; reliance on diarisation quality for disfluency features; ensemble complexity may pose deployment challenges; no evaluation on non-challenge real-world clinical cohorts. |
| Funding / Conflicts | Supported by Konkuk University and Voinosis Inc.; no conflicts of interest reported. |
