# Supplementary material for: Multimodal AI for Alzheimer Disease Diagnosis: Systematic Review of Datasets, Models, and Modalities
Source: J Med Internet Res. 2026 Mar 25;28:e85414. doi: 10.2196/85414 (PMC13018777; doi:10.2196/85414)
Supplement: Checklist 1 — Completed PRISMA 2020, PRISMA-S checklist, and PRISMA expanded checklist specifying reporting locations for all required items, including eligibility criteria, search methods, extraction procedures, bias assessments, and synthesis reporting. PRISMA: Preferred Reporting Items for Systematic Reviews and Meta-Analyses; PRISMA-S: Preferred Reporting Items for Systematic Reviews and Meta-Analyses extension for literature searches. [file jmir-v28-e85414-s006.pdf]

PRISMA 2020 Checklist

Section and Topic	Item #	Checklist item	Location where item is reported
**TITLE**			
Title	1	Identify the report as a systematic review.	Manuscript page 1
**ABSTRACT**			
Abstract	2	See the PRISMA 2020 for Abstracts checklist.	Manuscript pages 1–2
**INTRODUCTION**			
Rationale	3	Describe the rationale for the review in the context of existing knowledge.	Manuscript pages 2–3
Objectives	4	Provide an explicit statement of the objective(s) or question(s) the review addresses.	Manuscript page 3
**METHODS**			
Eligibility criteria	5	Specify the inclusion and exclusion criteria for the review and how studies were grouped for the syntheses.	Manuscript pages 4–5 and Appendix pages 1–6
Information sources	6	Specify all databases, registers, websites, organisations, reference lists and other sources searched or consulted to identify studies. Specify the date when each source was last searched or consulted.	Manuscript page 4
Search strategy	7	Present the full search strategies for all databases, registers and websites, including any filters and limits used.	Manuscript page 4; Multimedia Appendix 1 pages 1 – 6
Selection process	8	Specify the methods used to decide whether a study met the inclusion criteria of the review, including how many reviewers screened each record and each report retrieved, whether they worked independently, and if applicable, details of automation tools used in the process.	Manuscript pages 5–6
Data collection process	9	Specify the methods used to collect data from reports, including how many reviewers collected data from each report, whether they worked independently, any processes for obtaining or confirming data from study investigators, and if applicable, details of automation tools used in the process.	Manuscript page 5
Data items	10a	List and define all outcomes for which data were sought. Specify whether all results that were compatible with each outcome domain in each study were sought (e.g. for all measures, time points, analyses), and if not, the methods used to decide which results to collect.	Manuscript pages 7–8
	10b	List and define all other variables for which data were sought (e.g. participant and intervention characteristics, funding sources). Describe any assumptions made about any missing or unclear information.	Manuscript pages 13–14
Study risk of bias assessment	11	Specify the methods used to assess risk of bias in the included studies, including details of the tool(s) used, how many reviewers assessed each study and whether they worked independently, and if applicable, details of automation tools used in the process.	Manuscript page 9 + Figure 3 on page 10
Effect measures	12	Specify for each outcome the effect measure(s) (e.g. risk ratio, mean difference) used in the synthesis or presentation of results.	Manuscript pages 7–8
Synthesis methods	13a	Describe the processes used to decide which studies were eligible for each synthesis (e.g. tabulating the study intervention characteristics and comparing against the planned groups for each synthesis (item #5)).	Manuscript page 13
	13b	Describe any methods required to prepare the data for presentation or synthesis, such as handling of missing summary statistics, or data conversions.	Manuscript page 36
	13c	Describe any methods used to tabulate or visually display results of individual studies and syntheses.	Manuscript pages 6–38
	13d	Describe any methods used to synthesize results and provide a rationale for the choice(s). If meta-analysis was performed, describe the model(s), method(s) to identify the presence and extent of statistical heterogeneity, and software package(s) used.	Manuscript pages 37–38
	13e	Describe any methods used to explore possible causes of heterogeneity among study results (e.g. subgroup analysis, meta-regression).	Manuscript page 37

Section and Topic	Item #	Checklist item	Location where item is reported
	13f	Describe any sensitivity analyses conducted to assess robustness of the synthesized results.	Manuscript pages 36–37
Reporting bias assessment	14	Describe any methods used to assess risk of bias due to missing results in a synthesis (arising from reporting biases).	Manuscript pages 9-10,
Certainty assessment	15	Describe any methods used to assess certainty (or confidence) in the body of evidence for an outcome.	Manuscript pages 44–45
**RESULTS**			
Study selection	16a	Describe the results of the search and selection process, from the number of records identified in the search to the number of studies included in the review, ideally using a flow diagram.	Manuscript page 5 + Figure 1 on Manuscript page 6
	16b	Cite studies that might appear to meet the inclusion criteria, but which were excluded, and explain why they were excluded.	Manuscript page 5
Study characteristics	17	Cite each included study and present its characteristics.	Manuscript pages 22–36
Risk of bias in studies	18	Present assessments of risk of bias for each included study.	Manuscript page 10 (Figure 3) + page 9; Multimedia Appendix 2 pages 1–4
Results of individual studies	19	For all outcomes, present, for each study (a) summary statistics for each group (where appropriate) and (b) an effect estimate and its precision (e.g. confidence/credible interval), ideally using structured tables or plots.	Manuscript pages 22–36
Results of syntheses	20a	For each synthesis, briefly summarise the characteristics and risk of bias among contributing studies.	Manuscript pages 37–38
	20b	Present results of all statistical syntheses conducted. If meta-analysis was done, present for each the summary estimate and its precision (e.g. confidence/credible interval) and measures of statistical heterogeneity. If comparing groups, describe the direction of the effect.	Manuscript pages 37–38
	20c	Present results of all investigations of possible causes of heterogeneity among study results.	Manuscript page 37
	20d	Present results of all sensitivity analyses conducted to assess the robustness of the synthesized results.	Manuscript page 37
Reporting biases	21	Present assessments of risk of bias due to missing results (arising from reporting biases) for each synthesis assessed.	Manuscript pages 44–45
Certainty of evidence	22	Present assessments of certainty (or confidence) in the body of evidence for each outcome assessed.	Manuscript pages 32–35 and 21–29
**DISCUSSION**			
Discussion	23a	Provide a general interpretation of the results in the context of other evidence.	Manuscript pages 39–40
	23b	Discuss any limitations of the evidence included in the review.	Manuscript pages 40–46
	23c	Discuss any limitations of the review processes used.	Manuscript pages 40–46
	23d	Discuss implications of the results for practice, policy, and future research.	Manuscript pages 40–46
**OTHER INFORMATION**			
Registration and protocol	24a	Provide registration information for the review, including register name and registration number, or state that the review was not registered.	Manuscript page 5
	24b	Indicate where the review protocol can be accessed, or state that a protocol was not prepared.	Manuscript page 1
	24c	Describe and explain any amendments to information provided at registration or in the protocol.	Manuscript page 5

PRISMA 2020 Checklist

Section and Topic	Item #	Checklist item	Location where item is reported
Support	25	Describe sources of financial or non-financial support for the review, and the role of the funders or sponsors in the review.	Manuscript page 47
Competing interests	26	Declare any competing interests of review authors.	Manuscript page 47
Availability of data, code and other materials	27	Report which of the following are publicly available and where they can be found template data collection forms; data extracted from included studies; data used for all analyses; analytic code; any other materials used in the review.	Manuscript page 47

From Page MJ, McKenzie JE, Bossuyt PM, Boutron I, Hoffmann TC, Mulrow CD, et al. The PRISMA 2020 statement an updated guideline for reporting systematic reviews. BMJ 2021;372 n71. doi 10.1136/bmj.n71. This work is licensed under CC BY 4.0. To view a copy of this license, visit https //creativecommons.org/licenses/by/4.0/

PRISMA-S Checklist

Section/topic	#	Checklist item	Location(s) Reported
**INFORMATION SOURCES AND METHODS**			
Database name	1	Name each individual database searched, stating the platform for each.	Manuscript page 4
Multi-database searching	2	If databases were searched simultaneously on a single platform, state the name of the platform, listing all of the databases searched.	Manuscript page 4
Study registries	3	List any study registries searched.	Manuscript page 4
Online resources and browsing	4	Describe any online or print source purposefully searched or browsed (e.g., tables of contents, print conference proceedings, web sites), and how this was done.	Manuscript page 4
Citation searching	5	Indicate whether cited references or citing references were examined, and describe any methods used for locating cited/citing references (e.g., browsing reference lists, using a citation index, setting up email alerts for references citing included studies).	Manuscript page 4
Contacts	6	Indicate whether additional studies or data were sought by contacting authors, experts, manufacturers, or others.	Manuscript page 4
Other methods	7	Describe any additional information sources or search methods used.	Manuscript page 4 and Appendix 1 pages 1–6
SEARCH STRATEGIES			
Full search strategies	8	Include the search strategies for each database and information source, copied and pasted exactly as run.	Manuscript page 4 and Appendix 1 pages 1–6
Limits and restrictions	9	Specify that no limits were used, or describe any limits or restrictions applied to a search (e.g., date or time period, language, study design) and provide justification for their use.	Manuscript page 5 and Appendix 1 pages 1–6
Search filters	10	Indicate whether published search filters were used (as originally designed or modified), and if so, cite the filter(s) used.	Manuscript page 4

Prior work	11	Indicate when search strategies from other literature reviews were adapted or reused for a substantive part or all of the search, citing the previous review(s).	Manuscript page 4
Updates	12	Report the methods used to update the search(es) (e.g., rerunning searches, email alerts).	Manuscript page 5
Dates of searches	13	For each search strategy, provide the date when the last search occurred.	Manuscript page 5 and Appendix 1 pages 1–6
PEER REVIEW			
Peer review	14	Describe any search peer review process.	Manuscript page 4
MANAGING RECORDS			
Total Records	15	Document the total number of records identified from each database and other information sources.	Manuscript page 5
Deduplication	16	Describe the processes and any software used to deduplicate records from multiple database searches and other information sources.	Manuscript page 5

PRISMA-S An Extension to the PRISMA Statement for Reporting Literature Searches in Systematic Reviews
Rethlefsen ML, Kirtley S, Waffenschmidt S, Ayala AP, Moher D, Page MJ, Koffel JB, PRISMA-S Group.
Last updated February 27, 2020.

PRISMA 2020 Checklist

PRISMA 2020 expanded checklist

Note: This expanded checklist details elements recommended for reporting for each item in the PRISMA 2020 statement. Non-italicized elements are considered 'essential' and should be reported n the main report or as supplementary material for all systematic reviews (except for those preceded by "If…", which should only be reported where applicable). Elements written in italics are additional', and while not essential, provide supplementary information that may enhance the completeness and usability of systematic review reports. Note that elements presented here are an abridged version of those presented in the explanation and elaboration paper (BMJ 2021;372:n160), with references and some examples removed. Consulting the explanation and elaboration paper s recommended if further clarity or information is required.

Section and Topic	Item #	Items and elements recommended for reporting
**TITLE**		
**TITLE**	1	Item: Identify the report as a systematic review.<br>Elements:<br>• The title clearly identifies the report as a systematic review and specifies the focus on multimodal artificial intelligence approaches for Alzheimer' s disease diagnosis.
**ABSTRACT**		
**ABSTRACT**	2	**Item: See the PRISMA 2020 for Abstracts checklist.**<br>Elements:<br>• The abstract was structured in accordance with the PRISMA 2020 for Abstracts checklist, reporting the background, objectives, data sources, eligibility crite methods, main results, limitations, and conclusions of the systematic review.
**INTRODUCTION**		
**RATIONALE**	3	**Item: Describe the rationale for the review in the context of existing knowledge.**<br>Elements:<br>• The rationale for this review is presented in the Introduction, which outlines current limitations in unimodal Alzheimer' s disease research, the fragmentation of existing multimodal studies, and the need for a unified synthesis to evaluate multimodal AI approaches across datasets, modalities, and clinical contexts.
**OBJECTIVES**	4	**Item: Provide an explicit statement of the objective(s) or question(s) the review addresses.**<br>Elements:<br>• This review aims to systematically evaluate multimodal AI-based approaches for Alzheimer' s disease by comparing datasets, modality combinations, modelling strategies, and diagnostic performance, and by identifying methodological strengths, limitations, and research gaps.
**METHODS**		
**ELIGIBILITY CRITERIA**	5	**Item: Specify the inclusion and exclusion criteria for the review and how studies were grouped for the syntheses.**<br>Elements:<br>• Eligibility criteria were defined in the Methods section, specifying inclusion and exclusion based on study population (Alzheimer' s disease or related cognitive impairment), use of multimodal data, application of AI or machine learning methods, reported quantitative outcomes, publication language, and study type.

PRISMA 2020 Checklist

Section and Topic	Item #	Items and elements recommended for reporting
INFORMATION SOURCES	6	**Item: Specify all databases, registers, websites, organisations, reference lists and other sources searched or consulted to identify studies. Specify the date when each source was last searched or consulted.**<br>Elements:<br>• Multiple information sources were searched, including PubMed, Scopus, IEEE Xplore, ACM Digital Library, Cochrane Library, and arXiv, with the date of last search for each database reported in the Methods section.
SEARCH STRATEGY	7	**Item: Present the full search strategies for all databases, registers and websites, including any filters and limits used.**<br>Element:<br>• Full search strategies, including database-specific search strings, filters, and date limits, are reported in the Methods section and detailed in Multimedia Appendix 1, with searches conducted across multiple databases using structured keyword combinations.
SELECTION PROCESS	8	**Item: Specify the methods used to decide whether a study met the inclusion criteria of the review, including how many reviewers screened each record and each report retrieved, whether they worked independently, and if applicable, details of automation tools used in the process.**<br>Elements:<br>• Study selection was performed through title/abstract screening followed by full-text review, with records screened independently and disagreements resolved consensus, and the overall process summarised in the PRISMA flow diagram (Figure 1).
DATA COLLECTION PROCESS	9	**Item: Specify the methods used to collect data from reports, including how many reviewers collected data from each report, whether they worked independently, any processes for obtaining or confirming data from study investigators, and if applicable, details of automation tools used in the process.**<br>Elements:<br>• Data were extracted independently by reviewers using a predefined extraction framework, with discrepancies resolved by discussion, and study characteristics and outcomes recorded systematically for synthesis.
DATA ITEMS (outcomes)	10a	**Item: List and define all outcomes for which data were sought. Specify whether all results that were compatible with each outcome domain in each study were sought (e.g. for all measures, time points, analyses), and if not, the methods used to decide which results to collect.**<br>Elements:<br>• Outcome data extracted included diagnostic, prognostic, and risk prediction performance measures (e.g., accuracy, AUC, F1-score, sensitivity, specificity), with all reported outcomes within each study collected where available and summarised across datasets in the Results section.
DATA ITEMS (other variables)	10b	**Item: List and define all other variables for which data were sought (e.g. participant and intervention characteristics, funding sources). Describe any assumptions made about any missing or unclear information.**<br>Elements:<br>• Additional data items extracted included study characteristics, participant demographics, data modalities, modelling approaches, and dataset sources, with assumptions regarding missing or unclear information documented during data extraction as described in the Methods section.
STUDY RISK OF BIAS ASSESSMENT	11	**Item: Specify the methods used to assess risk of bias in the included studies, including details of the tool(s) used, how many reviewers assessed each study and whether they worked independently, and if applicable, details of automation tools used in the process.**<br>Elements:<br>• Risk of bias for each included study was assessed using the QUADAS-2 tool, with domain-level judgements made independently and summarised in the Risk of Bias section and Figure 3.

PRISMA

PRISMA 2020 Checklist

Section and Topic	Item #	Items and elements recommended for reporting
EFFECT MEASURES	12	**Item: Specify for each outcome the effect measure(s) (e.g. risk ratio, mean difference) used in the synthesis or presentation of results.**<br>Elements:<br>• Effect measures reported for each outcome included classification and prediction performance metrics such as accuracy, AUC, sensitivity, specificity, and F1-score, as extracted and summarised in the Results section.
SYNTHESIS METHODS (eligibility for synthesis)	13a	**Item: Describe the processes used to decide which studies were eligible for each synthesis (e.g. tabulating the study intervention characteristics and comparing against the planned groups for each synthesis (item #5)).**<br>Element:<br>• Eligibility for each synthesis was determined by grouping studies according to data modality, clinical task, and modelling strategy, as specified in the Methods, and synthesised accordingly in the Results section.
SYNTHESIS METHODS (preparing for synthesis)	13b	**Item: Describe any methods required to prepare the data for presentation or synthesis, such as handling of missing summary statistics, or data conversions.**<br>Element:<br>• Data were prepared for synthesis by harmonising outcome measures and extracting comparable performance metrics across studies, with missing or non-comparable information handled through descriptive summarisation rather than statistical transformation.
SYNTHESIS METHODS (tabulation and graphical methods)	13c	**Item: Describe any methods used to tabulate or visually display results of individual studies and syntheses.**<br>Elements:<br>• Tabular and graphical presentations were used to summarise study characteristics and performance metrics, with tables and figures organised by dataset type and modelling approach to support comparative interpretation.
SYNTHESIS METHODS (statistical synthesis methods)	13d	**Item: Describe any methods used to synthesize results and provide a rationale for the choice(s). If meta-analysis was performed, describe the model(s), method(s) to identify the presence and extent of statistical heterogeneity, and software package(s) used.**<br>Elements:<br>• Formal meta-analysis was not conducted because of substantial heterogeneity in study designs, outcome definitions, and performance metrics; instead, a narrative synthesis approach was used to summarise and compare findings across studies.
SYNTHESIS METHODS (methods to explore heterogeneity)	13e	**Item: Describe any methods used to explore possible causes of heterogeneity among study results (e.g. subgroup analysis, meta-regression).**<br>Elements:<br>• Heterogeneity was explored qualitatively through comparison of study characteristics and outcomes across datasets and modelling approaches, as quantitative subgroup or meta-regression analyses were not appropriate.
SYNTHESIS METHODS (sensitivity analyses)	13f	**Item: Describe any sensitivity analyses conducted to assess robustness of the synthesized results.**<br>Elements:<br>• Sensitivity analyses were not undertaken because the heterogeneity in study designs, outcome measures, and modelling frameworks prevented meaningful quantitative reanalysis; robustness was instead evaluated qualitatively by examining consistency of findings across datasets and methodological settings.
REPORTING BIAS ASSESSMENT	14	**Item: Describe any methods used to assess risk of bias due to missing results in a synthesis (arising from reporting biases).**<br>Elements:<br>• Risk of bias due to missing results was assessed qualitatively using QUADAS-2 and narrative evaluation of reporting completeness across studies, with consideration of selective reporting and dataset availability described in the Methods and Discussion sections.

PRISMA 2020 Checklist

Section and Topic	Item #	Items and elements recommended for reporting
**CERTAINTY ASSESSMENT**	**15**	**Item: Describe any methods used to assess certainty (or confidence) in the body of evidence for an outcome.**<br>Elements:<br>• Certainty of evidence was assessed qualitatively using the QUADAS-2 framework, considering risk of bias, dataset heterogeneity, and consistency of findings across studies, with judgments reported in the *Risk of Bias and Quality Assessment* section and interpreted in the *Discussion*.
**RESULTS**		
**STUDY SELECTION (flow of studies)**	**16a**	**Item: Describe the results of the search and selection process, from the number of records identified in the search to the number of studies included in the review, ideally using a flow diagram.**<br>Elements:<br>• The study selection process, including numbers of records identified, screened, excluded, and included, is reported in the Methods section and summarised using a PRISMA flow diagram (Figure 1).
**STUDY SELECTION (excluded studies)**	**16b**	**Item: Cite studies that might appear to meet the inclusion criteria, but which were excluded, and explain why they were excluded.**<br>Element:<br>• Studies that appeared to meet the inclusion criteria but were ultimately excluded are reported in the study selection process and summarised in the PRISMA flow diagram (Figure 1), with reasons for exclusion provided.
**STUDY CHARACTERISTICS**	**17**	**Item: Cite each included study and present its characteristics.**<br>Elements:<br>• Characteristics of all included studies, including datasets, modalities, sample sizes, tasks, and evaluation metrics, are reported in the Results section and summarised in Tables 3 – 5 to facilitate comparison across studies.
**RISK OF BIAS IN STUDIES**	**18**	**Item: Present assessments of risk of bias for each included study.**<br>Elements:<br>• Risk of bias for each included study was assessed using the QUADAS-2 tool and summarised in the *Risk of Bias and Quality Assessment* section, with domain-level judgements presented visually in Figure 3.
**RESULTS OF INDIVIDUAL STUDIES**	**19**	**Item: For all outcomes, present, for each study: (a) summary statistics for each group (where appropriate) and (b) an effect estimate and its precision (e.g. confidence/credible interval), ideally using structured tables or plots.**<br>Elements:<br>• For each included study, summary characteristics and outcome measures (e.g., accuracy, AUC, F1-score) are reported in the Results section and presented in structured tables (Tables 3 – 5), with effect estimates and performance metrics extracted directly from the original studies.
**RESULTS OF SYNTHESES**<br><br>**(characteristics of contributing studies)**	**20a**	**Item: For each synthesis, briefly summarise the characteristics and risk of bias among contributing studies.**<br>Elements:<br>• Characteristics and risk of bias of studies contributing to each synthesis are summarised in the Results section, particularly in the dataset-specific summaries (Tables 3 – 5) and the QUADAS-2 risk-of-bias assessment (Figure 3).

Section and Topic	Item #	Items and elements recommended for reporting
RESULTS OF SYNTHESES (results of statistical syntheses)	20b	**Item: Present results of all statistical syntheses conducted. If meta-analysis was done, present for each the summary estimate and its precision (e.g. confidence/credible interval) and measures of statistical heterogeneity. If comparing groups, describe the direction of the effect.**<br>Elements:<br>• Statistical synthesis results are reported descriptively across dataset-specific summaries in the Results section (Tables 3 – 5), including performance metrics such as accuracy, AUC, and F1-score, as formal meta-analysis with pooled effect sizes was not conducted due to substantial methodological heterogeneity.
RESULTS OF SYNTHESES (results of investigations of heterogeneity)	20c	**Item: Present results of all investigations of possible causes of heterogeneity among study results.**<br>Elements:<br>• Heterogeneity across studies was explored qualitatively by comparing dataset types, modalities, and modelling approaches in the Results section, with further interpretation in the Discussion, as formal statistical subgroup or meta-regression analyses were not feasible.
RESULTS OF SYNTHESES (results of sensitivity analyses)	20d	**Item: Present results of all sensitivity analyses conducted to assess the robustness of the synthesized results.**<br>Elements:<br>• Formal sensitivity analyses were not performed due to substantial heterogeneity across studies; however, robustness of the synthesized findings was qualitatively evaluated by comparing results across datasets, modalities, and modelling approaches in the Results and Discussion sections.
REPORTING BIASES	21	**Item: Present assessments of risk of bias due to missing results (arising from reporting biases) for each synthesis assessed.**<br>Elements:<br>• Risk of bias due to missing results was assessed qualitatively through QUADAS-2 evaluation and discussion of reporting completeness and dataset heterogeneity in the Risk of Bias and Quality Assessment and Discussion sections, as formal funnel plot or quantitative publication bias analyses were not feasible due to methodological heterogeneity.
CERTAINTY OF EVIDENCE	22	**Item: Present assessments of certainty (or confidence) in the body of evidence for each outcome assessed.**<br>Elements:<br>• Certainty of evidence was qualitatively assessed using QUADAS-2 in the Risk of Bias and Quality Assessment section and further contextualised through discussion of dataset heterogeneity and external validation in the Discussion section.
**DISCUSSION**		
DISCUSSION (interpretation)	23a	**Item: Provide a general interpretation of the results in the context of other evidence.**<br>Element:<br>• Results are interpreted in the context of existing evidence on multimodal AI for Alzheimer's disease in the Discussion section (1 – 3 paragraphs)
DISCUSSION (limitations of evidence)	23b	**Item: Discuss any limitations of the evidence included in the review.**<br>Element:<br>• Limitations of the included evidence, including dataset heterogeneity, reliance on benchmark cohorts, and limited external validation, are discussed in the Discussion section.

PRISMA

PRISMA 2020 Checklist

Section and Topic	Item #	Items and elements recommended for reporting
**DISCUSSION (limitations of review processes)**	**23c**	**Item: Discuss any limitations of the review processes used.**<br>Element:<br>• Limitations of the review process, including exclusion of non-English literature, heterogeneity preventing meta-analysis, and reliance on reported performance metrics, are acknowledged in the Discussion.
**DISCUSSION (implications)**	**23d**	**Item: Discuss implications of the results for practice, policy, and future research.**<br>Elements:<br>• Implications for clinical practice, policy, and future research are discussed, emphasising interpretability, generalisability, and the need for standardised multimodal benchmarks.
**OTHER INFORMATION**		
**REGISTRATION AND PROTOCOL (registration)**	**24a**	This review was pre-registered at PROSPERO<br>Element:<br>• Registered ID: CRD420251241895
**REGISTRATION AND PROTOCOL (protocol)**	**24b**	This review was pre-registered at PROSPERO<br>Element:<br>• Link: https://www.crd.york.ac.uk/PROSPERO/recorddashboard, ID: CRD420251241895
**REGISTRATION AND PROTOCOL (amendments)**	**24c**	Add my second superviser<br>• Add my second superviser.
**SUPPORT**	**25**	"Support" section<br>Elements:<br>• This work received no specific financial or non-financial support. No funder or sponsor had any role in the design of the review; data collection, analysis, or interpretation; writing of the manuscript; or the decision to submit for publication.
**COMPETING INTERESTS**	**26**	Conflicts of Ineterst<br>Elements:<br>• No conflicts
**AVAILABILITY OF DATA, CODE, AND OTHER MATERIALS**	**27**	Availability of data, code, and other materials<br>Elements:<br>• **Template data collection / extraction form:** provided as Multimedia Appendix 4 (Cochrane Handbook Section 5.3.3 Extraction Framework), submitted with the manuscript.<br>**Data extracted from included studies:** extracted study characteristics and performance metrics are reported in the main evidence tables (eg, Tables 3 – 4; and your quantitative summary tables where applicable).<br>**Data used for all analyses:** the review did **not generate any new datasets**; all analysed data were extracted from publicly available publications included in the review<br>**Other materials used in the review:** full search strategies (Multimedia Appendix 1), QUADAS-2 risk-of-bias tables (Multimedia Appendix 2), traditional ML algorithm list (Multimedia Appendix 3), PRISMA & PRISMA-S checklists (Multimedia Appendix 5) — all submitted as supplementary appendices with the manuscript.
